# One-pot synthesis of substituted pyrrolo[3,4-*b*]pyridine-4,5-diones based on the reaction of *N*-(1-(4-hydroxy-6-methyl-2-oxo-2*H*-pyran-3-yl)-2-oxo-2-arylethyl)acetamide with amines

**DOI:** 10.3762/bjoc.15.277

**Published:** 2019-11-25

**Authors:** Valeriya G Melekhina, Andrey N Komogortsev, Boris V Lichitsky, Vitaly S Mityanov, Artem N Fakhrutdinov, Arkady A Dudinov, Vasily A Migulin, Yulia V Nelyubina, Elizaveta K Melnikova, Michail M Krayushkin

**Affiliations:** 1 N. D. Zelinsky Institute of Organic Chemistry, Russian Academy of Sciences, Leninsky Pr., 47, Moscow 119991, Russian Federation; 2 Department of Fine Organic Synthesis and Chemistry of Dyes, D. Mendeleyev University of Chemical Technology of Russia, Miusskaya Sq., 9, Moscow 125047, Russian Federation; 3 A. N. Nesmeyanov Institute of Organoelement Compounds, Vavilova St., 28, Moscow 119991, Russian Federation; 4 Lomonosov Moscow State University, Moscow 119991, Russian Federation

**Keywords:** condensation, dihydropyrrolone derivative, one-pot synthesis, pyrrolo[3,4-*b*]pyridine-4,5-diones, recyclization

## Abstract

The condensation of primary amines with *N*-(1-(4-hydroxy-6-methyl-2-oxo-2*H*-pyran-3-yl)-2-oxo-2-arylethyl)acetamides was explored. Thus, a previously unknown recyclization of the starting material was observed in acidic ethanol in the presence of an amine, which provided the corresponding dihydropyrrolone derivative as the major reaction product. Based on this transformation, a practical and convenient one-pot synthetic method for substituted pyrrolo[3,4-*b*]pyridin-5-ones could be devised.

## Introduction

Derivatives of pyrrolo[3,4-*b*]pyridin-5-one are known for their broad-spectrum biological activity [1–3]. One example includes a family of compounds based on this fragment that was tested as dipeptidyl peptidase-4 (DPP4) inhibitors [4–7]. From the obtained results, the authors suggested these compounds as effective therapeutic agents for the treatment of diabetes mellitus. Further, it was shown that several synthetic molecules with a pyrrolo[3,4-*b*]pyridin-5-one core display a remarkable anti-epileptic activity [8]. Additionally, compounds containing the 1*H*-imidazo[1’2’:1,2]pyrrolo[3,4-*b*]pyridine moiety were employed as herbicides [9]. At the same time, the published synthetic procedures towards the reported scaffolds were mainly specific for the individual products, with no universal methodology being presented. This limits the synthetic scope to targets carrying only certain, specific substituents. Therefore, considering the importance of the pyrrolo[3,4-*b*]pyridin-5-one motif, a novel, general synthetic approach to this class of organic compounds has become strongly desirable.

**Scheme 1:**
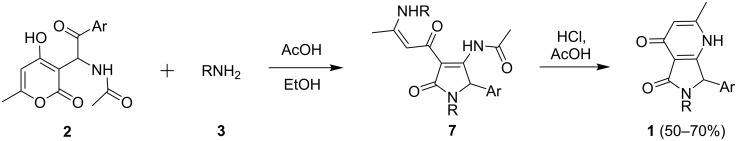
General synthetic pathway to **1**.

In this article, we describe a convenient synthetic method for the preparation of 6-alkyl-2-methyl-7-aryl-6,7-dihydro-1*H*-pyrrolo[3,4-*b*]pyridine-4,5-diones **1**. Therein, the condensation of *N*-(1-(4-hydroxy-6-methyl-2-oxo-2*H*-pyran-3-yl)-2-oxo-2-arylethyl)acetamides **2** with a diverse range of amines **3** did not only furnish the desired scaffolds in satisfactory yields but also allowed to use a variety of readily available substituted substrates (Scheme 1).

## Results and Discussion

**Scheme 2:**
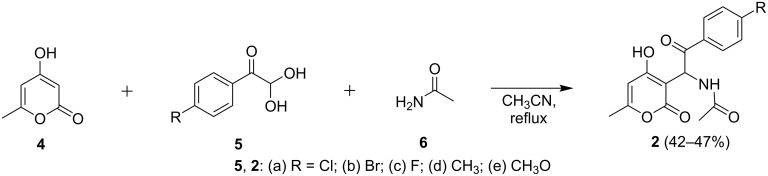
Synthesis of acetamides **2**.

The published synthesis of acetamides **2** includes a one-pot three-component reaction of 4-hydroxy-6-methyl-2*H*-pyran-2-one (**4**), arylglyoxals **5**, and acetamide (**6**) using SnCl_2_–SiO_2_ nanoparticles as heterogeneous catalyst under solvent-free conditions [10]. However, as we found out, the use of a catalyst was unnecessary for this transformation. In turn, refluxing the reaction mixture in acetonitrile was sufficient for this reaction to proceed, furnishing target materials **2** in good yields (Scheme 2).

The established straightforward synthesis of acetamides **2** provides further access to target materials with diverse substituent patterns. It is known that in the presence of amines, pyranones undergo recyclization under formation of various nitrogen-containing heterocycles [11–16]. In order to investigate the possibility of an analogous rearrangement of the pyranone ring in **2** when being attached by an amine, we studied the model reaction of *N*-(2-(4-chlorophenyl)-1-(4-hydroxy-6-methyl-2-oxo-2*H*-pyran-3-yl)-2-oxoethyl)acetamide (**2a**) with 2-(4-methoxyphenyl)ethan-1-amine (**3a**) under different conditions, and the results are summarized in Table 1.

**Table 1:** Screening and optimization of the reaction conditions for the synthesis of **7a**.^a^

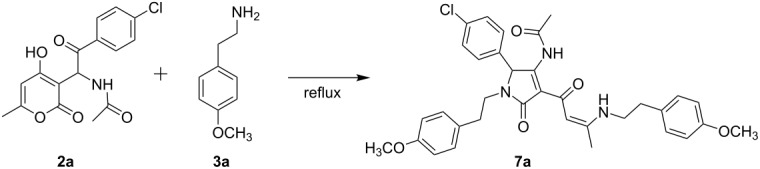				

Entry	Solvent	*t*, h	Acid (3 equiv)	Yield, %

1	MeCN	4	–	traces
2	MeCN	4	AcOH	39
3	MeCN	8	AcOH	22
4	dioxane	4	–	traces
5	dioxane	4	AcOH	25
6	dioxane	8	AcOH	13
7	EtOH	4	–	traces
8	EtOH	4	AcOH	60
9	EtOH	8	AcOH	24

^a^The structure of product **7a** was confirmed by ^1^Н, ^13^С, and 2D (HMBC) NMR as well as HRMS.

As can be seen in Table 1, entry 8, the most efficient conditions for the reaction of **2a** and **3a** were found to be the use of ethanol as solvent as well as equimolar amounts of amine species and acetic acid under reflux. At the same time, in the absence of AcOH as additive, only traces of the desired product **7a** could be detected using either solvent. In addition, extending the reaction time to over 4 h under reflux led to a significant decrease in yield of the target material. Thus, optimal conditions could be established for this reaction, allowing the synthesis of enaminones **7** in moderate yields (Table 2). Note, that this is the first time for such a transformation to be reported.

**Table 2:** Synthesis of dihydropyrrolone derivatives **7**.^a^

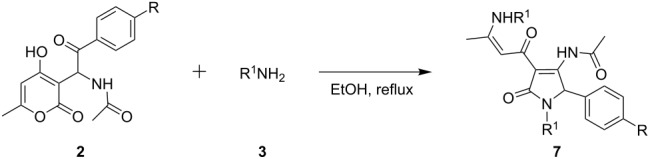				

Entry	R^1^	R	Product	Yield, %

1	4-CH_3_O–C_6_H_4_–(CH_2_)_2_	Cl	**7a**	60
2	H	Cl	**7b**	41
3^b^	H	Br	**7c**	42
4^b^	H	CH_3_	**7d**	30
5	CH_3_	Cl	**7e**	59
6	HO–(CH_2_)_2_	Cl	**7f**	57

^a^Reaction conditions: **2** (3 mmol), amine **3** (9 mmol), AcOH (9 mmol), EtOH, reflux, 4 h. ^b^Ammonium acetate (6 mmol) was used instead of amine **3** (9 mmol) and AcOH (9 mmol).

**Scheme 3:**
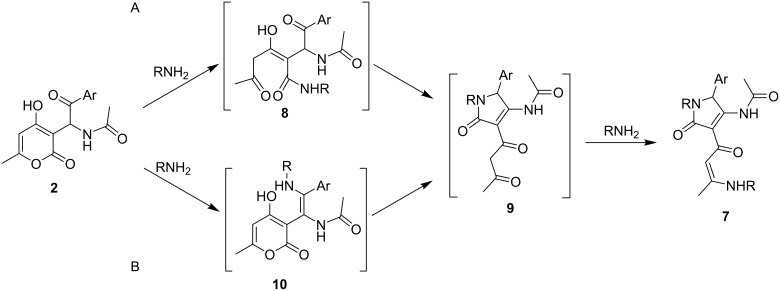
Possible reaction pathways for the formation of enaminone **7**.

There are two possible reaction pathways for the investigated conversion. The first possibility includes the attack of an amine, and pyranone ring-opening to afford the acyclic intermediate **8** (Scheme 3A). Then, the latter undergoes cyclization to intermediate diketone **9**. The alternative approach includes an initial condensation of amine and aryl ketone to give the enamine intermediate **10**, which can subsequently undergo opening of the pyranone ring, yielding dihydropyrrolone **9** (Scheme 3B). Following this, the diketone-containing substituent of **9** reacts with a second equivalent of amine, forming the final enaminone **7**. After this general synthetic method for enaminone targets had been developed, the obtained compounds were further employed in the synthesis of pyrrolopyridinones **1**.

The presence of the two key reactive groups in adjacent positions in **7**, the acetamide moiety and the enaminoketone functionality, should allow for an intramolecular condensation and the formation of the target pyrrolopyridinones **1**. To investigate this, first, enaminone **7a** was chosen as a model compound in a test reaction in acidic medium in order to identify the most efficient conditions for the transformation to **1a** (Table 3). Several organic and inorganic acids and mixtures thereof were tested for this conversion. As it was established, the choice of acid was crucial. As such, the best yield was obtained after 1 h under reflux in AcOH/HCl, 1:1, v/v (Table 3, entry 3). All other attempts, i.e., applying longer reaction times and using different acids/acid mixtures, only provided inferior results.

**Table 3:** Optimization of the reaction conditions towards pyrrolo[3,4-*b*]pyridin-5-one derivative **1a** from **7a**.

Entry	Reagent/solvent, v/v	*t*, h	Yield, %

1	AcOH	1	0
2	HCl	1	traces
3	HCl/AcOH, 1:1	1	82
4	HCl/AcOH, 1:1	4	35
5	TsOH/HCl, 1:20	1	traces
6	TsOH/AcOH, 1:20	1	41
7	TsOH/AcOH, 1:20	4	14
8	H_2_SO_4_/AcOH, 1:1	1	0
9	H_2_SO_4_/AcOH, 1:5	1	27
10	H_2_SO_4_/AcOH, 1:20	1	44
11	H_2_SO_4_	1	0

After having established a novel synthetic pathway to the target pyrrolopyridinone **1a**, we decided to test the sequential transformation in a much more convenient, one-pot procedure. For this purpose, after completion of the first step, condensation of acetamide **2a** and the corresponding amine **3a**, the solvent was evaporated to give the crude enaminone **7a** as a brown solid. The obtained residue was then heated in an AcOH/HCl mixture to give the target pyrrolopyridinone **1a**. In addition, the described one-pot synthesis, besides from being more straightforward, provided better yields as compared to the standard two-step procedure due to circumventing the isolation of the intermediate enaminones **7**. Applying this method to the whole range of substrates **2** and **3** allowed to access the target materials **1** in high yields (Table 4).

**Table 4:** One-pot method for the synthesis of pyrrolo[3,4-*b*]pyridin-5-one derivatives **1**.^a^

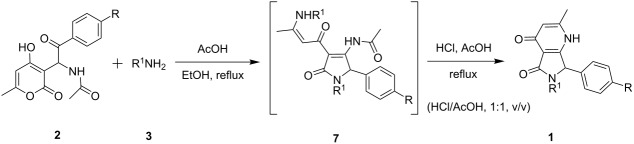				

Entry	R^1^	R	Product	Yield, %

1	4-CH_3_O–C_6_H_4_(CH_2_)_2_	Cl	**1a**	60
2	H	Cl	**1b**	50
3	H	Br	**1c**	60
4	H	CH_3_	**1d**	59
5	CH_3_	Cl	**1e**	59
6	CH_3_	Br	**1f**	70
7	CH_3_	F	**1g**	54
8	CH_3_	CH_3_O	**1h**	55
9	CH_3_	CH_3_	**1i**	68
10	HO–(CH_2_)_2_	Cl	**1j**	50

^a^Reaction conditions: 1) **2** (3 mmol), amine **3** (9 mmol), AcOH (9 mmol), EtOH, reflux, 4 h. 2) HCl/AcOH, 5 mL/5 mL, reflux, 1 h.

**Scheme 4:**
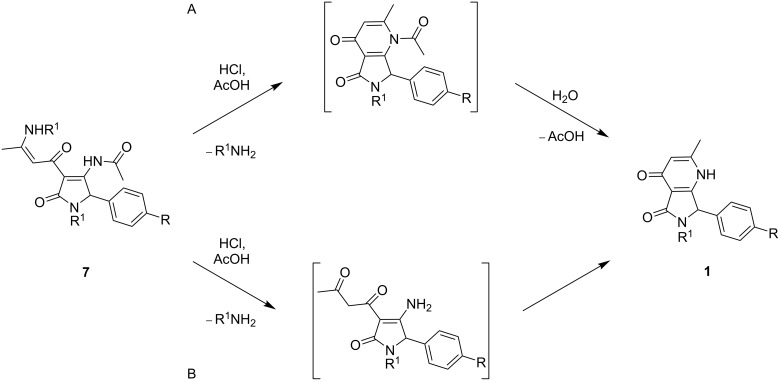
Possible reaction pathways for the formation of pyrrolo[3,4-*b*]pyridin-5-one derivatives **1**.

Again, the transformation of **7** to **1** may have proceeded via one of two possible routes: The two-step process might start with an intramolecular condensation of the enaminone moiety in **7**, followed by acidic hydrolysis of the acetyl group, furnishing the desired pyrrolopyridinones **1** (Scheme 4A). Alternatively, these steps could be reversed in order, i.e., the hydrolysis of both enaminoketone and acetamide fragments proceeds first, followed by intramolecular cyclization (Scheme 4B).

**Figure 1:**
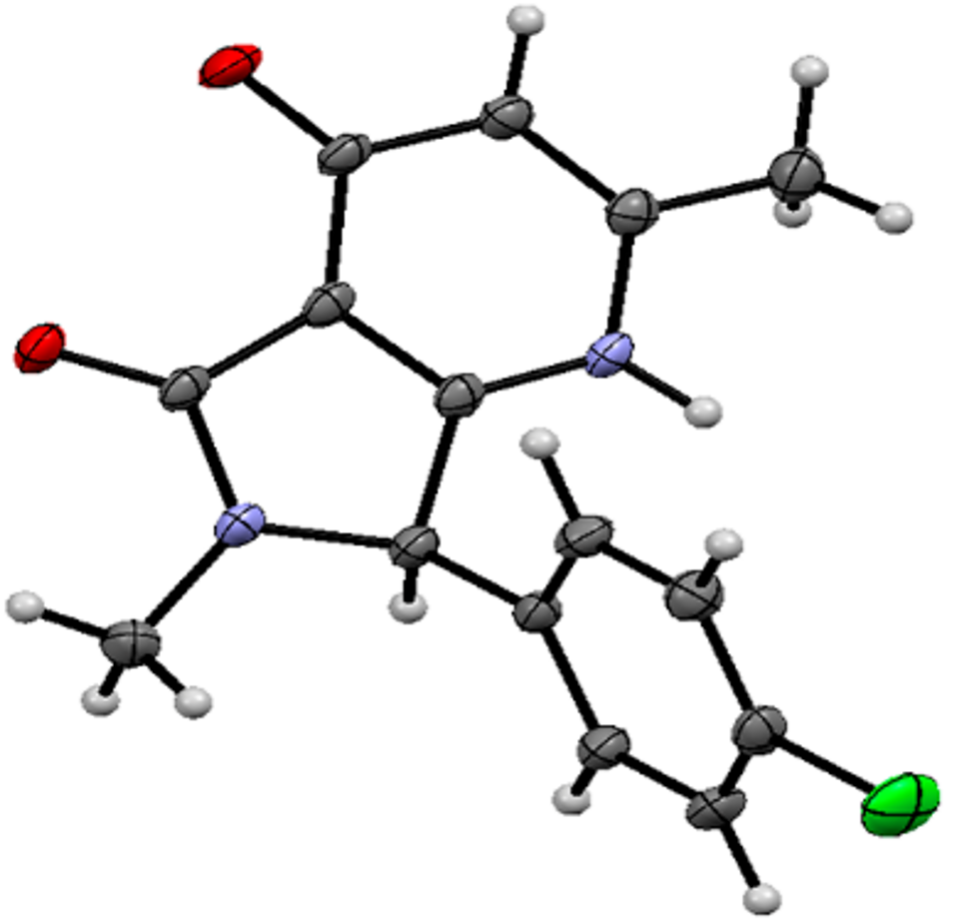
View of the structure of **1e** in the crystal (CCDC: 1921613). Thermal ellipsoids indicate 50% probability. Solvent molecules have been omitted for clarity.

**Scheme 5:**
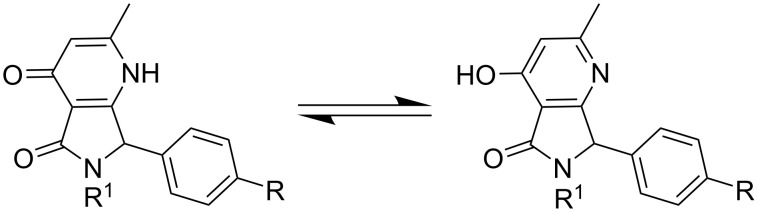
Tautomeric equilibrium of pyrrolo[3,4-*b*]pyridin-5-one derivatives **1** in solution.

The structure of the 4-chloro-substituted pyrrolopyrimidinone **1e** was proven by single crystal X-ray analysis (Figure 1). Despite the unambiguous dominance of the pyridone-based conformation in the solid state of **1e**, two tautomers of **1** might exist in solution, with the other form being a hydroxypyridine (Scheme 5). This assumption is supported by an apparent broadening of several signals in the ^1^H NMR spectra of **1**, as shown in Supporting Information File 1.

## Conclusion

In conclusion, a new, general synthetic pathway to various substituted pyrrolopyridinones **1** was developed. The reaction of *N*-(1-(4-hydroxy-6-methyl-2-oxo-2*H*-pyran-3-yl)-2-oxo-2-arylethyl)acetamides with a diverse family of amines was performed in two consecutive steps, without isolation of the intermediate enaminones **7**. The described one-pot method combines efficiency and simplicity, granting a convenient access to the pyrrolo[3,4-*b*]pyridin-5-one scaffold. In addition, single crystal X-ray crystallographic analysis unequivocally confirmed the structure assigned to the obtained products.

## Supporting Information

File 1 Experimental procedures, characterization data of all products, copies of ^1^H, ^13^C, 2D NMR, and HRMS spectra of all compounds, and X-ray data for compound **1e**.
