# Supplementary material for: One-pot synthesis of substituted pyrrolo[3,4-b]pyridine-4,5-diones based on the reaction of N-(1-(4-hydroxy-6-methyl-2-oxo-2H-pyran-3-yl)-2-oxo-2-arylethyl)acetamide with amines
Source: Beilstein J Org Chem. 2019 Nov 25;15:2840–6. doi: 10.3762/bjoc.15.277 (PMC6902885; doi:10.3762/bjoc.15.277)
Supplement: File 1 — Experimental procedures, characterization data of all products, copies of 1H, 13C, 2D NMR, and HRMS spectra of all compounds, and X-ray data for compound 1e. [file Beilstein_J_Org_Chem-15-2840-s001.pdf]

BEILSTEIN JOURNAL OF ORGANIC CHEMISTRY

Supporting Information

for

One-pot synthesis of substituted pyrrolo[3,4-*b*]pyridine-4,5-diones based on the reaction of *N*-(1-(4-hydroxy-6-methyl-2-oxo-2*H*-pyran-3-yl)-2-oxo-2-arylethyl)acetamide with amines

Valeriya G. Melekhina, Andrey N. Komogortsev, Boris V. Lichitsky, Vitaly S. Mityanov, Artem N. Fakhrutdinov, Arkady A. Dudinov, Vasily A. Migulin, Yulia V. Nelyubina, Elizaveta K. Melnikova and Michail M. Krayushkin

Beilstein J. Org. Chem. **2019,** *15,* 2840–2846. doi:10.3762/bjoc.15.277

Experimental procedures, characterization data of all products, copies of ^{1}H, ^{13}C, 2D NMR, and HRMS spectra of all compounds, and X-ray data for compound 1e

License and Terms: This is a supporting information file under the terms of the Creative Commons Attribution License (http://creativecommons.org/licenses/by/4.0). Please note that the reuse, redistribution and reproduction in particular requires that the authors and source are credited.
The license is subject to the *Beilstein Journal of Organic Chemistry* terms and conditions: (https://www.beilstein-journals.org/bjoc)

Table of contents

1. General information S2
2. General procedure for the synthesis of all compounds S2
3. Characterization data of all products S3
4. Copies of ^{1}H and ^{13}C NMR spectra for all compounds S11
5. Copies of HRMS for all compounds S32
6. 2D NMR spectra for compounds **7e** and **1e** S44
7. X-Ray analysis of compounds **1e** S46

1. General information

Unless otherwise stated, all starting chemicals were commercially available and used as received. All reactions were performed without inert gas atmosphere. NMR spectra were recorded with Bruker AM 300 (300 MHz), Bruker AV 400 (400 MHz), Bruker DRX 500 (500 MHz), and Bruker AV 600 (600 MHz) spectrometers in DMSO-d_6. Chemical shifts (ppm) are given relative to solvent signals (2.50 ppm for ^{1}H NMR and 39.52 ppm for ^{13}C NMR). High-resolution mass spectra (HRMS) were obtained on a Bruker micrOTOF II instrument using electrospray ionization (ESI). IR spectra were registered on a Bruker ALPHA spectrophotometer as KBr pellets. The melting points were determined on a Kofler hot stage. Crystallographic data for the structure reported in this paper have been deposited in the Cambridge Crystallographic Data Centre as CCDC 1921613.

2. General procedure for the synthesis of compounds 2, 7 and 1.

*Experimental procedure for the preparation of compounds **2.***

A mixture of 4-hydroxy-6-methyl-2*H*-pyran-2-one (**4**, 3 mmol), the corresponding arylglyoxal **5** (3 mmol) and acetamide (**6**, 9 mmol) in MeCN was refluxed for 6 h. The reaction mixture was cooled to room temperature and the precipitate formed was collected by filtration and washed with MeCN (3 × 5 mL).

*Experimental procedure for the preparation of compounds **7**.*

A mixture of the corresponding acetamide derivatives **2** (3 mmol), amines **3** (9 mmol) and AcOH (9 mmol) in EtOH was refluxed for 4 h. The reaction mixture was cooled to room temperature and the precipitate formed was collected by filtration and washed with EtOH (3 × 5 mL). In the case of using ammonium acetate as the source of ammonia, we used 6 equivalents of this substance.

*Experimental one-pot procedure for the preparation of pyrrolo[3,4-b]pyridin-5-one derivatives **1**.*

A mixture of the corresponding acetamide derivatives **2** (3 mmol), amines **3** (9 mmol) and AcOH (9 mmol) in EtOH was refluxed for 4 h. The solvent was removed under reduced pressure. Then, a mixture of hydrochloric acid and acetic acid (5 mL:5 mL) was added to the residue and the mixture refluxed for 1 h. After completion of the reaction, the solvent was evaporated under reduced pressure and water (10 mL) and NaOAc (0.5 g) were added and left overnight. The obtained solid product was filtered and recrystallized from 50% EtOH.

3. **Characterization data of all products**

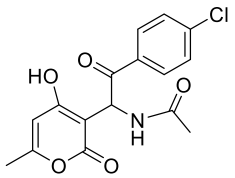

N-(2-(4-Chlorophenyl)-1-(4-hydroxy-6-methyl-2-oxo-2*H*-pyran-3-yl)-2-oxoethyl)acetamide (**2a**)

White powder; yield 42%; mp 249-250°C; ^{1}H NMR (300 MHz, DMSO-d_6) δ 12.25 (s, 1H), 8.13 (d, J = 7.3 Hz, 1H), 7.74 (d, J = 8.5 Hz, 2H), 7.52 (d, J = 8.5 Hz, 2H), 6.23 (d, J = 7.6 Hz, 1H), 6.00 (s, 1H), 2.12 (s, 3H), 1.91 (s, 3H). ^{13}C NMR (75 MHz, DMSO-d_6) δ 193.91, 169.38, 167.40, 163.18, 162.90, 137.64, 134.20, 129.11, 128.67, 99.85, 98.40, 50.51, 22.31, 19.37. IR spectrum, ν, cm^{-1} : 3300, 3105, 3043, 2959, 2931, 2874, 2860, 2628, 2368, 1691, 1591, 1561, 1448, 1422, 1398, 1275, 1233, 1171, 1140, 1117, 1095, 1045, 997. HRMS: Calculated for $C_{16}H_{14}ClNO_5$ $[M+H]^+$: 336.0633. Found:336.0636.

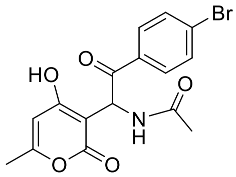

N-(2-(4-Bromophenyl)-1-(4-hydroxy-6-methyl-2-oxo-2*H*-pyran-3-yl)-2-oxoethyl)acetamide (**2b**)

White powder; yield 45%; mp 251-252°C; ^{1}H NMR (300 MHz, DMSO-d_6) δ 12.24 (s, 1H), 8.13 (d, J = 7.6 Hz, 1H), 7.66 (s, 4H), 6.22 (d, J = 7.6 Hz, 1H), 6.00 (s, 1H), 2.12 (s, 3H), 1.91 (s, 3H). ^{13}C NMR (75 MHz, DMSO-d_6) δ 194.09, 169.34, 167.35, 163.14, 162.87, 134.52, 131.57, 129.19, 126.70, 99.81, 98.35, 50.44, 22.28, 19.33. IR spectrum, ν, cm^{-1} : 3368, 3297, 3097, 2959, 2933, 2874, 2860, 1712, 1693, 1618, 1590, 1561, 1528, 1448, 1422, 1395, 1275, 1234, 1173, 1139, 1120, 1072, 1042, 996. HRMS: Calculated for $C_{16}H_{14}BrNO_5$ $[M+H]^+$: 380.0128. Found: 380.0127.

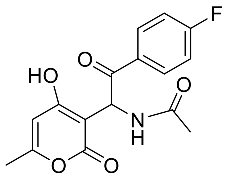

N-(2-(4-Fluorophenyl)-1-(4-hydroxy-6-methyl-2-oxo-2*H*-pyran-3-yl)-2-oxoethyl)acetamide (**2c**)

White powder; yield 46%; mp 246-247°C; ^{1}H NMR (300 MHz, DMSO-d_6) δ 12.21 (s, 1H), 8.11 (d, J = 7.7 Hz, 1H), 7.82 (dd, J_F = 8.5, 5.5 Hz, 2H), 7.29 (t, J_F = 8.8 Hz, 2H), 6.24 (d, J = 7.7 Hz, 1H), 6.01 (s, 1H), 2.13 (s, 3H), 1.91 (s, 3H). ^{13}C NMR (75 MHz, DMSO-d_6) δ 193.36, 169.33, 167.31, 166.31, 163.07 (d, J_{CF} = 14 Hz), 162.82, 132.09, 130.10 (d, J_{CF} = 9.4 Hz), 115.57 (d, J_{CF} = 22.0 Hz), 99.83, 98.50, 50.42, 22.30, 19.33. IR spectrum, ν, cm^{-1} : 3301, 3087, 2959, 2922, 2875, 2629, 2457, 2345, 1898, 1691, 1599, 1561, 1508, 1448, 1411, 1396, 1302, 1276, 1233, 1159, 1140, 1117, 1106, 1047, 998. HRMS: Calculated for $C_{16}H_{14}FNO_5$ $[M+H]^+$: 320.0929. Found: 320.0932.

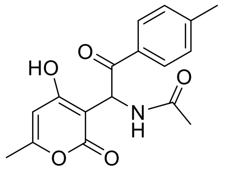

N-(1-(4-Hydroxy-6-methyl-2-oxo-2*H*-pyran-3-yl)-2-oxo-2-(p-tolyl)ethyl)acetamide (**2d**)

White powder; yield 46%; mp 241-242°C; ^{1}H NMR (300 MHz, DMSO-d_6) δ 12.14 (s, 1H), 8.05 (d, *J* = 7.7 Hz, 1H), 7.66 (d, *J* = 8.1 Hz, 2H), 7.24 (d, *J* = 8.0 Hz, 2H), 6.25 (d, *J* = 7.8 Hz, 1H), 5.99 (s, 1H), 2.32 (s, 3H), 2.11 (s, 3H), 1.91 (s, 3H). ^{13}C NMR (75 MHz, DMSO-d_6) δ 194.21, 169.27, 167.11, 163.16, 162.61, 143.11, 132.84, 128.99, 127.38, 99.84, 98.89, 50.33, 22.34, 21.06, 19.31. IR spectrum, ν, cm^{-1} : 3284, 3093, 3042, 2957, 2918, 2629, 2363, 2344, 1685, 1610, 1579, 1559, 1448, 1419, 1276, 1236, 1186, 1173, 1139, 1114, 1046, 997. HRMS: Calculated for $C_{17}H_{17}NO_5$ [M+H]$^+$: 316.1179. Found: 316.1184.

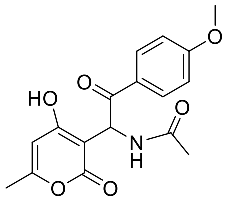

N-(1-(4-Hydroxy-6-methyl-2-oxo-2*H*-pyran-3-yl)-2-(4-methoxyphenyl)-2-oxoethyl)acetamide (**2e**)

White powder; yield 47%; mp 238-239°C; ^{1}H NMR (300 MHz, DMSO-d_6) δ 12.10 (s, 1H), 8.02 (d, *J* = 7.9 Hz, 1H), 7.75 (d, *J* = 8.7 Hz, 2H), 6.97 (d, *J* = 8.8 Hz, 2H), 6.23 (d, *J* = 7.8 Hz, 1H), 6.00 (s, 1H), 3.80 (s, 3H), 2.12 (s, 3H), 1.90 (s, 3H). ^{13}C NMR (75 MHz, DMSO-d_6) δ 192.98, 169.26, 167.11, 163.19, 162.83, 162.60, 129.58, 128.02, 113.74, 99.90, 99.06, 55.45, 50.18, 22.38, 19.34. IR spectrum, ν, cm^{-1} : 3442, 3288, 2959, 2927, 2873, 2858, 1679, 1604, 1578, 1560, 1509, 1449, 1417, 1276, 1260, 1240, 1180, 1139, 1116, 1075, 1033, 998. HRMS: Calculated for $C_{17}H_{17}NO_6$ [M+H]$^+$: 332.1129. Found: 332.1128.

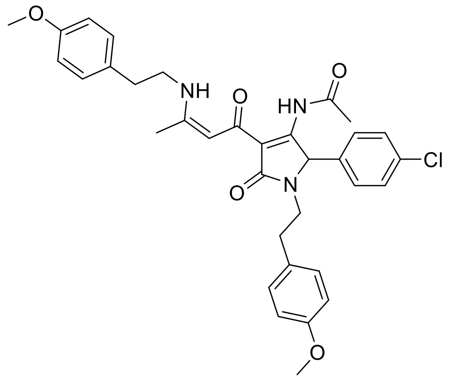

N-(2-(4-Chlorophenyl)-1-(4-methoxyphenethyl)-4-(3-((4-methoxyphenethyl)amino)but-2-enoyl)-5-oxo-2,5-dihydro-1*H*-pyrrol-3-yl)acetamide (**7a**)

White powder; yield 60%; mp 165-166°C; ^{1}H NMR (300 MHz, DMSO-*d*$_6$) δ 12.14 (s, 1H), 11.16 (s, 1H), 7.50 – 7.31 (m, 2H), 7.31 – 7.16 (m, 2H), 7.16 – 7.08 (m, 2H), 7.07 – 6.94 (m, 2H), 6.94 – 6.85 (m, 2H), 6.85 – 6.74 (m, 2H), 6.35 (s, 1H), 5.71 (s, 1H), 3.85 – 3.60 (m, 6H), 3.59 – 3.48 (m, 2H), 2.88 – 2.75 (m, 2H), 2.74 – 2.57 (m, 2H), 2.53 – 2.31 (m, 2H), 1.99 (s, 6H). ^{13}C NMR (75 MHz, DMSO-*d*$_6$) δ 181.74, 167.25, 166.39, 166.17, 159.17, 157.92, 157.76, 134.71, 132.62, 130.70, 130.35, 130.00, 129.88, 129.75, 129.40, 128.52, 113.85, 113.82, 108.60, 94.35, 61.73, 54.97, 44.68, 40.89, 34.91, 32.95, 24.06, 19.01. IR spectrum, ν, cm^{-1} : 3449, 3102, 3060, 3007, 2966, 2939, 2908, 2837, 1717, 1673, 1612, 1571, 1513, 1491, 1440, 1414, 1370, 1296, 1280, 1247, 1184, 1089, 1032, 994. HRMS: Calculated for $C_{34}H_{36}ClN_3O_5$ [M+H]$^+$: 602.2416. Found: 602.2414.

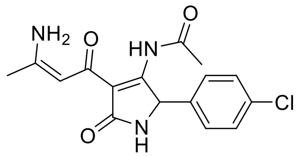

N-(4-(3-Aminobut-2-enoyl)-2-(4-chlorophenyl)-5-oxo-2,5-dihydro-1*H*-pyrrol-3-yl)acetamide (**7b**)

White powder; yield 41%; mp 281-282°C; ^{1}H NMR (300 MHz, DMSO-*d*$_6$) δ 12.21 (s, 1H), 9.90 (s, 1H), 8.29 (s, 1H), 8.07 (s, 1H), 7.37 (d, *J* = 7.6 Hz, 2H), 7.16 (d, *J* = 7.8 Hz, 2H), 6.30 (s, 1H), 5.82 (s, 1H), 2.00 (s, 3H), 1.98 (s, 3H). ^{13}C NMR (101 MHz, DMSO-*d*$_6$) δ 183.45, 169.01, 167.51, 165.73, 161.35, 137.16, 132.12, 129.43, 128.36, 108.88, 93.62, 58.00, 24.17, 21.98. IR spectrum, ν, cm^{-1} : 3650, 3630, 3458, 3307, 3272, 1735, 1700, 1654, 163, 1625, 1596, 1561, 1542, 1508, 1491, 1438, 1421, 1375, 1363, 1301, 1226, 1194, 1129, 1087, 1040, 1015, 993. HRMS: Calculated for $C_{16}H_{16}ClN_3O_3$ [M+H]$^+$: 334.0953. Found: 334.0953.

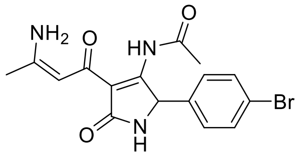

N-(4-(3-Aminobut-2-enoyl)-2-(4-bromophenyl)-5-oxo-2,5-dihydro-1*H*-pyrrol-3-yl)acetamide (**7c**)

White powder; yield 42%; mp 290-291°C; ^{1}H NMR (300 MHz, DMSO-d_6) δ 12.21 (s, 1H), 9.89 (s, 1H), 8.30 (s, 1H), 8.07 (s, 1H), 7.50 (d, *J* = 8.4 Hz, 2H), 7.10 (d, *J* = 8.4 Hz, 2H), 6.30 (s, 1H), 5.80 (s, 1H), 2.00 (s, 3H), 1.8 (s, 3H). ^{13}C NMR (126 MHz, DMSO-d_6) δ 183.43, 169.00, 167.51, 165.74, 161.27, 137.59, 131.28, 129.77, 120.66, 108.87, 93.60, 58.07, 24.18, 21.98. IR spectrum, ν, cm^{-1} : 3630, 3442, 2923, 1735, 1702, 1655, 1637, 1625, 1561, 1490, 1458, 1438, 1375, 1293, 1220, 1193, 1179, 1128, 1107, 1073, 1035, 1012. HRMS: Calculated for $C_{16}H_{16}BrN_3O_3$ [M+H]$^+$: 378.0448. Found: 378.0442.

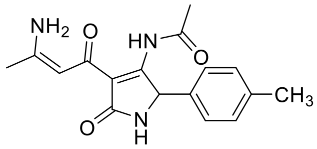

N-(4-(3-Aminobut-2-enoyl)-5-oxo-2-(*p*-tolyl)-2,5-dihydro-1*H*-pyrrol-3-yl)acetamide (**7d**)

White powder; yield 30%; mp 260-261°C; ^{1}H NMR (300 MHz, DMSO-d_6) δ 12.17 (s, 1H), 9.88 (s, 1H), 8.21 (s, 1H), 8.03 (s, 1H), 7.10 (d, *J* = 7.9 Hz, 2H), 7.02 (d, *J* = 8.0 Hz, 2H), 6.31 (s, 1H), 5.77 (s, 1H), 2.26 (s, 3H), 1.98 (s, 3H), 1.98 (s, 3H). ^{13}C NMR (75 MHz, DMSO-d_6) δ 183.66, 169.05, 167.36, 165.55, 161.97, 136.82, 135.09, 128.87, 127.38, 109.53, 108.81, 93.62, 58.38, 24.19, 21.97, 20.68. IR spectrum, ν, cm^{-1} : 3179, 3073, 1700, 1655, 1626, 1577, 1560, 1438, 1430, 1357, 1307, 1229, 1193, 1182, 1128, 1113, 994, 913. HRMS: Calculated for $C_{17}H_{19}N_3O_3$[M+H]$^+$: 314.1499. Found: 314.1506.

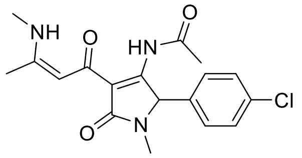

N-(2-(4-Chlorophenyl)-1-methyl-4-(3-(methylamino)but-2-enoyl)-5-oxo-2,5-dihydro-1*H*-pyrrol-3-yl)acetamide (**7e**)

White powder; yield 59%; mp 210-211°C; ^{1}H NMR (300 MHz, DMSO-d_6) δ 12.18 (s, 1H), 10.98 (s, 1H), 7.40 (d, *J* = 8.4 Hz, 2H), 7.16 (d, *J* = 8.4 Hz, 2H), 6.39 (s, 1H), 5.82 (s, 1H), 2.99 (d, *J* = 5.2 Hz, 3H), 2.54 (s, 3H), 2.05 (s, 3H), 1.99 (s, 3H). ^{13}C NMR (101 MHz, DMSO-d_6) δ 181.60, 167.33, 166.51, 158.98, 134.83, 132.56, 129.89, 128.56, 108.78, 94.09, 63.03, 29.70, 26.04, 24.07, 21.39, 19.15. IR spectrum, ν, cm^{-1} : 1719, 1692, 1626, 1573, 1519, 1489, 1445, 1418, 1388, 1305, 1275, 1190, 1156, 1103, 1077, 1013, 991, 954. HRMS: Calculated for $C_{18}H_{20}ClN_3O_3$[M+H]$^+$: 362.1266. Found: 362.1266.

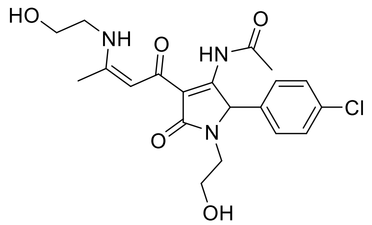

N-(2-(4-Chlorophenyl)-1-(2-hydroxyethyl)-4-(3-((2-hydroxyethyl)amino)but-2-enoyl)-5-oxo-2,5-dihydro-1*H*-pyrrol-3-yl)acetamide (**7f**)

White powder; yield 57%; mp 174-175°C; ^{1}H NMR (300 MHz, DMSO-d_6) δ 12.15 (s, 1H), 11.14 (s, 1H), 7.40 (d, J = 7.4 Hz, 2H), 7.15 (d, J = 7.8 Hz, 2H), 6.39 (s, 1H), 6.02 (s, 1H), 4.96 (s, 1H), 4.78 (s, 1H), 3.65-3.35 (m, 4H), 3.34-3.26 (m, 4H), 2.07 (s, 3H), 2.00 (s, 3H). ^{13}C NMR (126 MHz, DMSO-d_6) δ 181.79, 167.50, 166.66, 166.46, 159.54, 134.92, 132.63, 130.16, 128.57, 108.77, 94.44, 62.38, 60.12, 59.30, 45.57, 41.45, 24.20, 19.49. IR spectrum, ν, cm^{-1} : 3364, 2927, 1716, 1657, 1622, 1578, 1519, 1491, 1440, 1414, 1374, 1318, 1254, 1207, 1115, 1074, 1046, 999, 888. HRMS: Calculated for $C_{20}H_{24}ClN_3O_5$ [M+H]$^+$: 422.1477. Found: 422.1471.

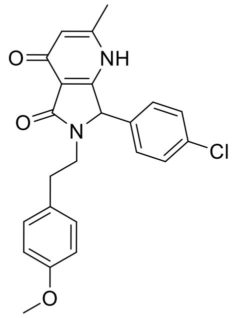

7-(4-Chlorophenyl)-6-(4-methoxyphenethyl)-2-methyl-6,7-dihydro-1*H*-pyrrolo[3,4-*b*]pyridine-4,5-dione (**1a**)

White powder; yield 60%; mp 251-252°C; ^{1}H NMR (300 MHz, DMSO-d_6) δ 7.44 (d, J = 7.8 Hz, 2H), 7.15 (d, J = 8.1 Hz, 2H), 7.04 (d, J = 7.9 Hz, 2H), 6.81 (d, J = 8.2 Hz, 2H), 6.45 (s, 1H), 5.37 (s, 1H), 3.95 – 3.77 (m, 1H), 3.70 (s, 3H), 2.85 – 2.67 (m, 2H), 2.68 – 2.55 (m, 1H), 2.26 (s, 3H). ^{13}C NMR (75 MHz, DMSO-d_6) δ 165.33, 157.77, 134.96, 133.17, 130.69, 129.87, 129.47, 129.24, 128.91, 128.35, 115.25, 113.84, 112.73, 110.63, 62.54, 54.95, 40.89, 32.89, 22.42. IR spectrum, ν, cm^{-1} : 3440, 3274, 2959, 2928, 1773, 1685, 1677, 1647, 1617, 1542, 1514, 1491, 1458, 1439, 1412, 1390, 1282, 1245, 1180, 1087, 1036, 1015, 833. HRMS: Calculated for $C_{23}H_{21}ClN_2O_3$ [M+H]$^+$: 409.1313. Found: 409.1311.

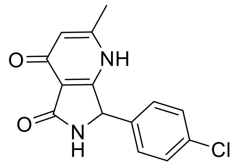

7-(4-Chlorophenyl)-2-methyl-6,7-dihydro-1*H*-pyrrolo[3,4-*b*]pyridine-4,5-dione (**1b**)

White powder; yield 50%; mp >300°C; ^{1}H NMR (300 MHz, DMSO-d_6) δ 8.64 (s, 1H), 7.42 (s, 2H), 7.32 (s, 2H), 6.45 (s, 1H), 5.53 (s, 1H), 2.28 (s, 3H). ^{13}C NMR (75 MHz, DMSO-d_6) δ 167.82, 137.22, 134.65, 132.51, 129.78, 129.10, 128.48, 128.01, 126.61, 110.73, 58.77, 24.40. IR spectrum, ν, cm^{-1}: 3137, 3089, 2959, 2922, 2361, 1694, 1647, 1614, 1541, 1492, 1448, 1338, 1248, 1215, 1180, 1091, 1017, 833. HRMS: Calculated for $C_{14}H_{11}ClN_2O_2$ [M+H]$^+$: 275.0582. Found: 275.0579.

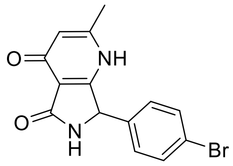

7-(4-Bromophenyl)-2-methyl-6,7-dihydro-1*H*-pyrrolo[3,4-*b*]pyridine-4,5-dione (**1c**)

White powder; yield 60%; mp 190-191°C; ^{1}H NMR (300 MHz, DMSO-d_6) δ 11.17 (s, 1H), 8.71 (s, 1H), 7.56 (d, J = 7.7 Hz, 2H), 7.24 (d, J = 8.2 Hz, 2H), 6.59 (s, 1H), 5.50 (s, 1H), 2.30 (s, 3H). ^{13}C NMR (151 MHz, DMSO-d_6) δ 167.76, 137.62, 131.60, 131.42, 129.80, 129.61, 129.54, 129.30, 121.17, 110.64, 58.69, 22.46. IR spectrum, ν, cm^{-1} : 3432, 3177, 3135, 3089, 3034, 2930, 2857, 1701, 1675, 1647, 1615, 1578, 1516, 1489, 1344, 1214, 1177, 1073, 1014, 859. HRMS: Calculated for $C_{14}H_{11}BrN_2O_2$ [M+H]$^+$: 319.0077. Found: 319.0072.

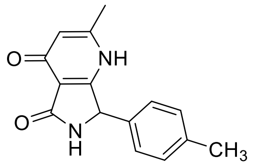

2-Methyl-7-(p-tolyl)-6,7-dihydro-1*H*-pyrrolo[3,4-*b*]pyridine-4,5-dione (**1d**)

White powder; yield 59%; mp >300°C; a mixture of isomers. ^{1}H NMR (400 MHz, DMSO-d_6) δ 8.73 (s, 1H), 8.45 (s, 1H), 7.62 (d, J = 7.8 Hz, 0.5H), 7.46-7.36 (m, 0.7H), 7.17 (d, J = 7.5 Hz, 1.5H), 7.13 (d, J = 7.3 Hz, 1.5H), 7.0 (d, J = 7.8 Hz, 0.3H), 6.86 (d, J = 7.8 Hz, 0.5H), 6.31 (s, 0.1H), 6.15 (s, 0.9H), 5.96 (s, 0.1H), 5.32 (s, 0.9H), 2.53 (s, 0.75H), 2.29 (s, 2.25H), 2.19 (s, 2.25 H), 2.15 (s, 0.75H). ^{13}C NMR (101 MHz, DMSO-d_6) δ 167.85, 160.06, 136.39, 135.21, 128.44, 127.59, 126.86, 126.67, 113.30, 110.28, 60.37, 23.27, 20.17. IR spectrum, ν, cm^{-1} : 3412, 3399, 3162, 2959, 2926, 1672, 1646, 1597, 1543, 1513, 1480, 1412, 1272, 1247, 1211, 1181, 1072, 855. HRMS: Calculated for $C_{15}H_{14}N_2O_2$ [M+H]$^+$: 255.1128. Found:225.1124.

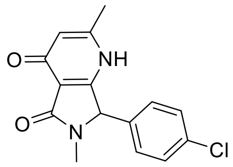

7-(4-Chlorophenyl)-2,6-dimethyl-6,7-dihydro-1*H*-pyrrolo[3,4-*b*]pyridine-4,5-dione (**1e**)

White powder; yield 59%; mp 270-271°C; ^{1}H NMR (300 MHz, DMSO-d_6) δ 7.43 (d, J = 8.0 Hz, 2H), 7.18 (d, J = 8.2 Hz, 2H), 6.29 (s, 1H), 5.37 (s, 1H), 2.72 (s, 3H), 2.19 (s, 3H). ^{13}C NMR (101 MHz, DMSO-d_6) δ 168.35, 167.38, 165.60, 158.42, 135.53, 132.88, 129.66, 128.86, 113.73, 110.72, 64.49, 26.49, 22.72. IR spectrum, ν, cm^{-1} : 3423, 3086, 3051, 2992, 2946, 2884, 2346, 1697, 1680, 1647, 1552, 1522, 1491, 1426, 1388, 1295, 1275, 1220, 1200, 1155, 1090, 1073, 1043, 1015, 948. HRMS: Calculated for $C_{15}H_{13}ClN_2O_2$ [M+H]$^+$: 289.0738. Found: 289.0740.

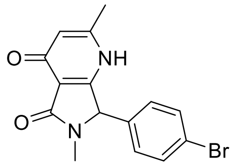

7-(4-Bromophenyl)-2,6-dimethyl-6,7-dihydro-1*H*-pyrrolo[3,4-*b*]pyridine-4,5-dione (**1f**)

White powder; yield 70%; mp 201-202°C; ^{1}H NMR (300 MHz, DMSO-d_6) δ 7.58 (d, J = 6.5 Hz, 2H), 7.13 (d, J = 4.5 Hz, 2H), 6.41 (s, 1H), 5.45 (s, 1H), 2.73 (s, 3H), 2.25 (s, 3H). ^{13}C NMR (75 MHz, DMSO-d_6) δ 166.12, 165.02, 137.79, 135.58, 131.88, 131.42, 130.02, 128.57, 121.63, 112.84, 64.39, 26.57, 23.01. IR spectrum, ν, cm^{-1} : 3492, 3437, 3253, 3109, 2980, 2889, 2830, 2702, 1682, 1643, 1614, 1548, 1511, 1488, 1374, 1267, 1218, 1195, 1146, 1071, 1041, 1012, 934. HRMS: Calculated for $C_{15}H_{13}BrN_2O_2$ [M+H]$^+$: 333.0233 Found: 333.0239.

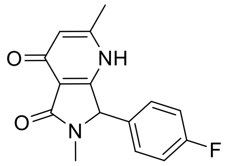

7-(4-Fluorophenyl)-2,6-dimethyl-6,7-dihydro-1*H*-pyrrolo[3,4-*b*]pyridine-4,5-dione (**1g**)

White powder; yield 54%; mp 230-231°C; ^{1}H NMR (300 MHz, DMSO-d_6) δ 7.29 – 7.11 (m, 4H), 6.37 (s, 1H), 5.44 (s, 1H), 2.72 (s, 3H), 2.24 (s, 3H). ^{13}C NMR (75 MHz, DMSO-d_6) δ 163.67, 163.58, 160.43, 160.35, 132.30, 129.91 (d, J = 8.5 Hz), 128.45 (d, J = 8.4 Hz) 115.81 (d, J = 21.6 Hz), 115.40, 115.11, 64.42, 26.51, 22.98. IR spectrum, ν, cm^{-1} : 3492, 3439, 3255, 3111, 2980, 2891, 2830, 2701, 2346, 1681, 1646, 1611, 1551, 1510, 1473, 1425, 1386, 1265, 1217, 1156, 1080, 1040, 1015, 977. HRMS: Calculated for $C_{15}H_{13}FN_2O_2$ [M+H]$^+$: 273.1034. Found: 273.1043

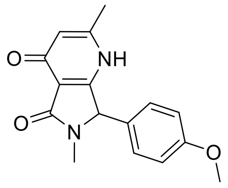

7-(4-Methoxyphenyl)-2,6-dimethyl-6,7-dihydro-1*H*-pyrrolo[3,4-*b*]pyridine-4,5-dione (**1h**)

White powder; yield 55%; mp 199-200°C; ^{1}H NMR (300 MHz, DMSO-d_6) δ 7.05 (s, 2H), 6.92 (d, 2H), 6.23 (s, 1H), 5.30 (s, 1H), 2.70 (s, 3H), 2.18 (s, 3H). ^{13}C NMR (151 MHz, DMSO-d_6) δ 168.75, 167.50, 165.66, 159.25, 129.03, 127.82, 114.25, 113.83, 113.69, 111.10, 64.66, 55.11, 26.33, 22.32. IR spectrum, ν, cm^{-1} : 3441, 1671, 1643, 1614, 1559, 1541, 1512, 1466, 1393, 1360,

1306, 1265, 1245, 1217, 1181, 1078, 1028, 978, 953. HRMS: Calculated for $C_{16}H_{16}ClN_2O_3$ $[M+H]^+$: 285.1234 Found: 285.1238.

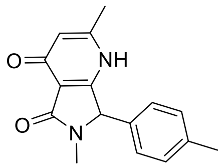

2,6-Dimethyl-7-(*p*-tolyl)-6,7-dihydro-1*H*-pyrrolo[3,4-*b*]pyridine-4,5-dione (**1i**)

White powder; yield 68%; mp 220-221°C; ^{1}H NMR (300 MHz, DMSO-d_6) δ 7.19 (d, *J* = 7.5 Hz, 2H), 7.05 (d, *J* = 7.8 Hz, 2H), 6.41 (s, 1H), 5.39 (s, 1H), 2.69 (s, 3H), 2.29 (s, 3H), 2.25 (s, 3H). ^{13}C NMR (75 MHz, DMSO-d_6) δ 165.50, 157.12, 137.87, 132.77, 129.49, 129.02, 127.75, 126.09, 113.08, 111.01, 64.40, 26.40, 21.98, 20.72. IR spectrum, ν, cm^{-1} : 3132, 3098, 2959, 2921, 2822, 1671, 1611, 1560, 1516, 1430, 1386, 1254, 1219, 1142, 1038, 851. HRMS: Calculated for $C_{16}H_{16}ClN_2O_2$ $[M+H]^+$: 269.1285. Found: 269.1294.

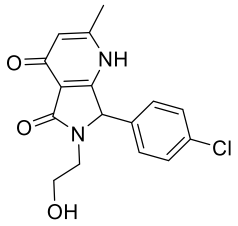

7-(4-Chlorophenyl)-6-(2-hydroxyethyl)-2-methyl-6,7-dihydro-1*H*-pyrrolo[3,4-*b*]pyridine-4,5-dione (**1j**)

White powder; yield 50%; mp 235-236°C; ^{1}H NMR (300 MHz, DMSO-d_6) δ 7.45 (d, *J* = 7.3 Hz, 2H), 7.20 (d, *J* = 7.4 Hz, 2H), 6.44 (s, 1H), 5.61 (s, 1H), 4.77 (s, 1H), 4.14 – 3.63 (m, 1H), 3.6-3.2 (m. 2H), 3.01-2.61 (m. 1H), 2.26 (s, 3H). ^{13}C NMR (126 MHz, DMSO-d_6) δ 170.24, 165.77, 135.24, 133.19, 133.08, 129.95, 129.90, 128.97, 128.92, 110.77, 61.71, 58.91, 41.79, 20.66. IR spectrum, ν, cm^{-1} : 3448, 3272, 3117, 2961, 2926, 2857, 1655, 1648, 1561, 1544, 1517, 1491, 1439, 1413, 1390, 1284, 1220, 1201, 1087, 1046, 1016, 845. HRMS: Calculated for $C_{16}H_{15}ClN_2O_3$ $[M+H]^+$: 319.0844. Found: 319.0842.

4. Copies of ^{1}H and ^{13}C NMR spectra for all compounds

^{1}H NMR spectrum (300 MHz) for **2a**

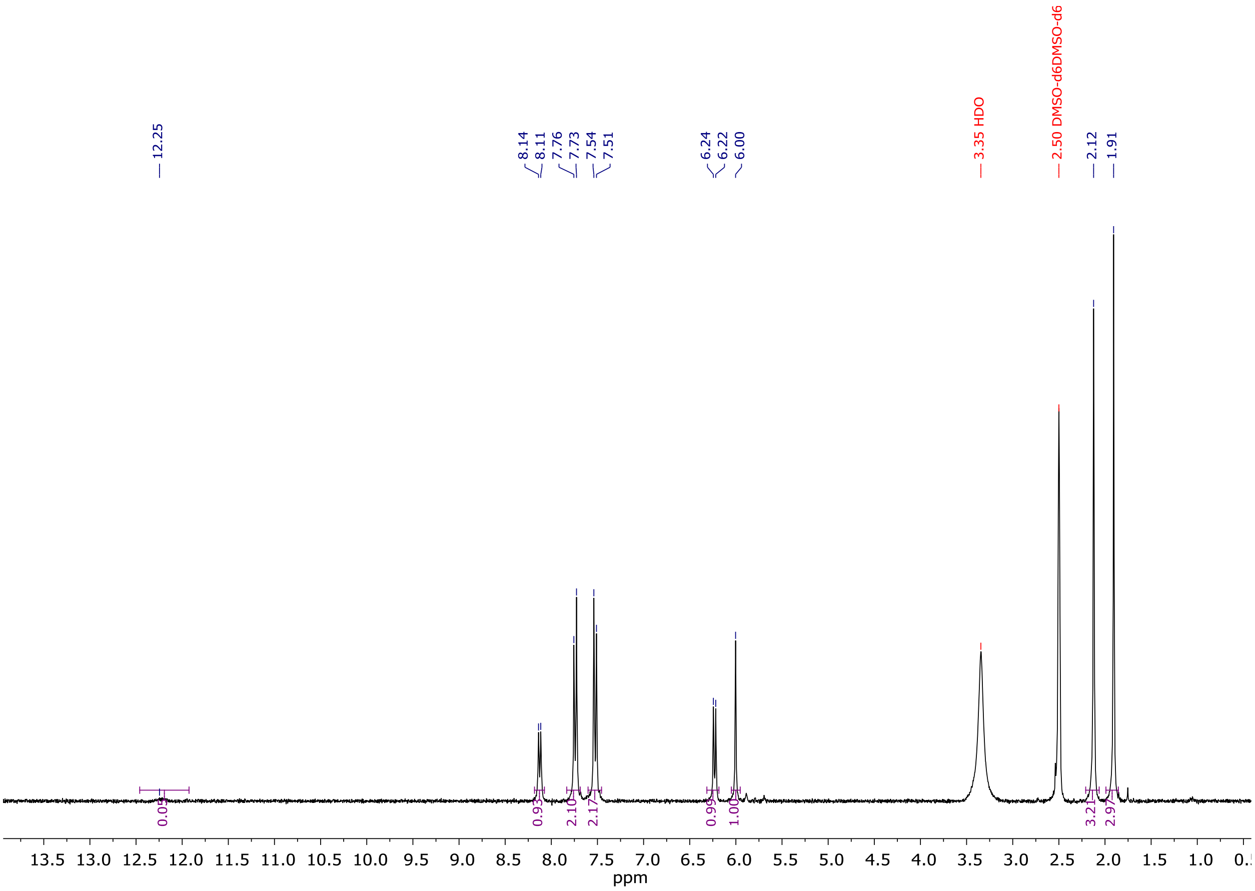

^{13}C NMR spectrum (75 MHz) for **2a**

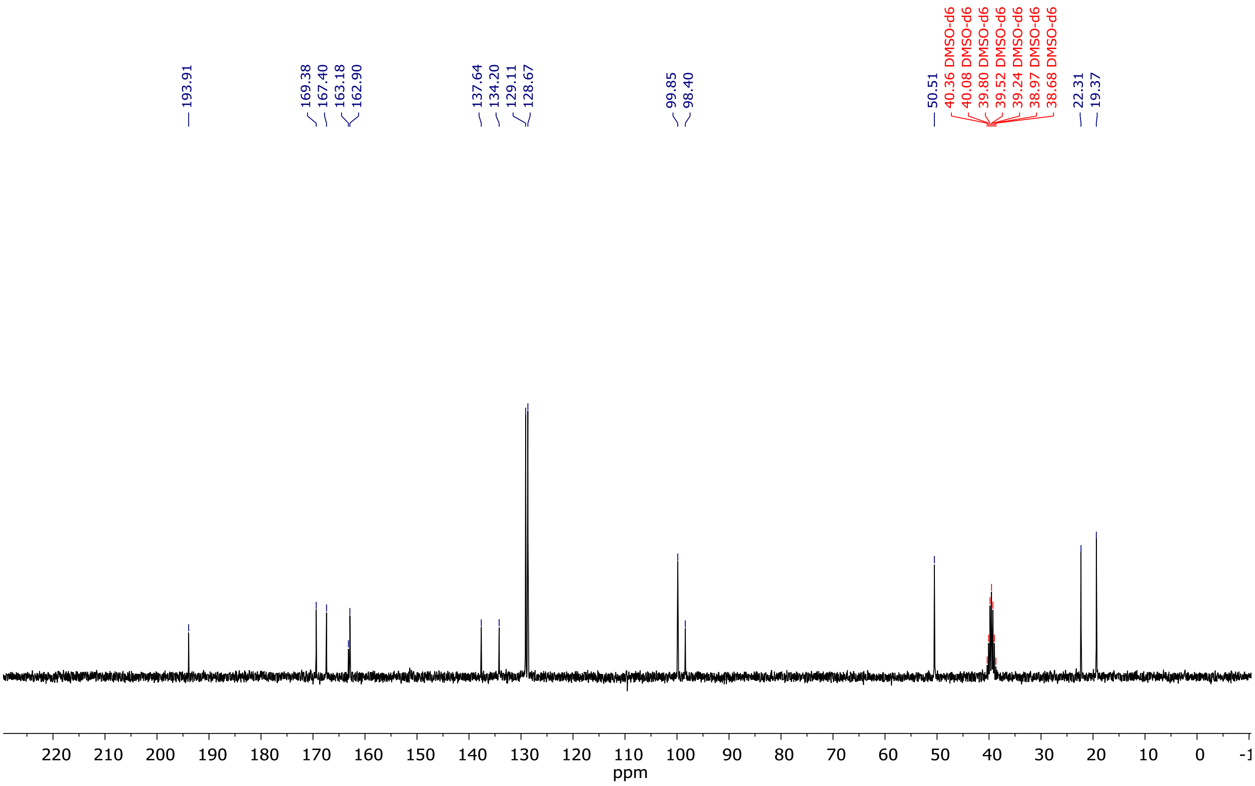

^{1}H NMR spectrum (300 MHz) for **2b**

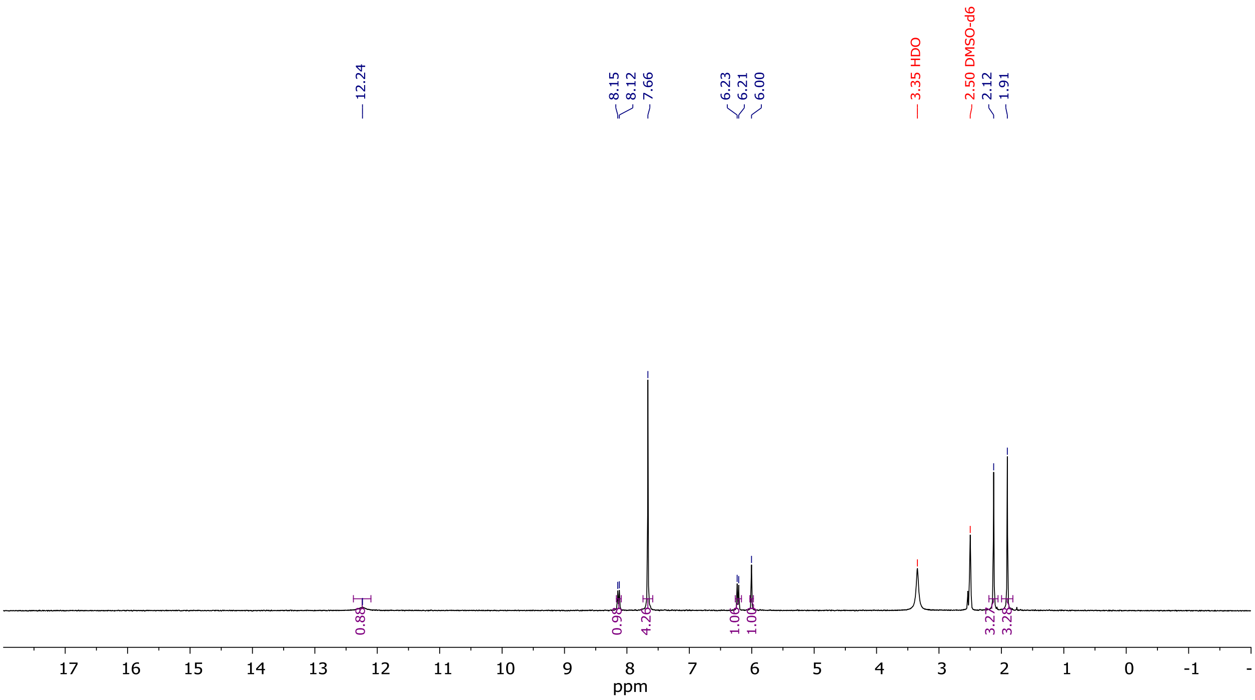

^{13}C NMR spectrum (75 MHz) for **2b**

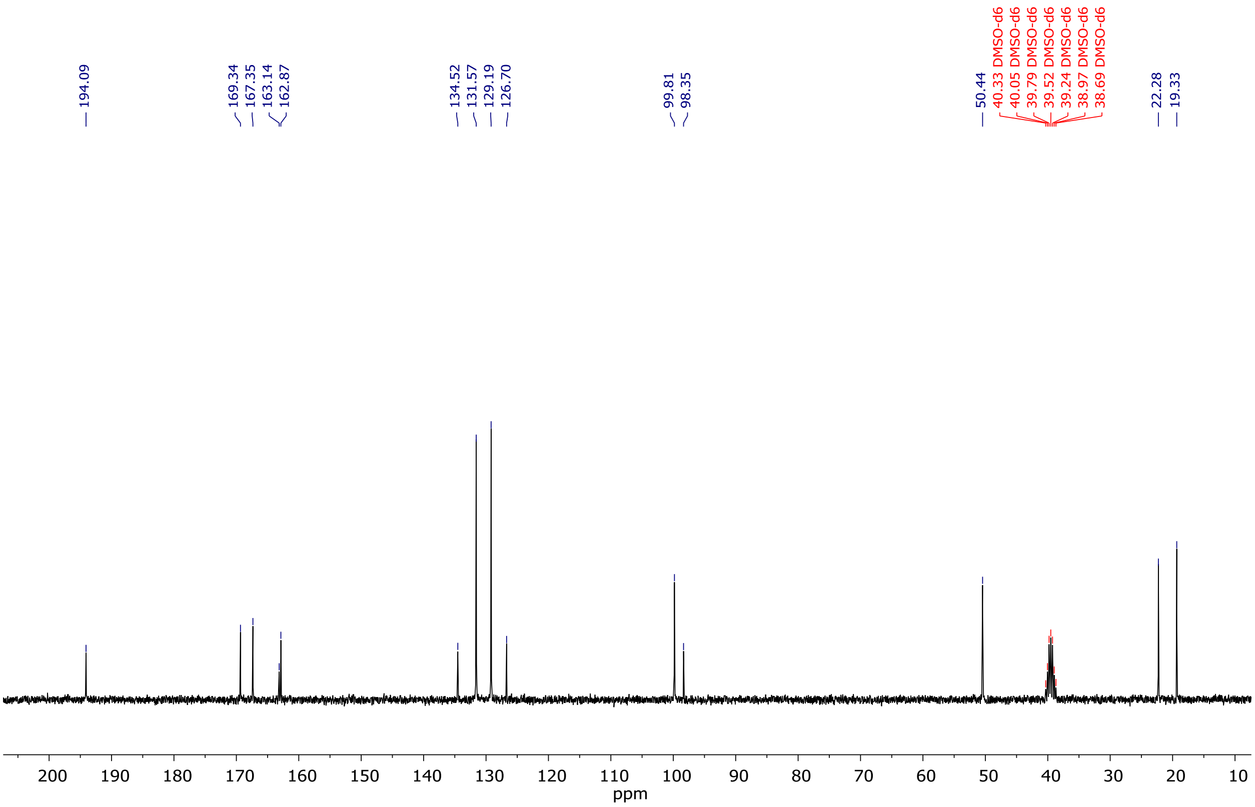

^{1}H NMR spectrum (300 MHz) for **2c**

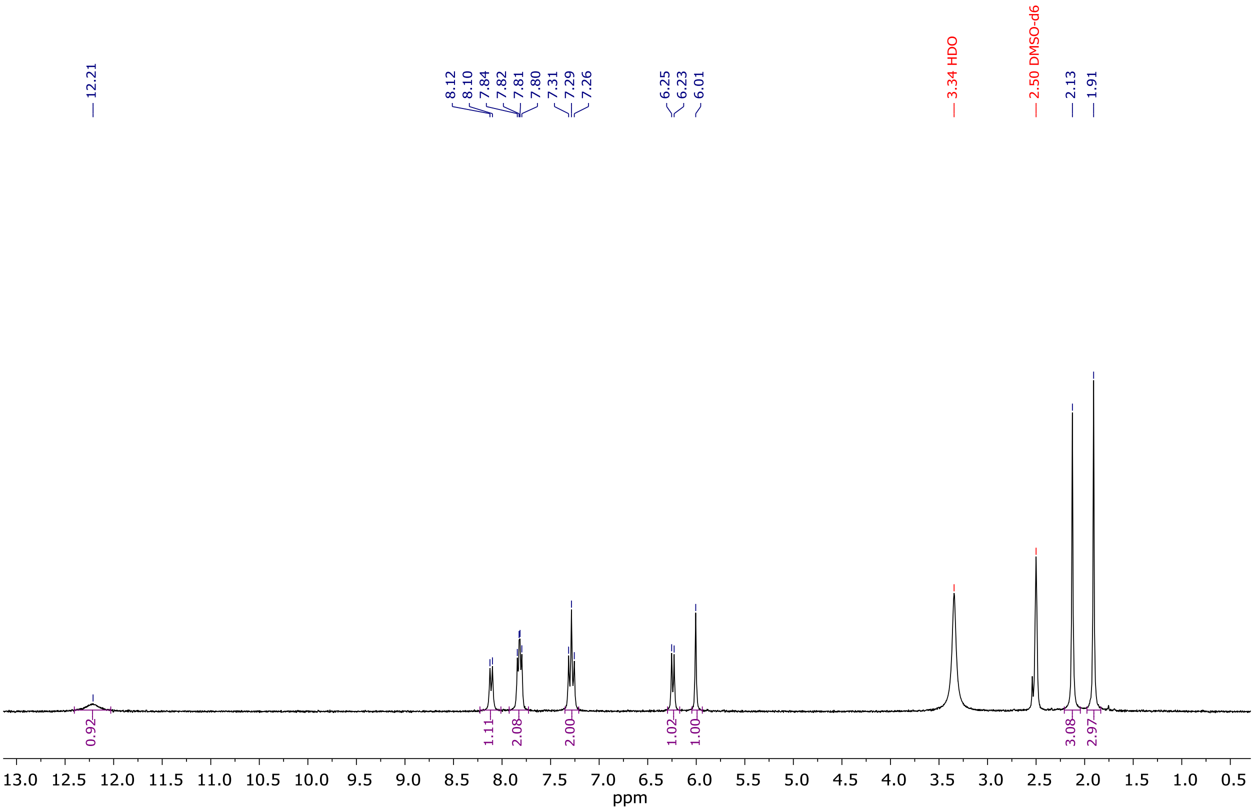

^{13}C NMR spectrum (75 MHz) for **2c**

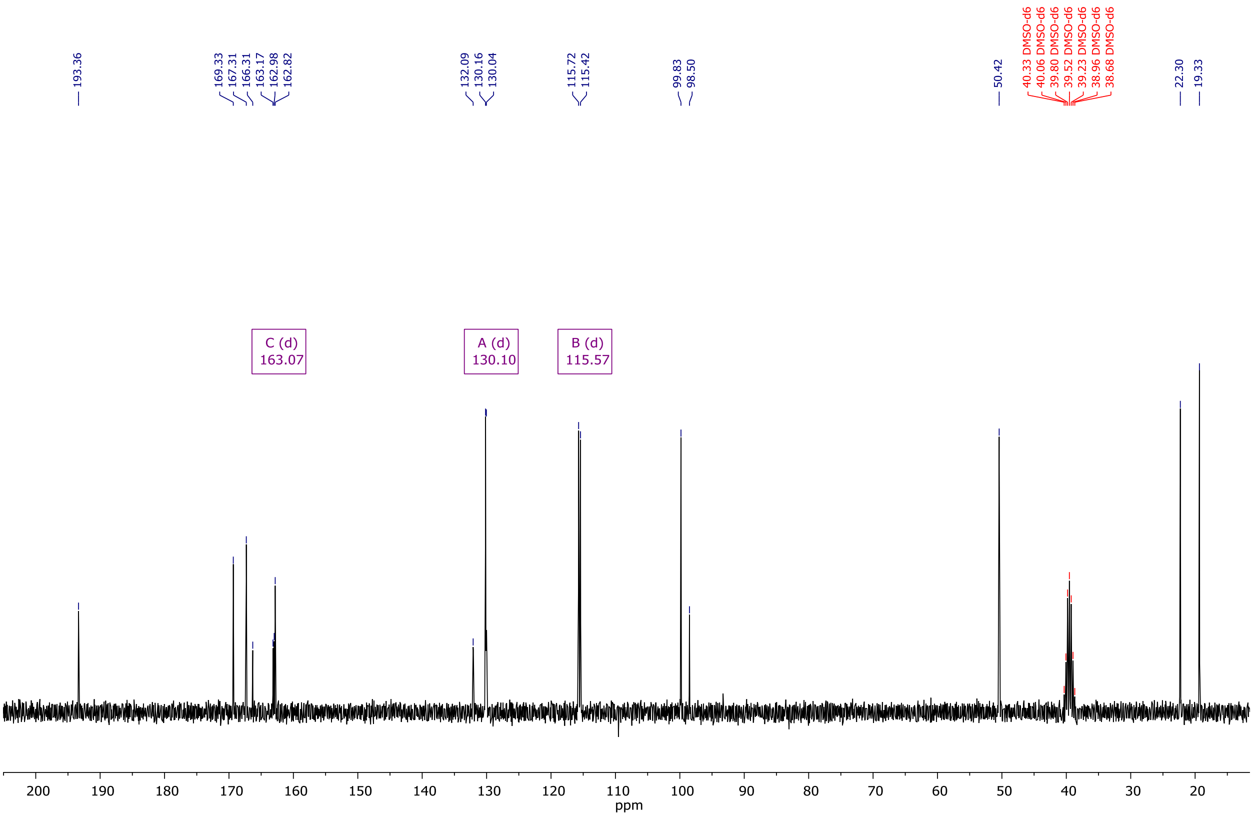

^{1}H NMR spectrum (300 MHz) for **2d**

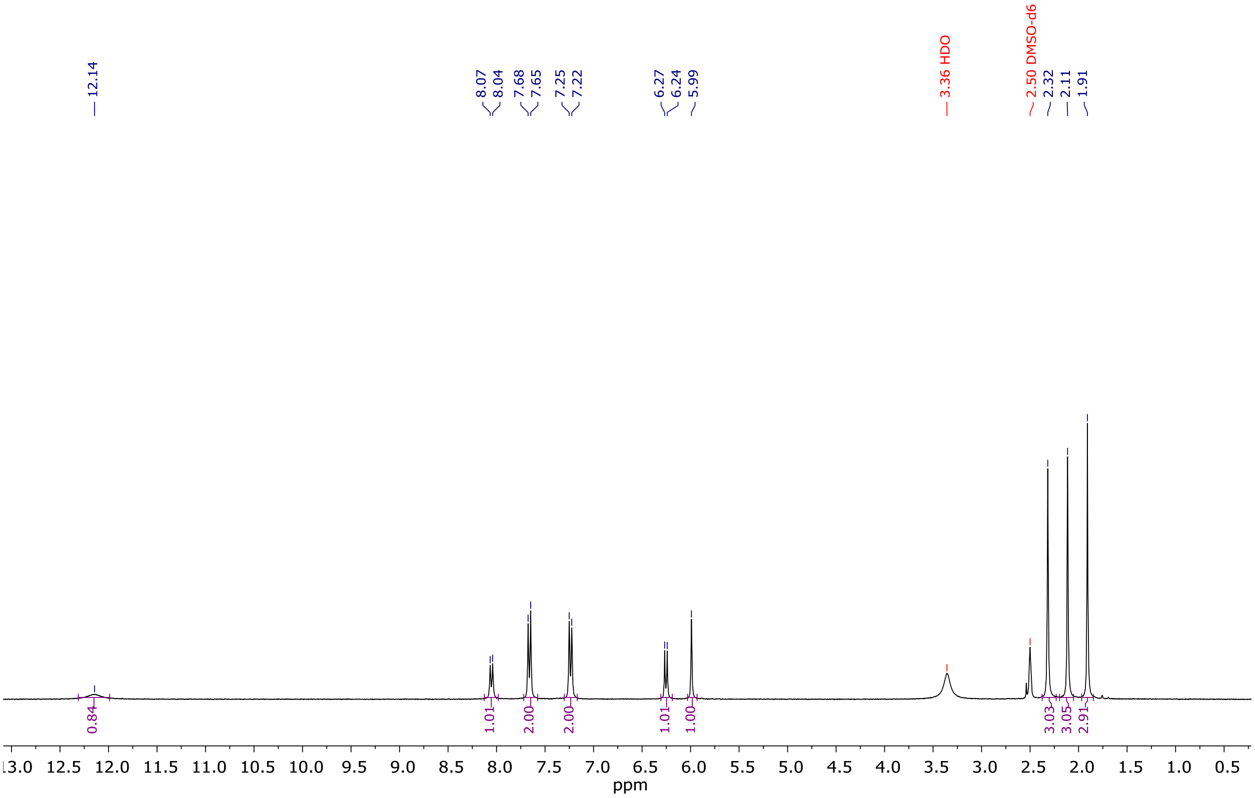

^{13}C NMR spectrum (75 MHz) for **2d**

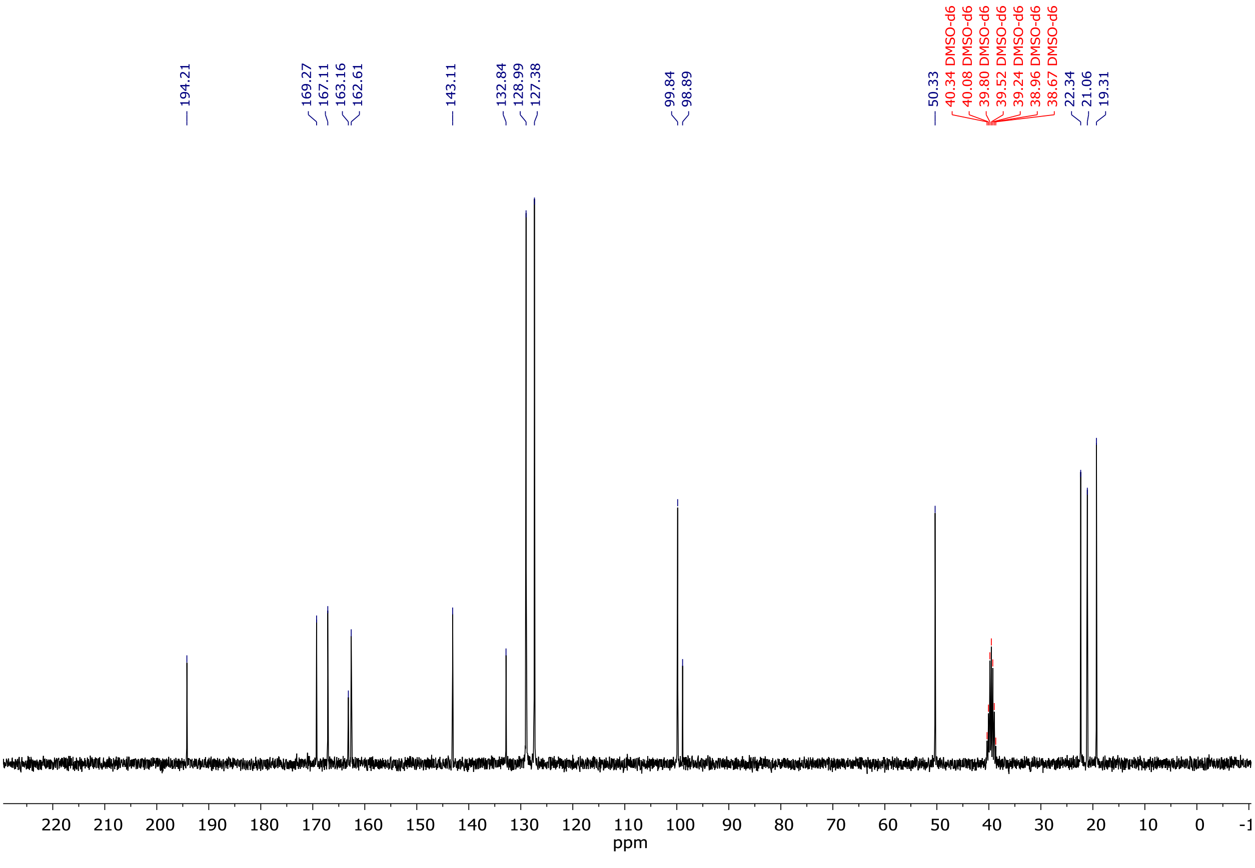

^{1}H NMR spectrum (300 MHz) for **2e**

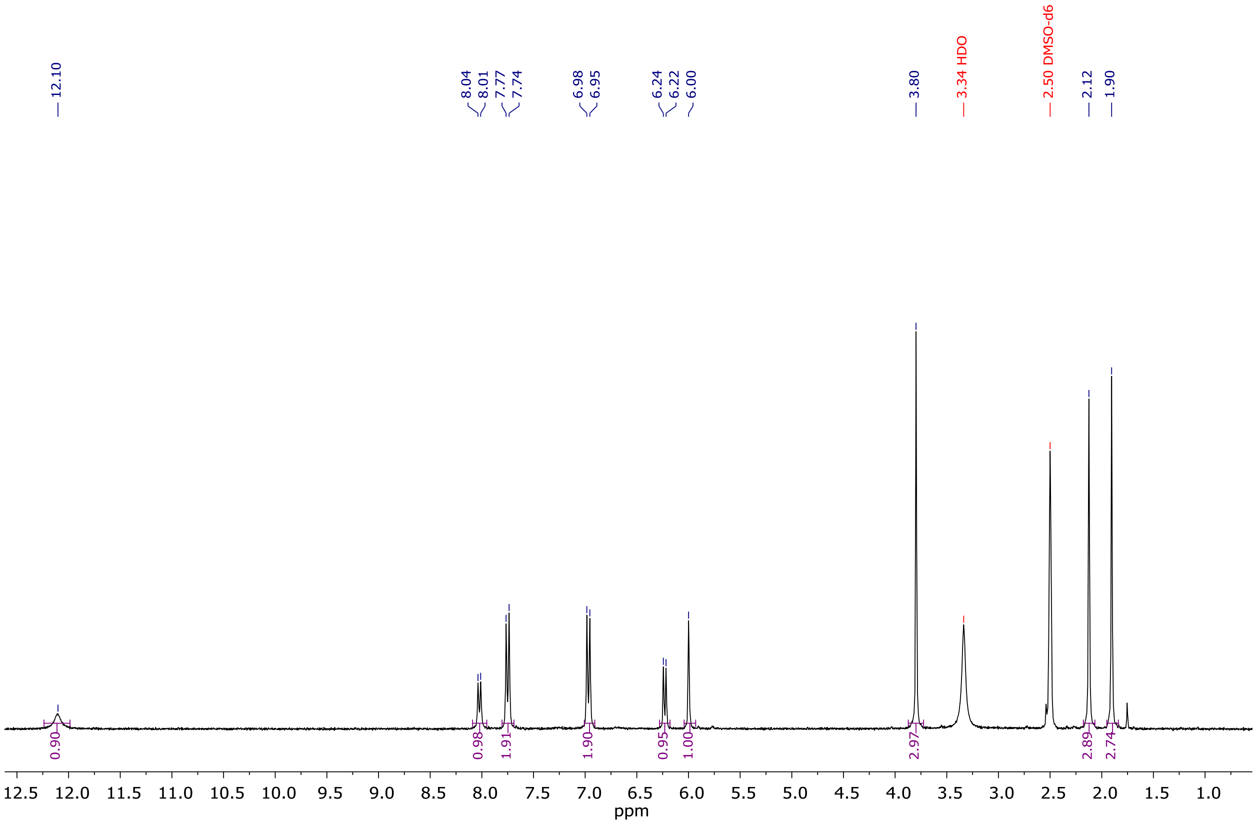

^{13}C NMR spectrum (75 MHz) for **2e**

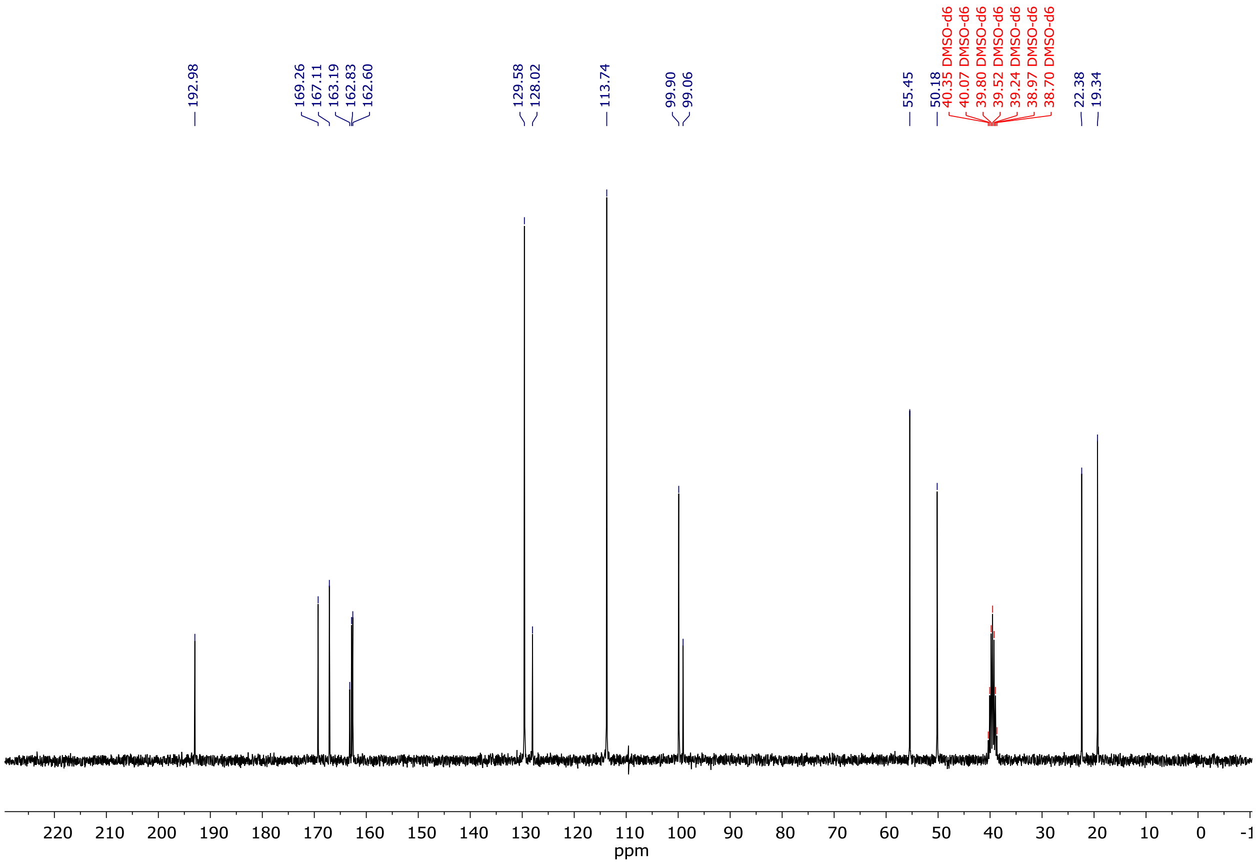

^{1}H NMR spectrum (300 MHz) for **7a**

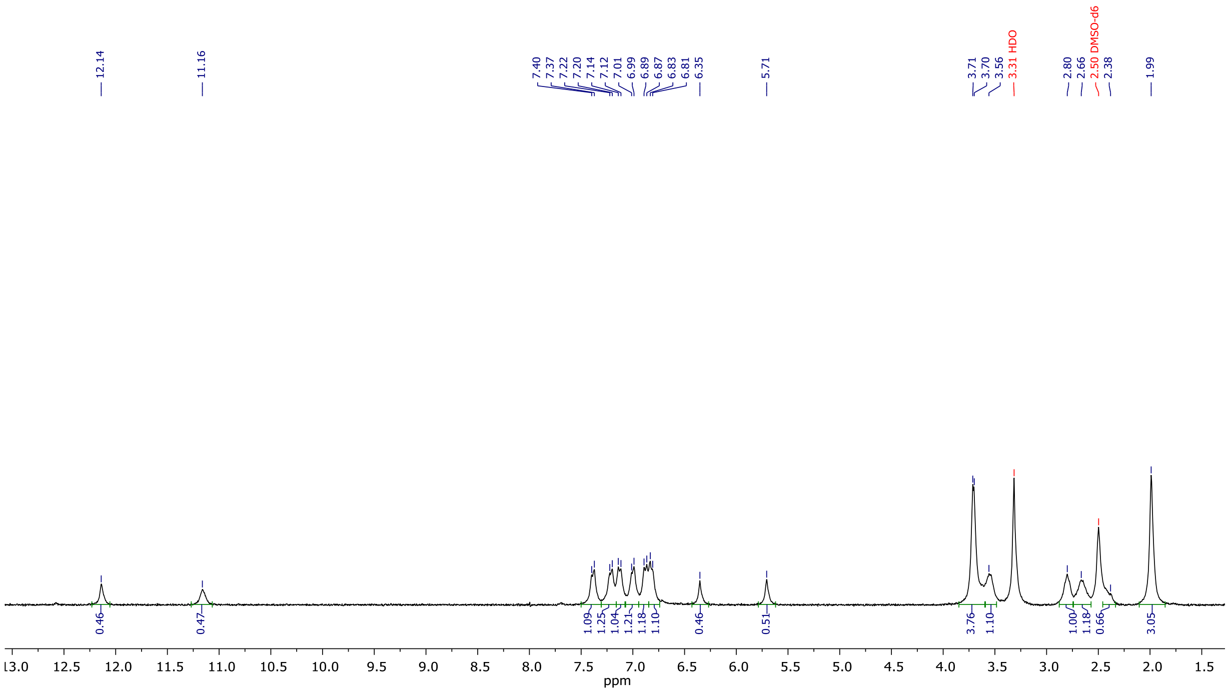

^{13}C NMR spectrum (75 MHz) for **7a**

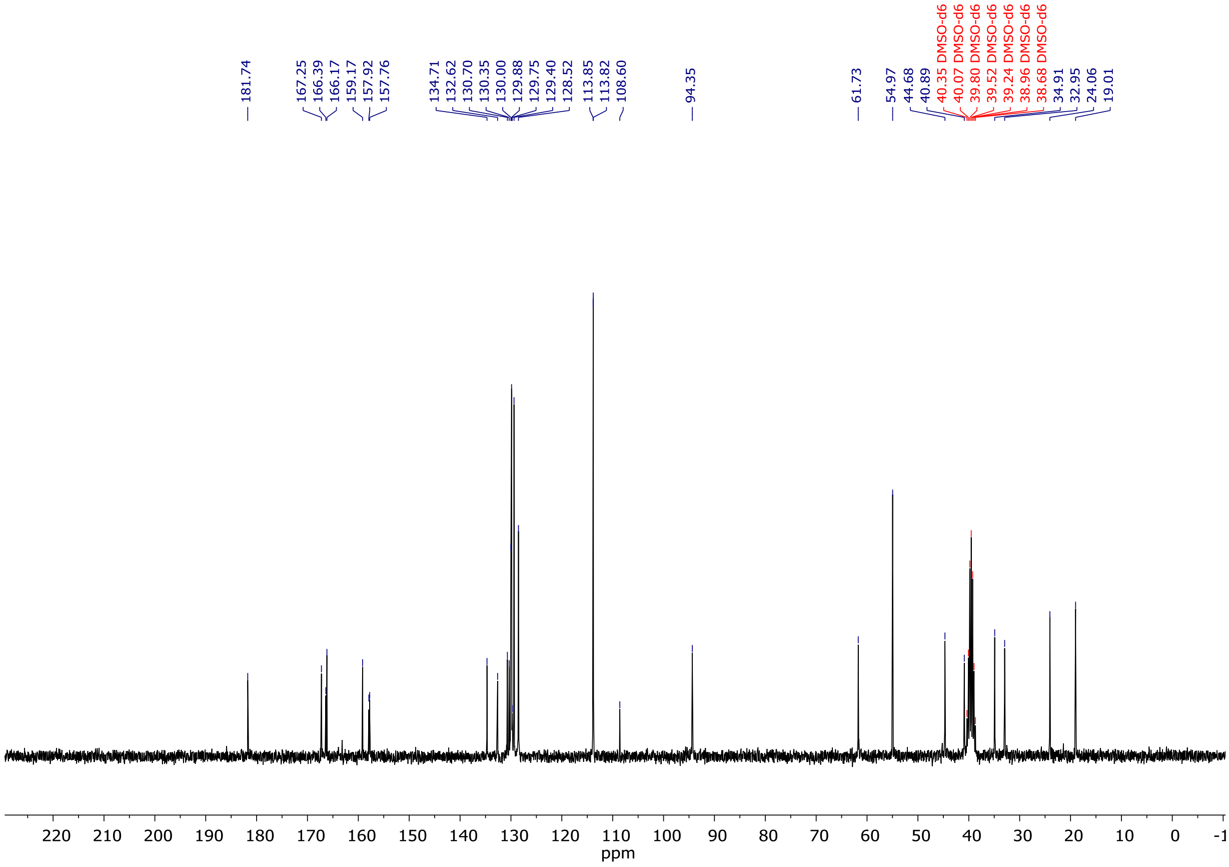

^{1}H NMR spectrum (300 MHz) for **7b**

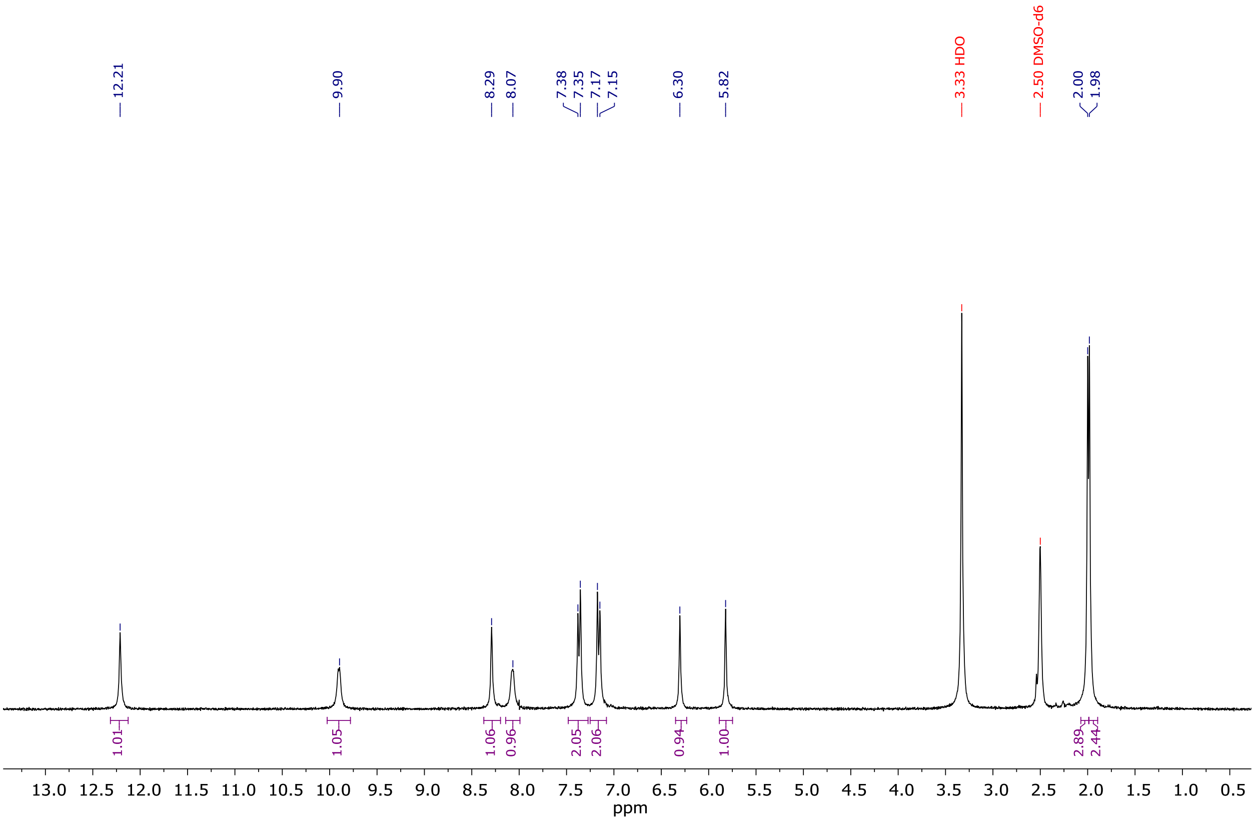

^{13}C NMR spectrum (101 MHz) for **7b**

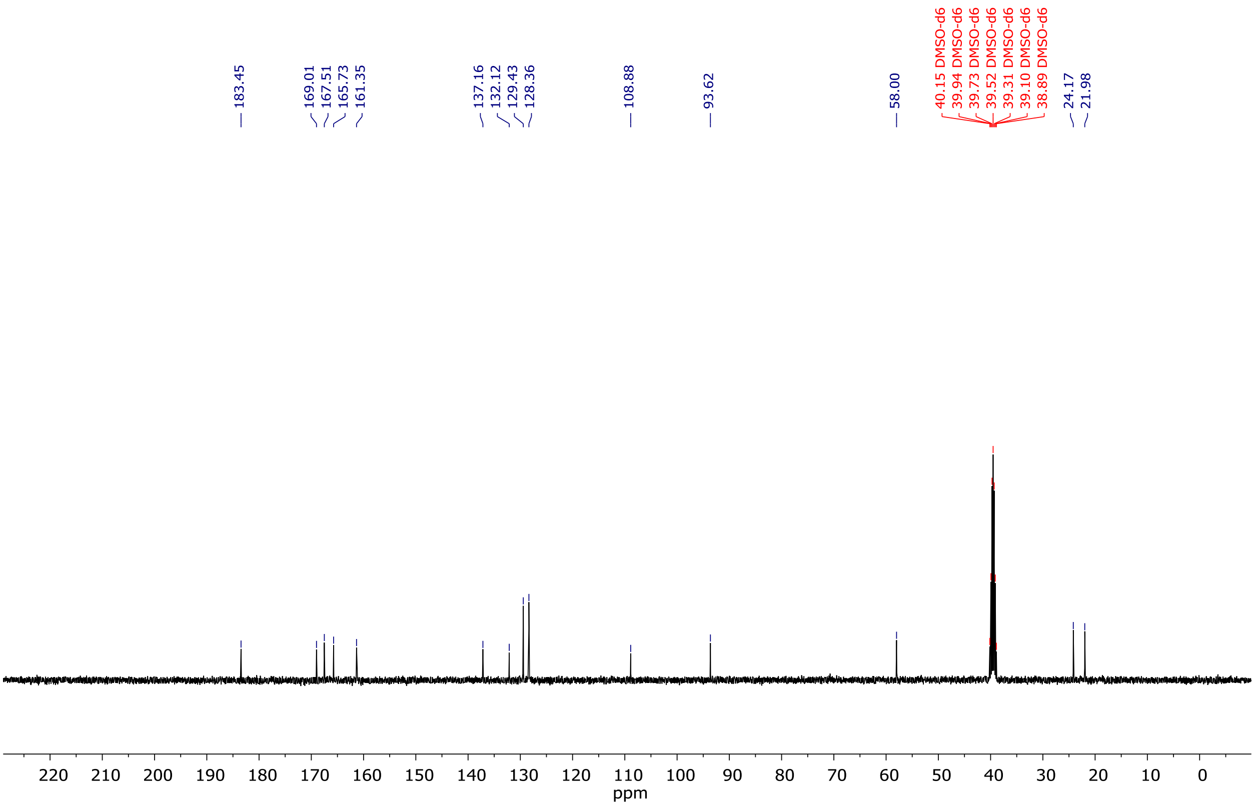

^{1}H NMR spectrum (300 MHz) for **7c**

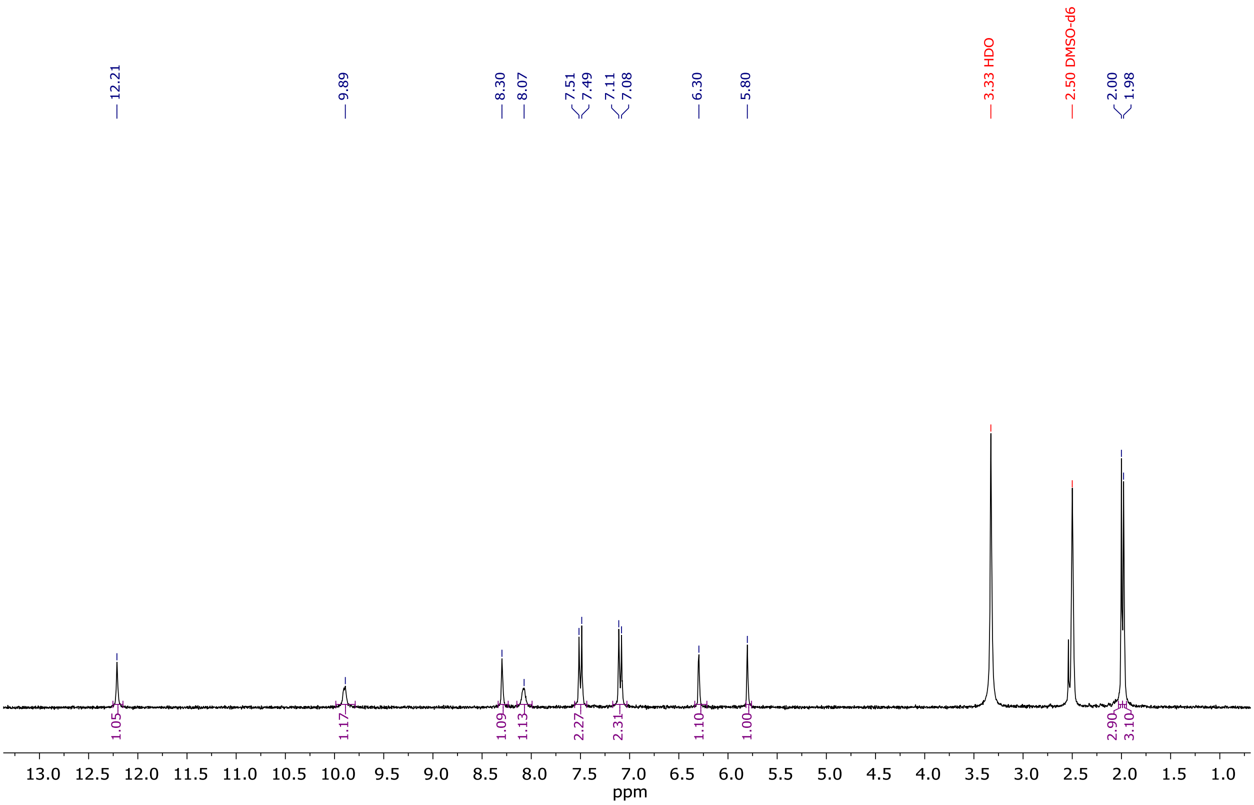

^{13}C NMR spectrum (126 MHz) for **7c**

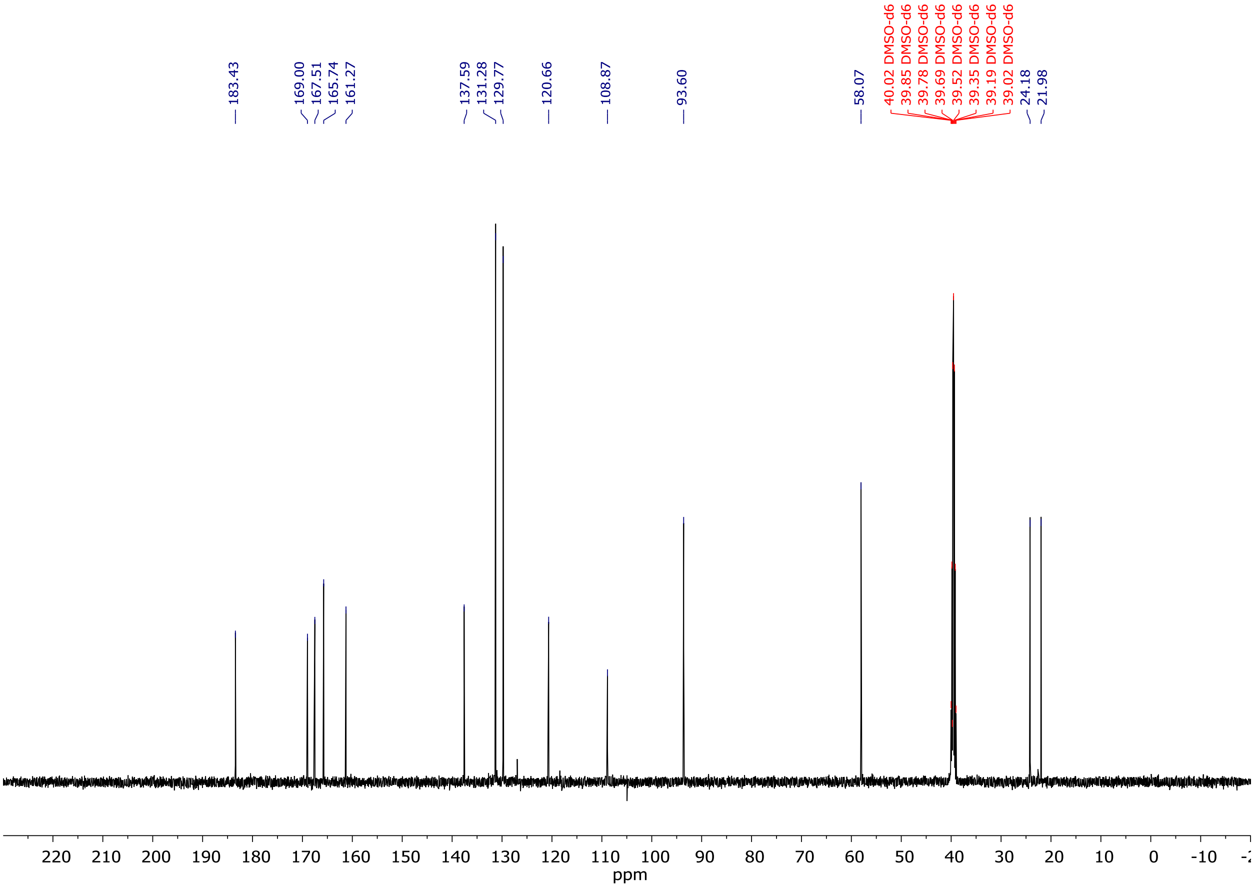

^{1}H NMR spectrum (300 MHz) for **7d**

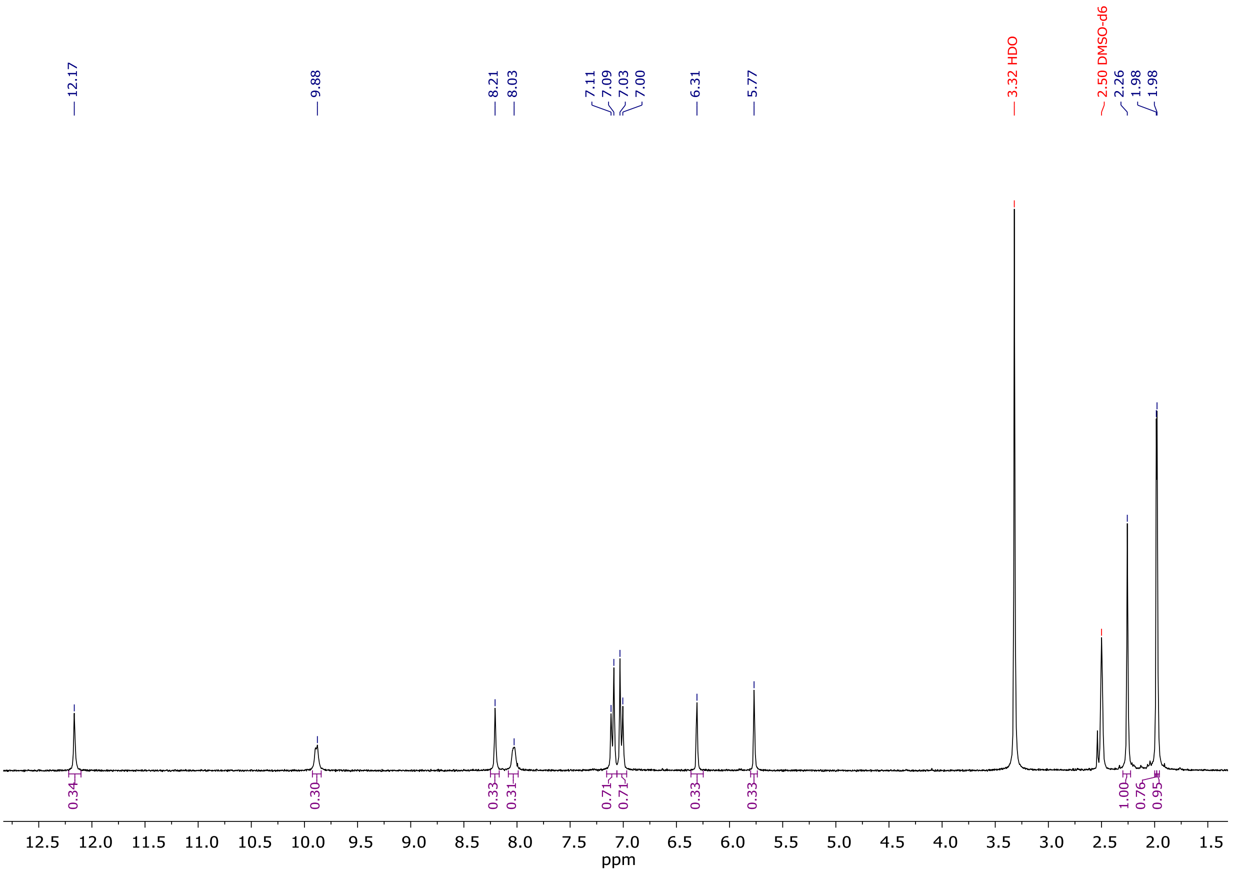

^{13}C NMR spectrum (75 MHz) for **7d**

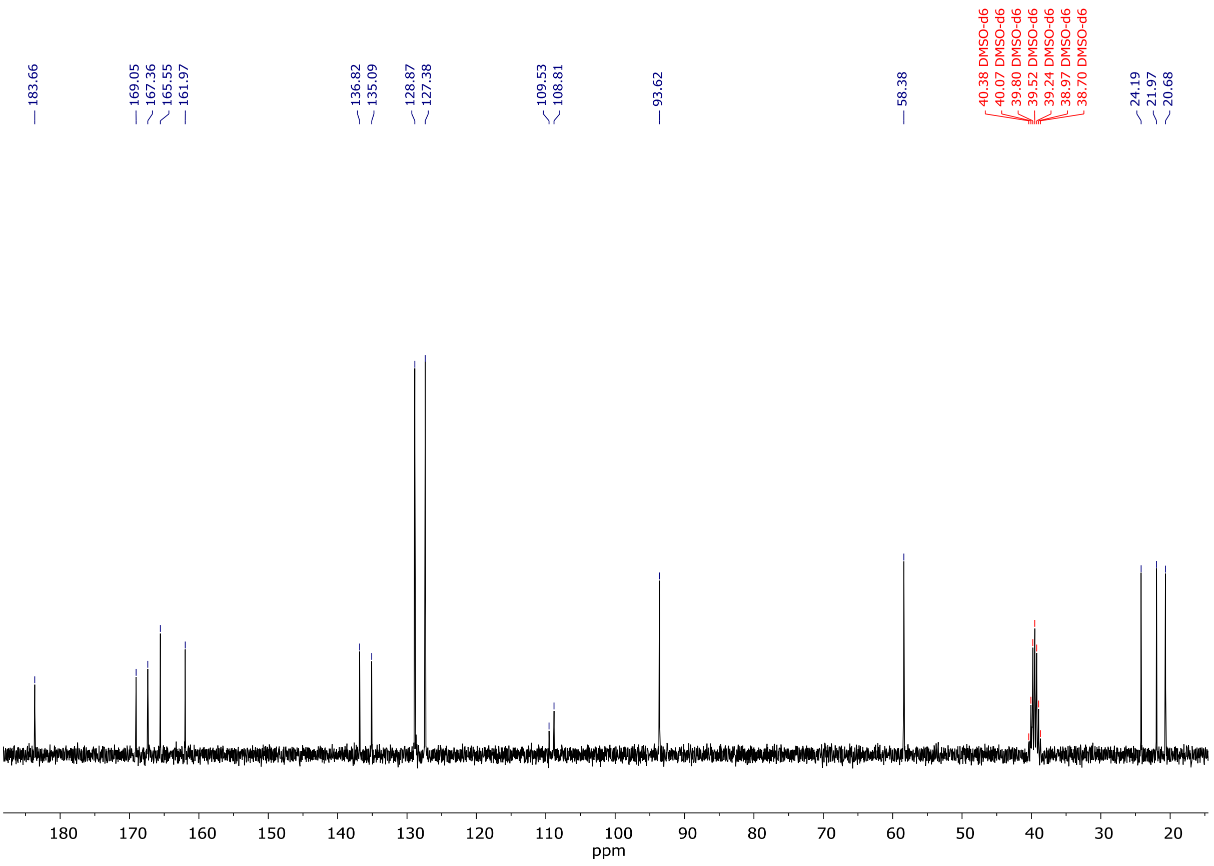

^{1}H NMR spectrum (300 MHz) for **7i**

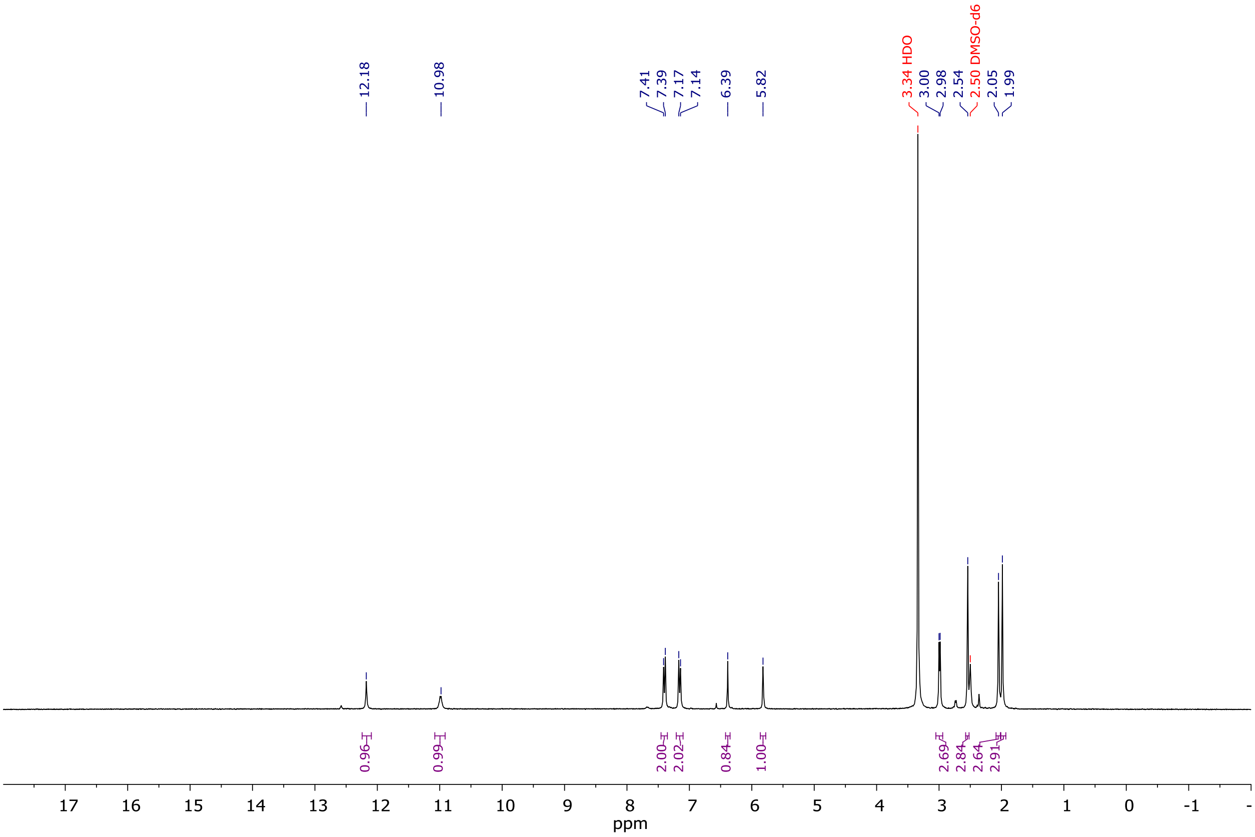

^{13}C NMR spectrum (101 MHz) for **7i**

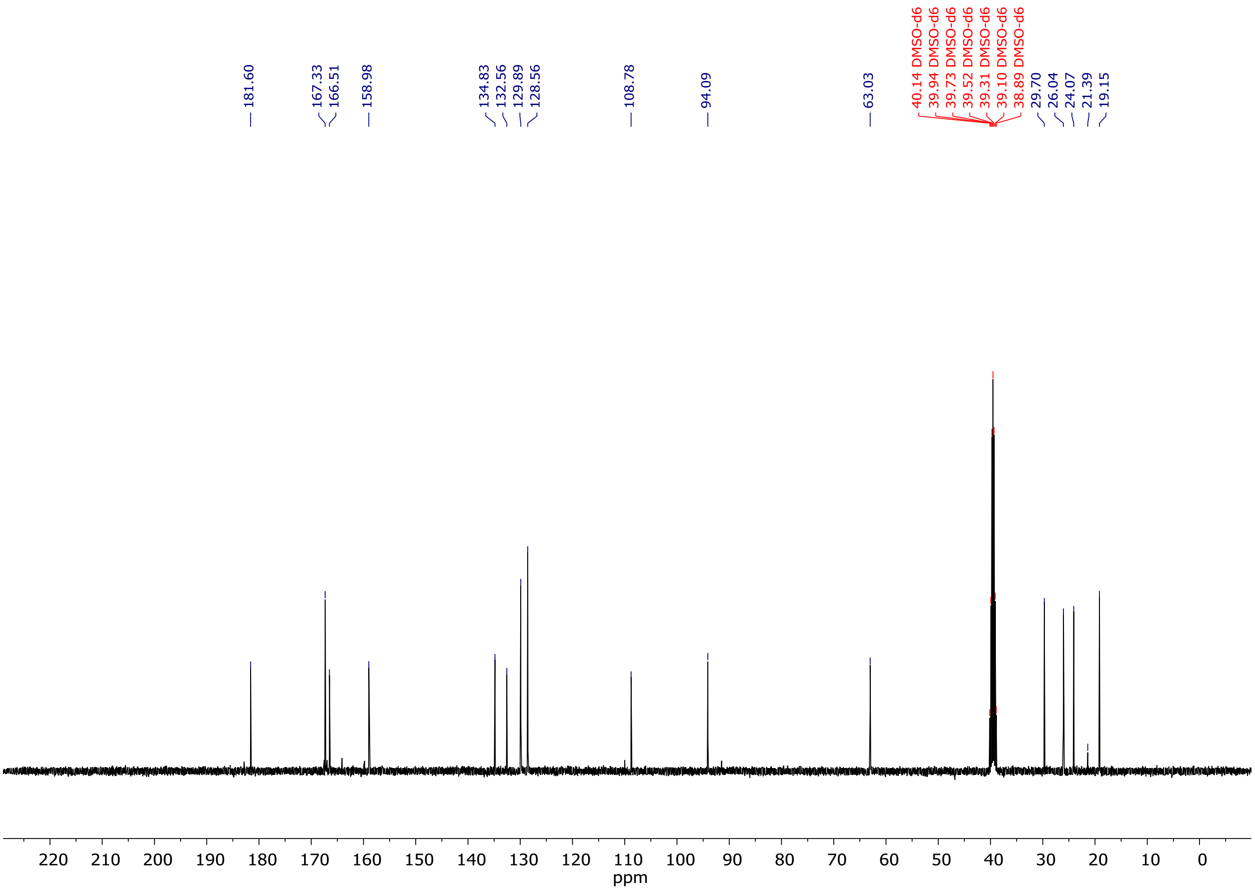

^{1}H NMR spectrum (300 MHz) for **7f**

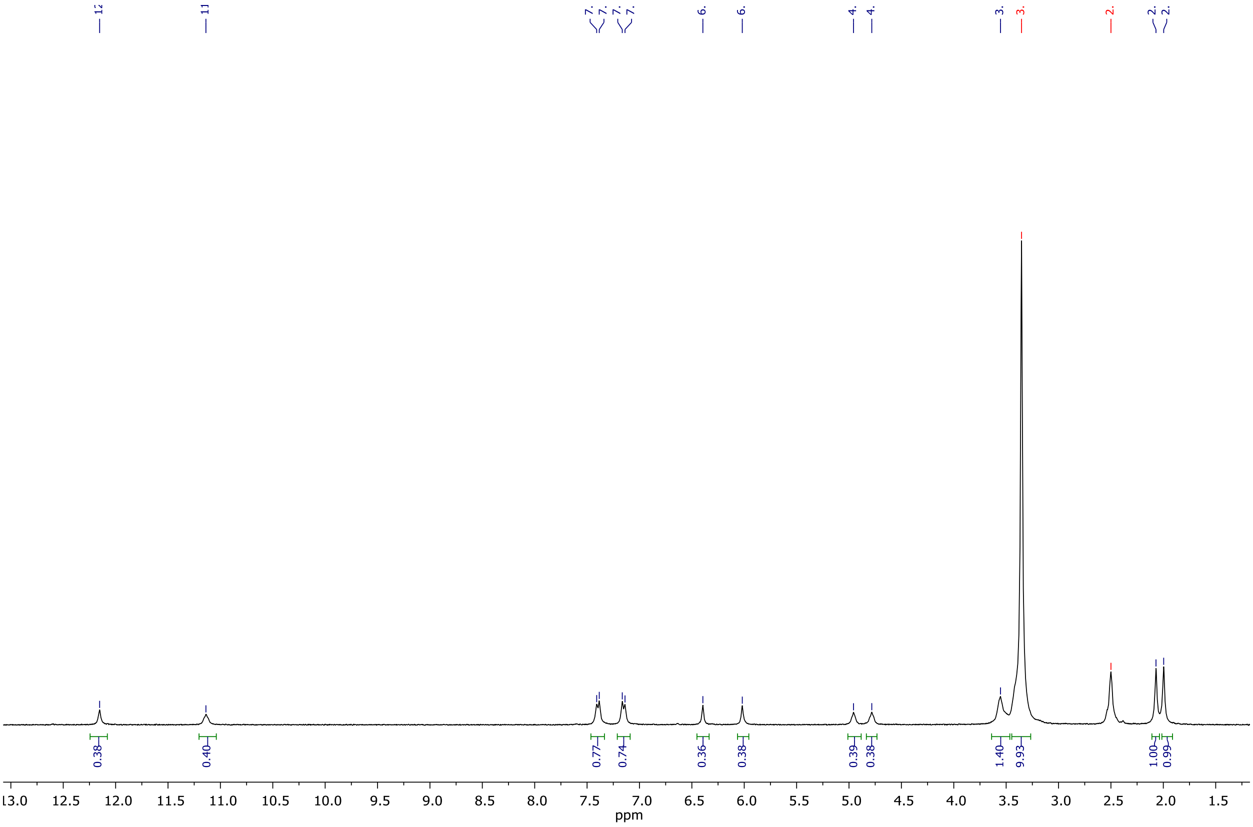

^{13}C NMR spectrum (126 MHz) for **7f**

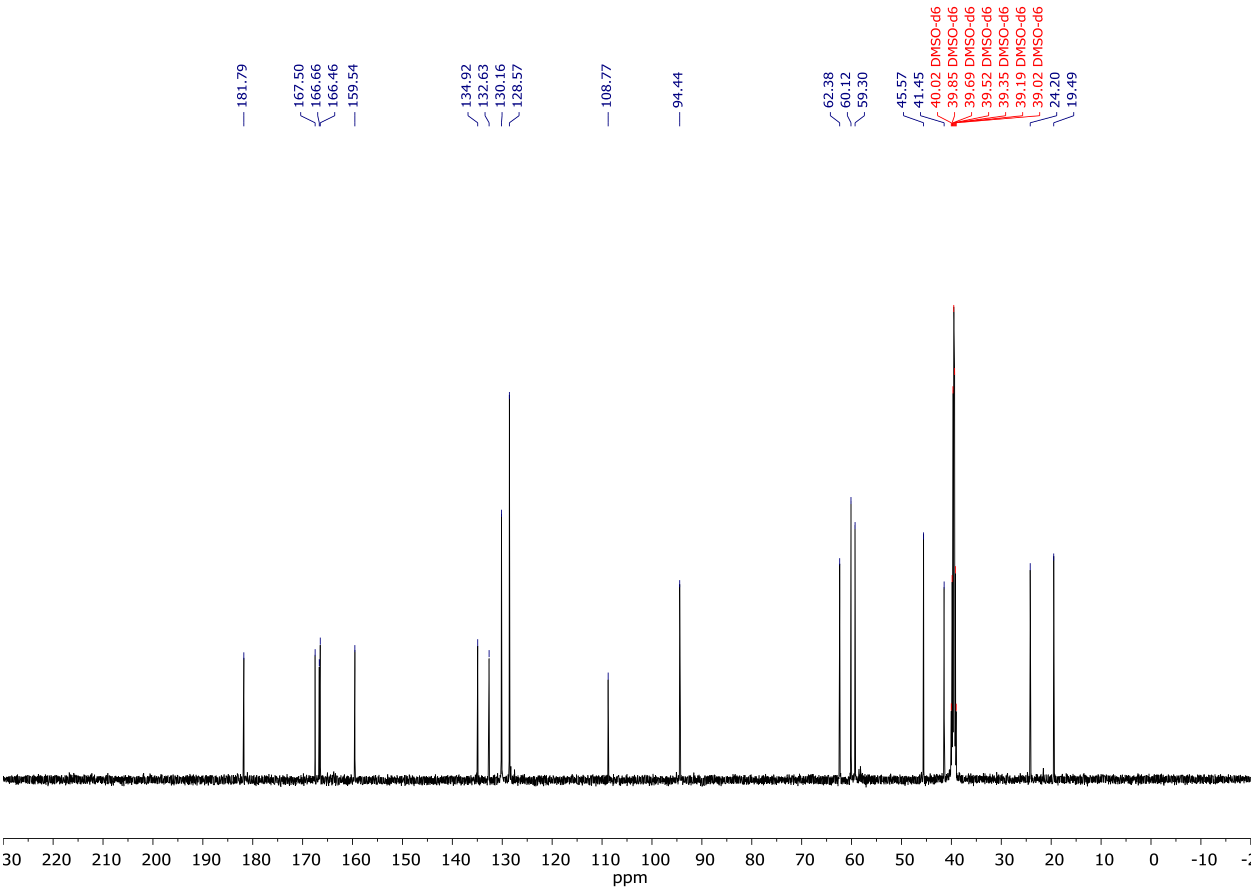

^{1}H NMR spectrum (300 MHz) for **1a**

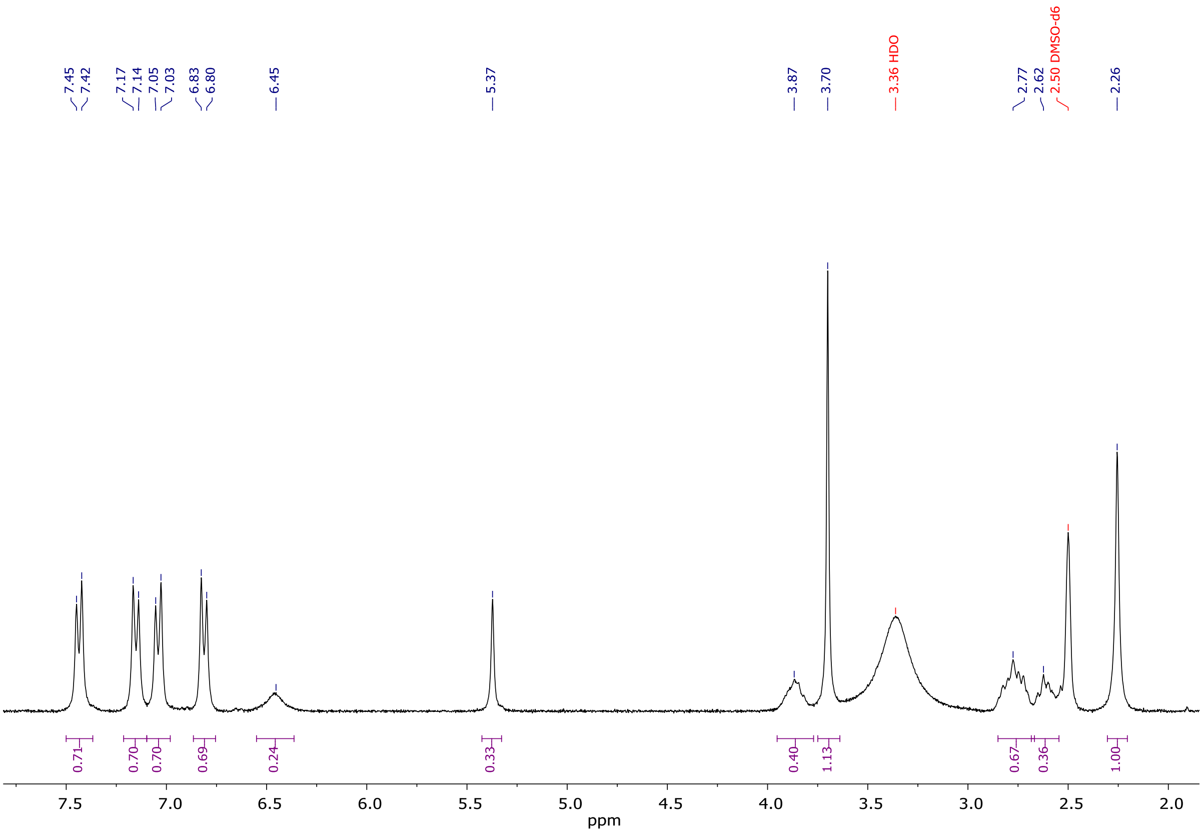

^{13}C NMR spectrum (75 MHz) for **1a**

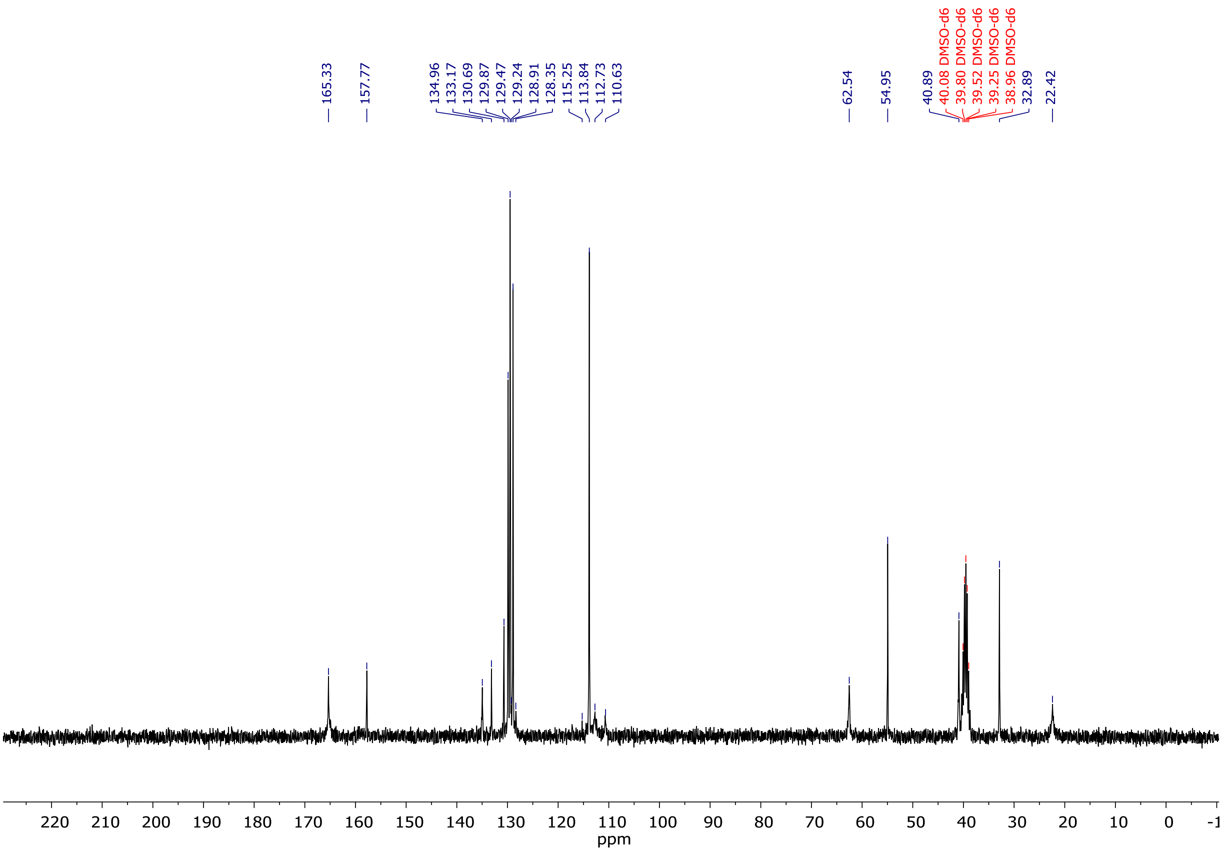

^{1}H NMR spectrum (300 MHz) for **1b**

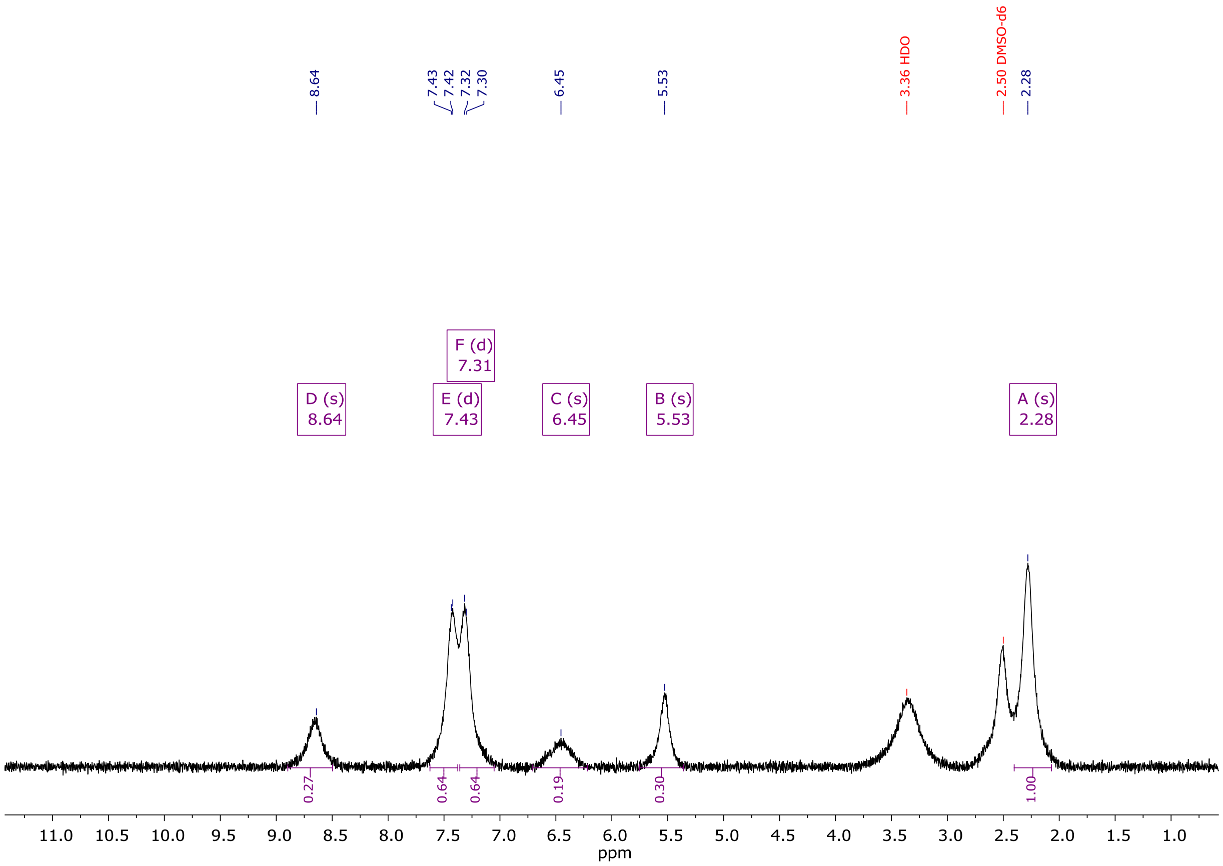

^{13}C NMR spectrum (75 MHz) for **1b**

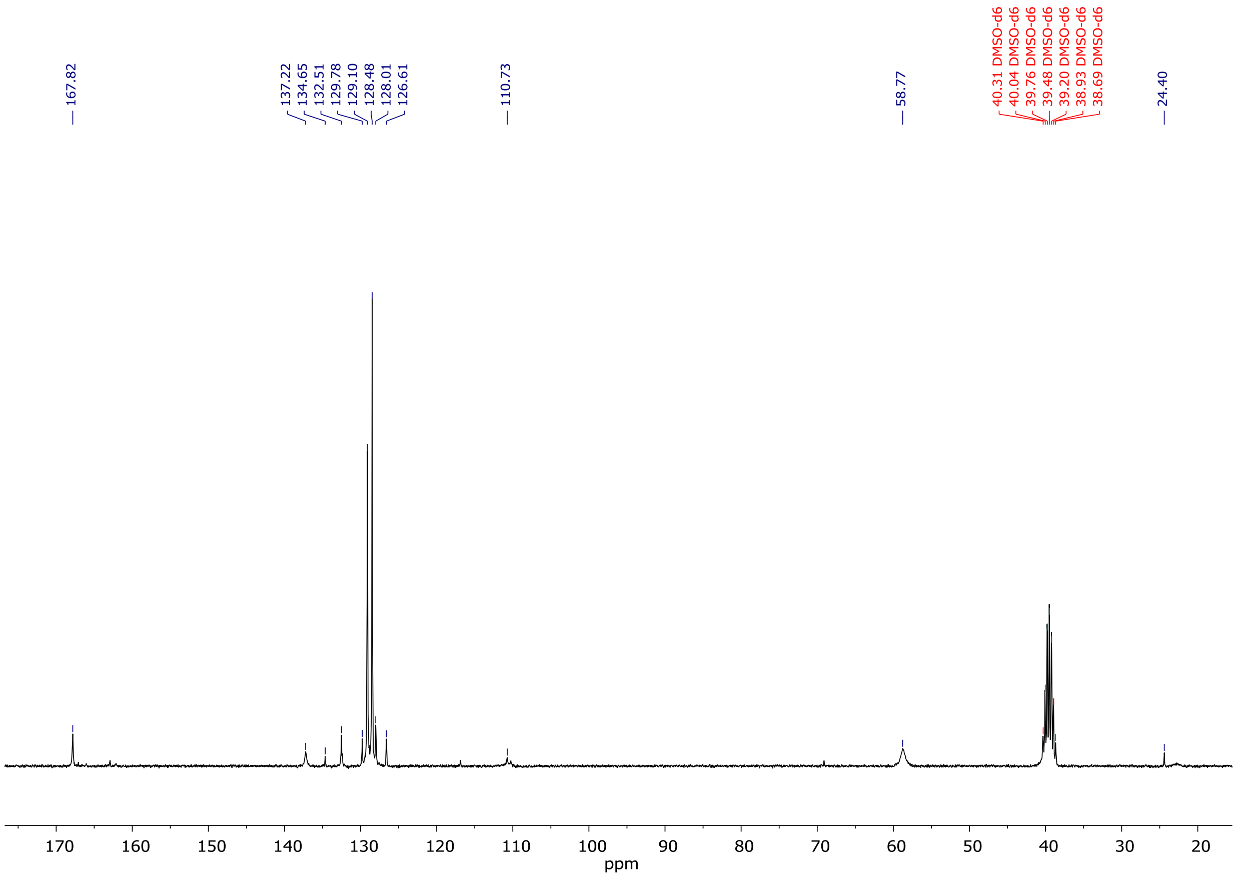

^{1}H NMR spectrum (300 MHz) for **1c**

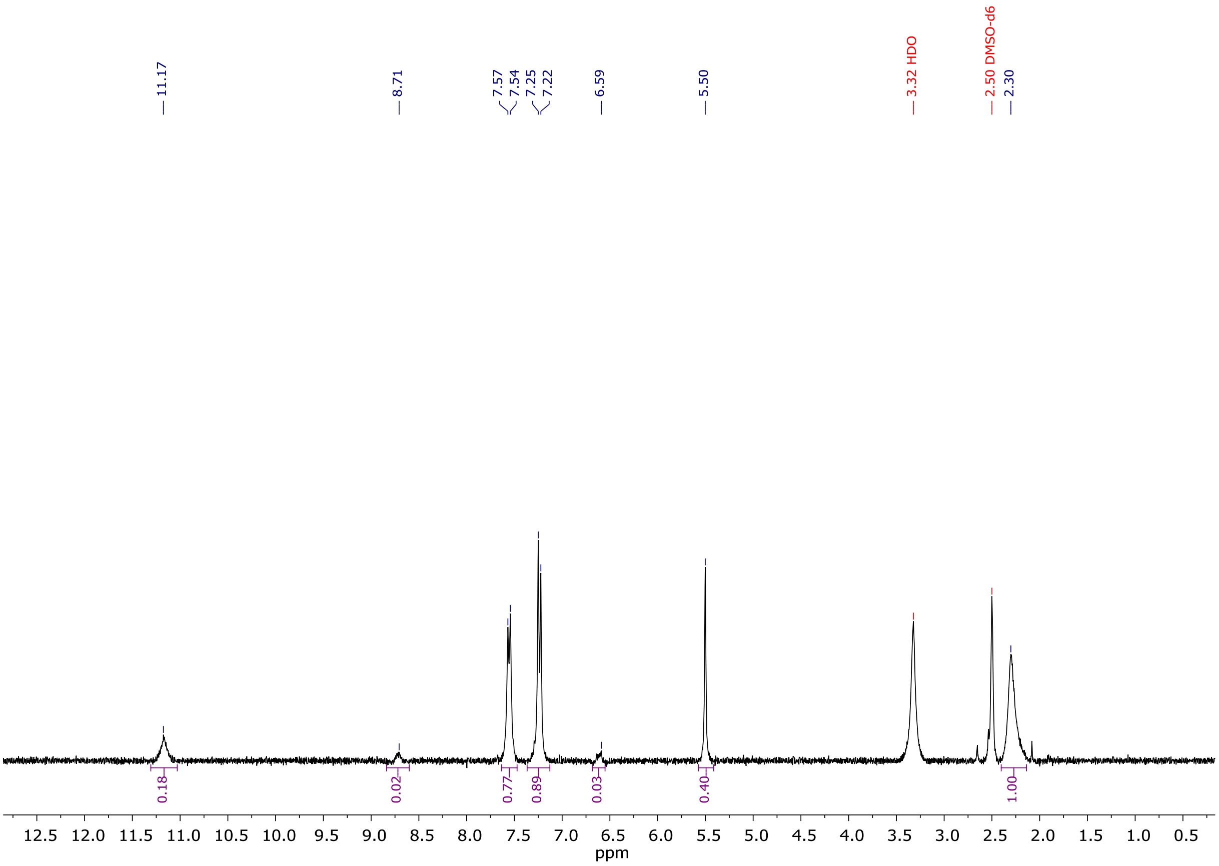

^{13}C NMR spectrum (151 MHz) for **1c**

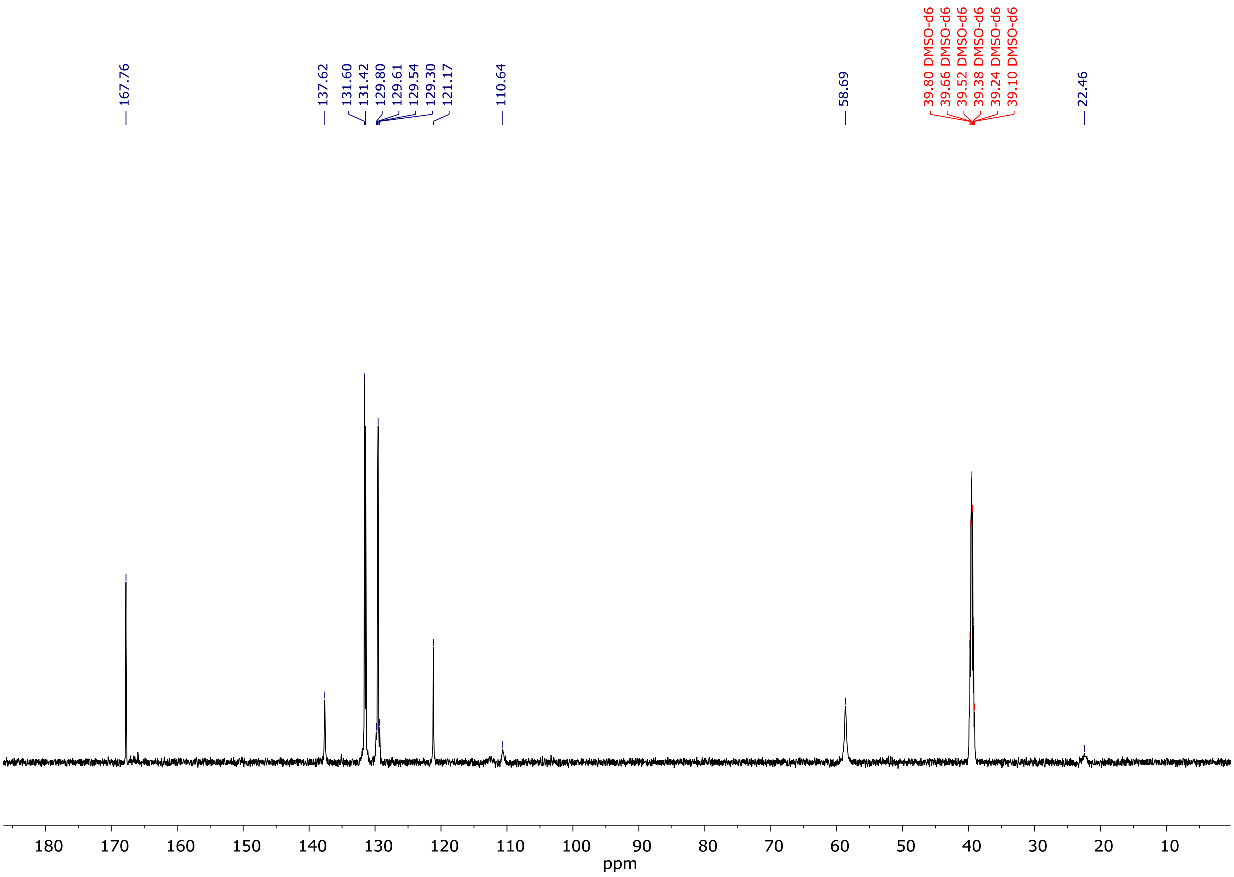

^{1}H NMR spectrum (400 MHz) for **1d**

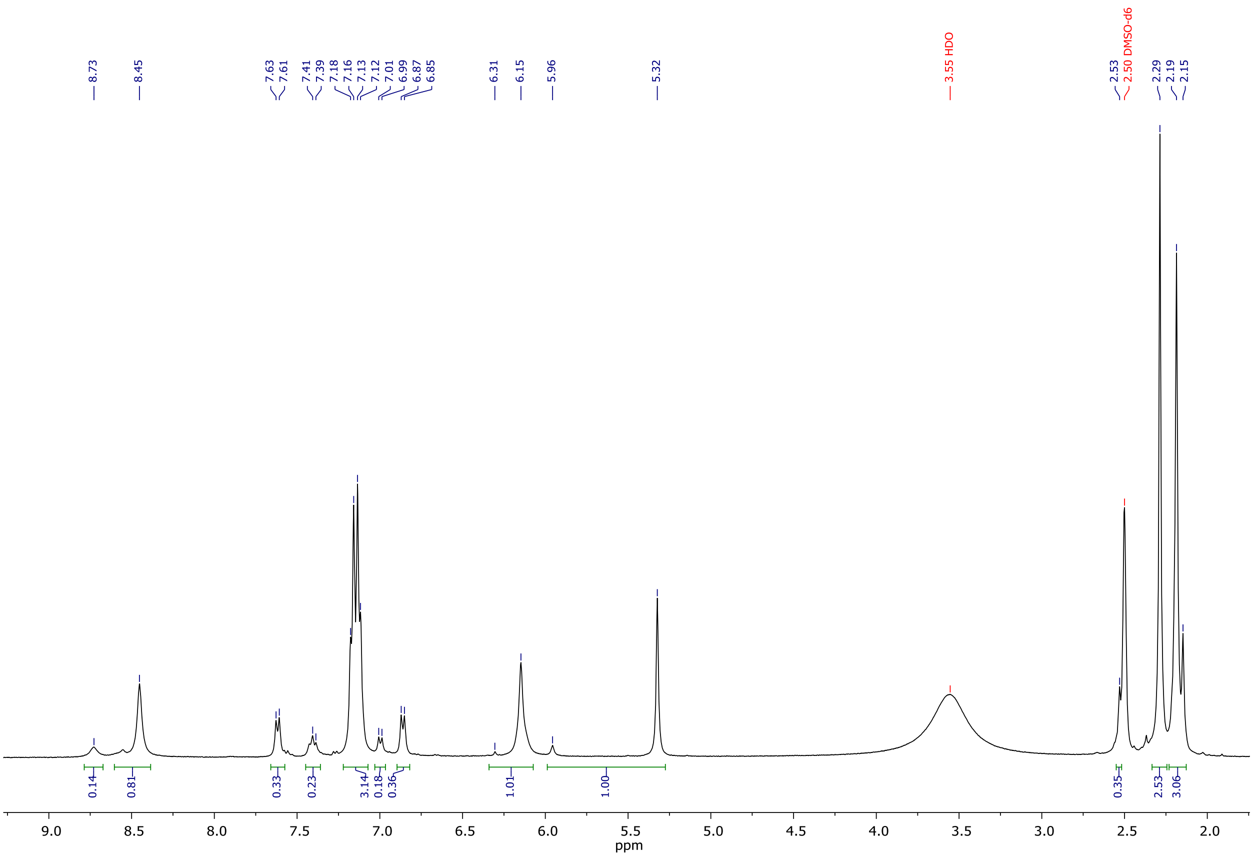

^{13}C NMR spectrum (101 MHz) for **1d**

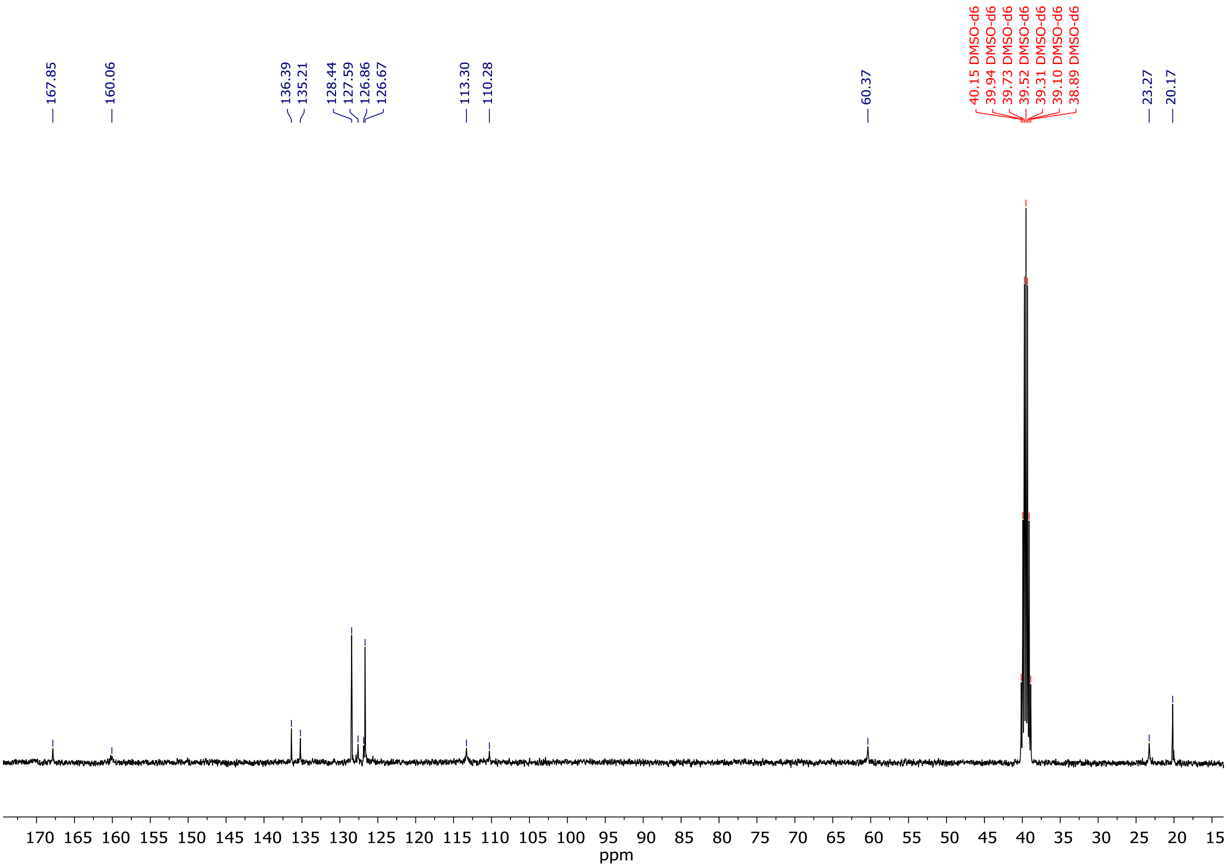

^{1}H NMR spectrum (300 MHz) for **1e**

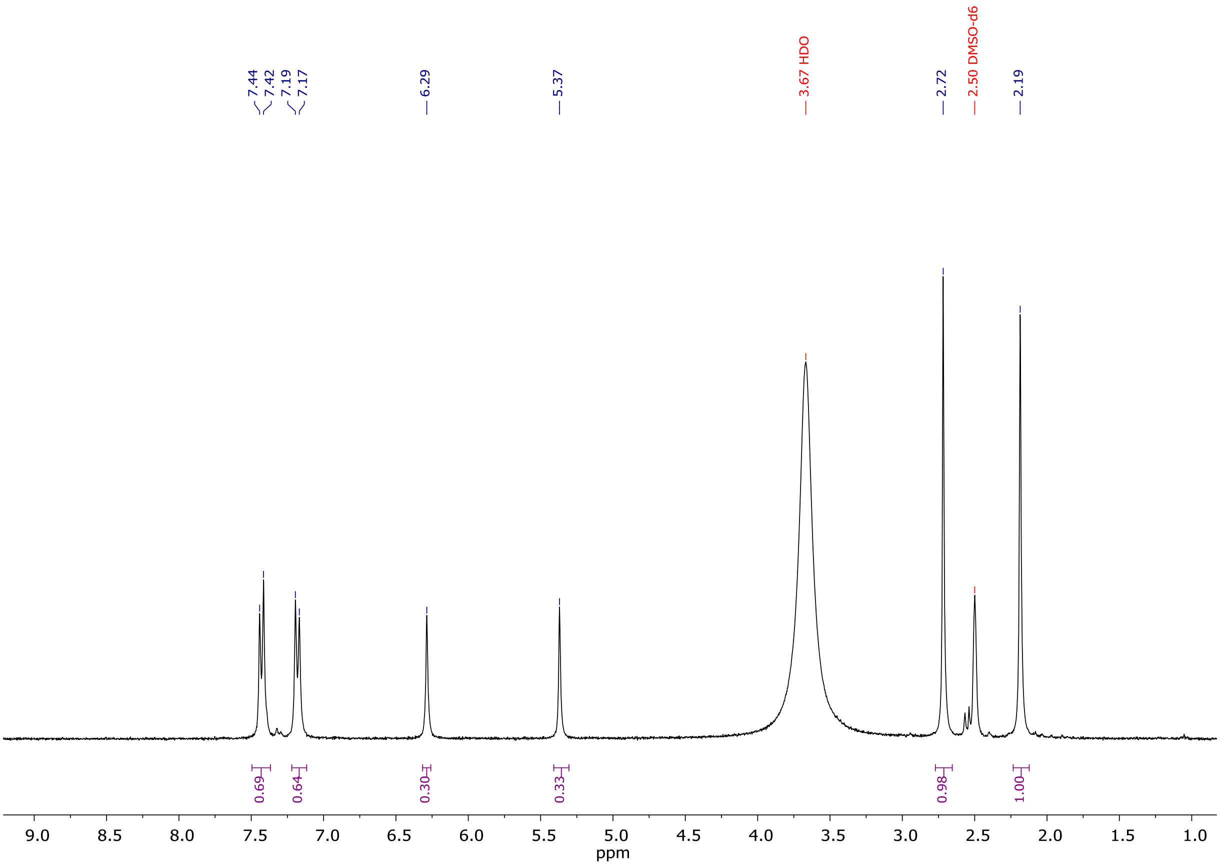

^{13}C NMR spectrum (101 MHz) for **1e**

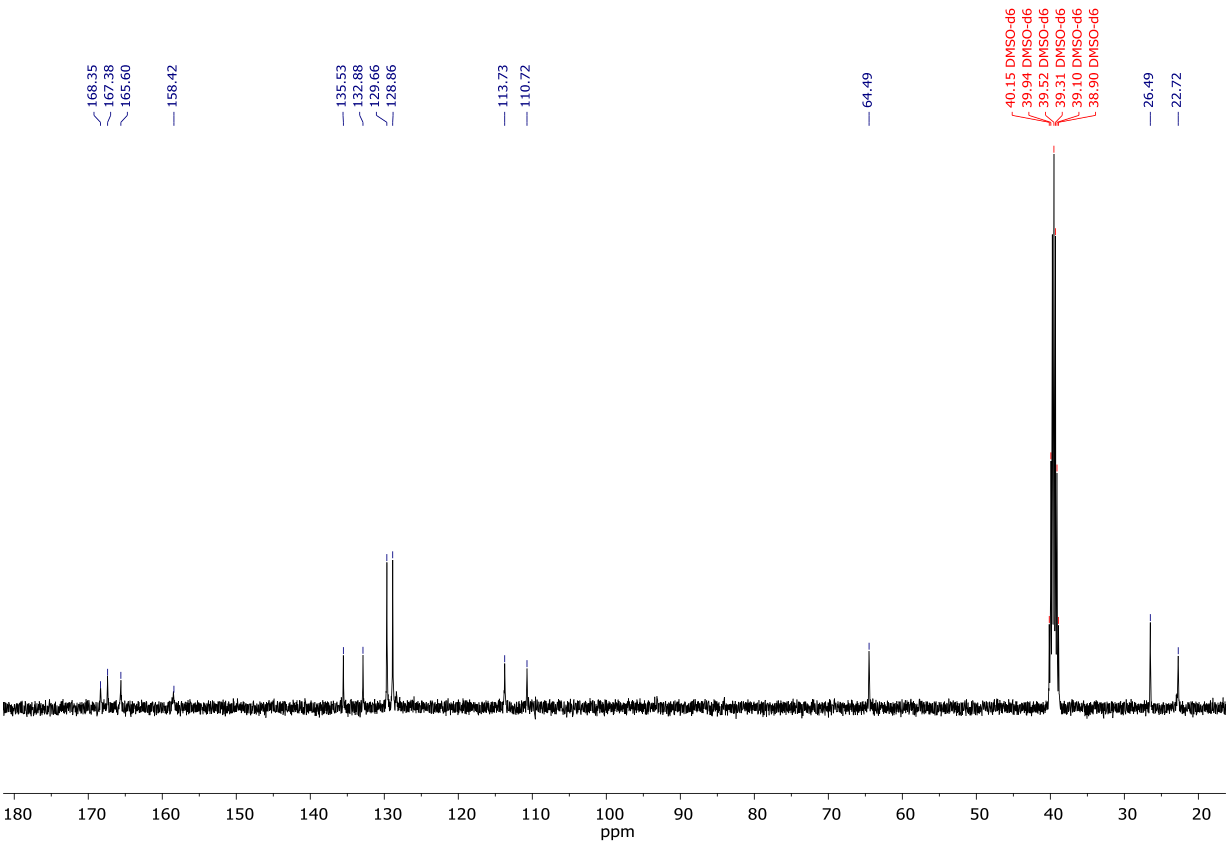

^{1}H NMR spectrum (300 MHz) for **1f**

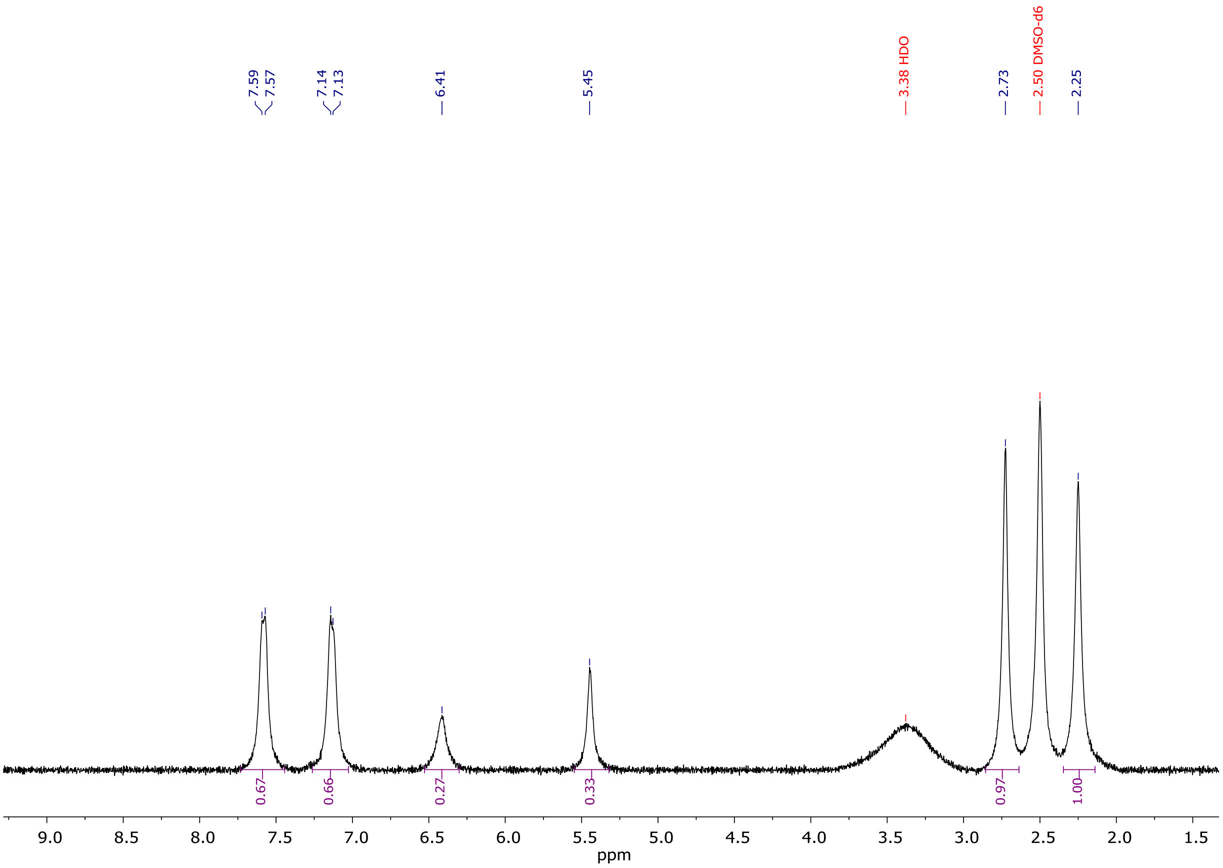

^{13}C NMR spectrum (75 MHz) for **1f**

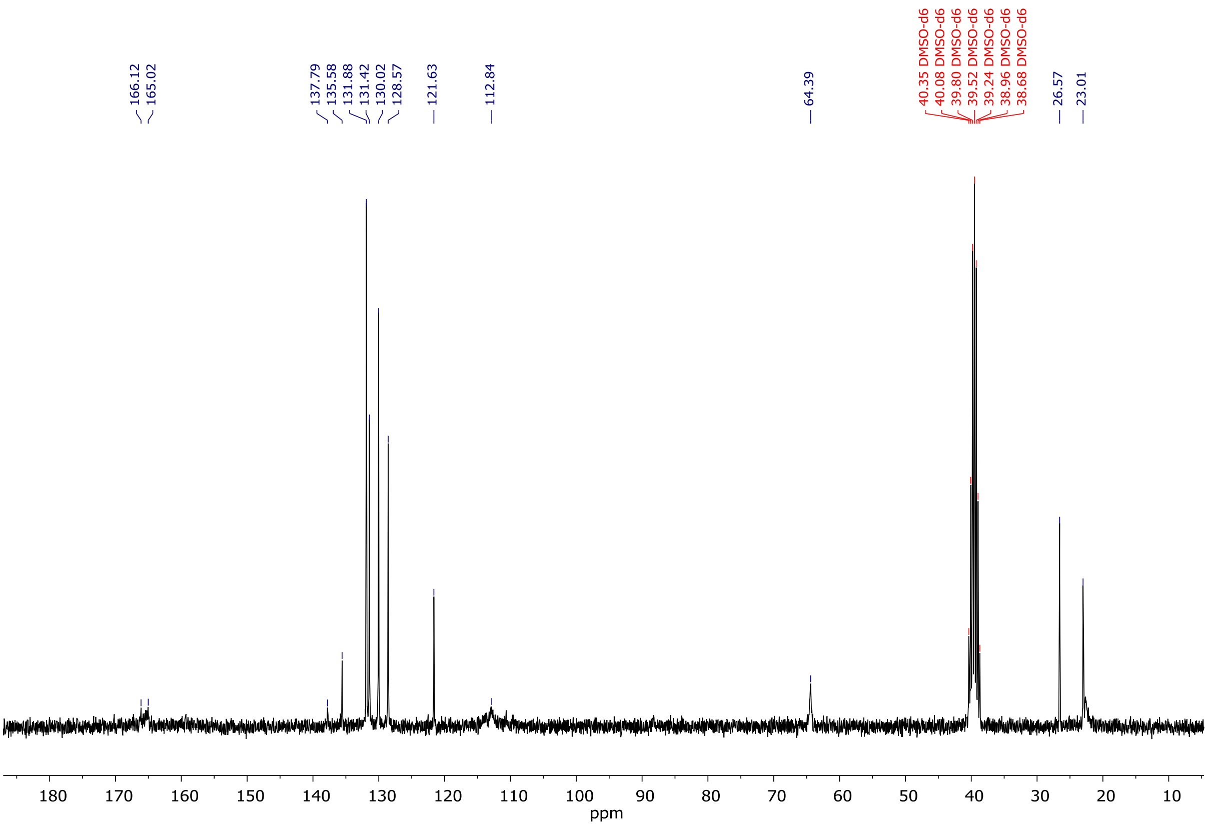

^{1}H NMR spectrum (300 MHz) for **1g**

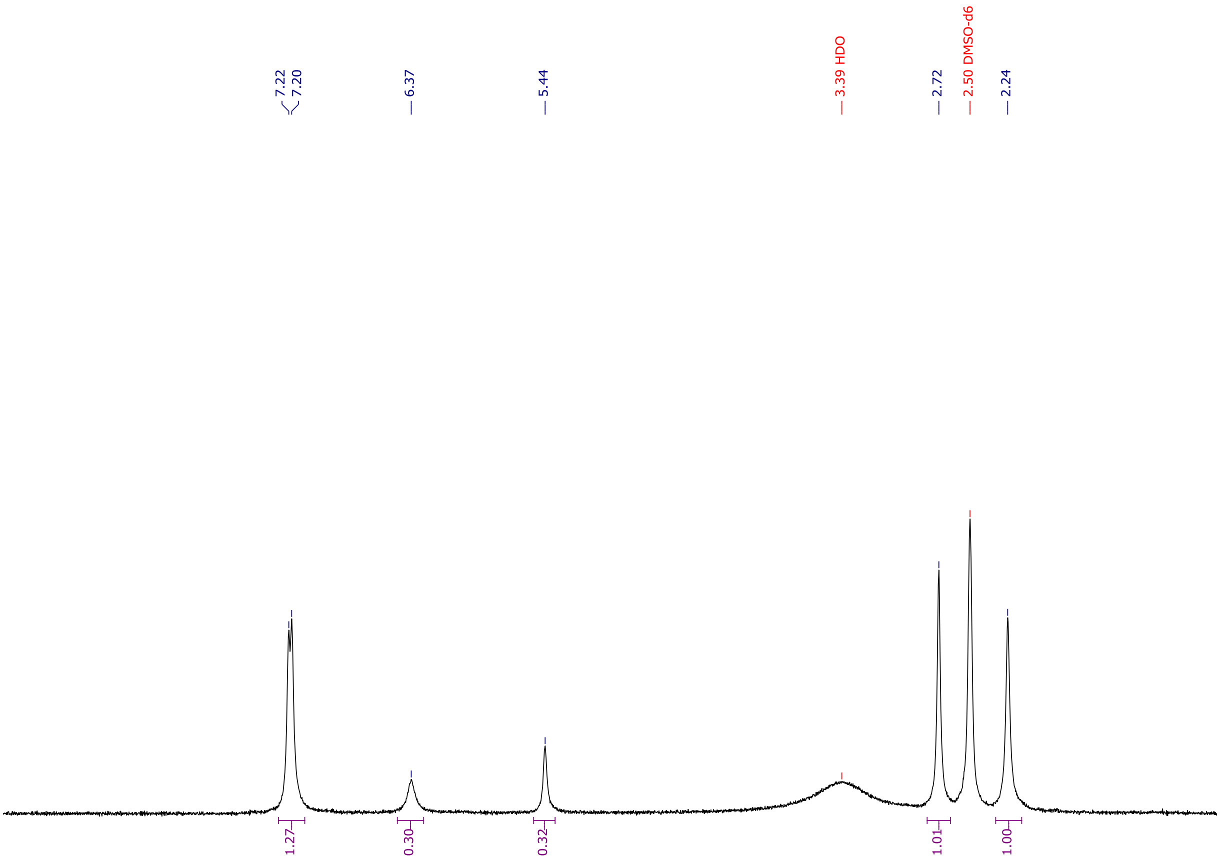

^{13}C NMR spectrum (75 MHz) for **1g**

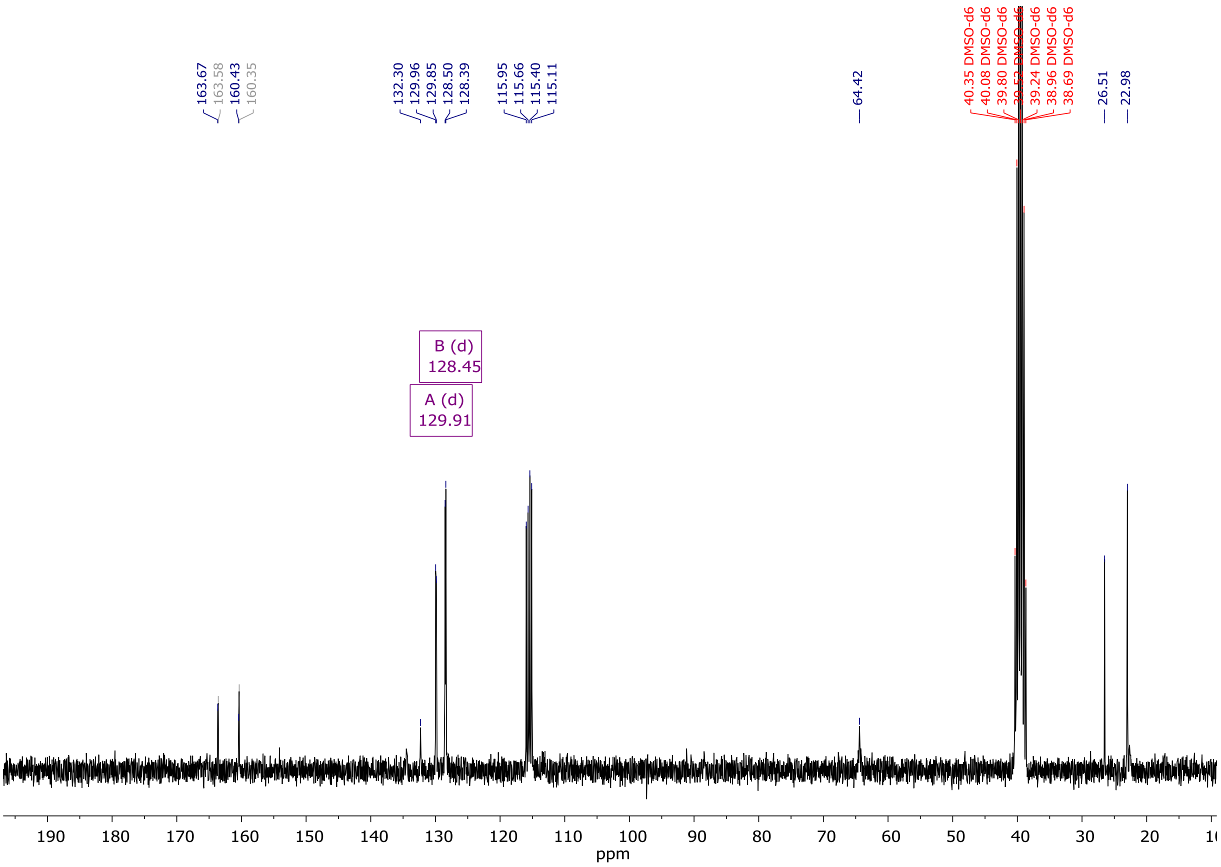

^{1}H NMR spectrum (300 MHz) for **1h**

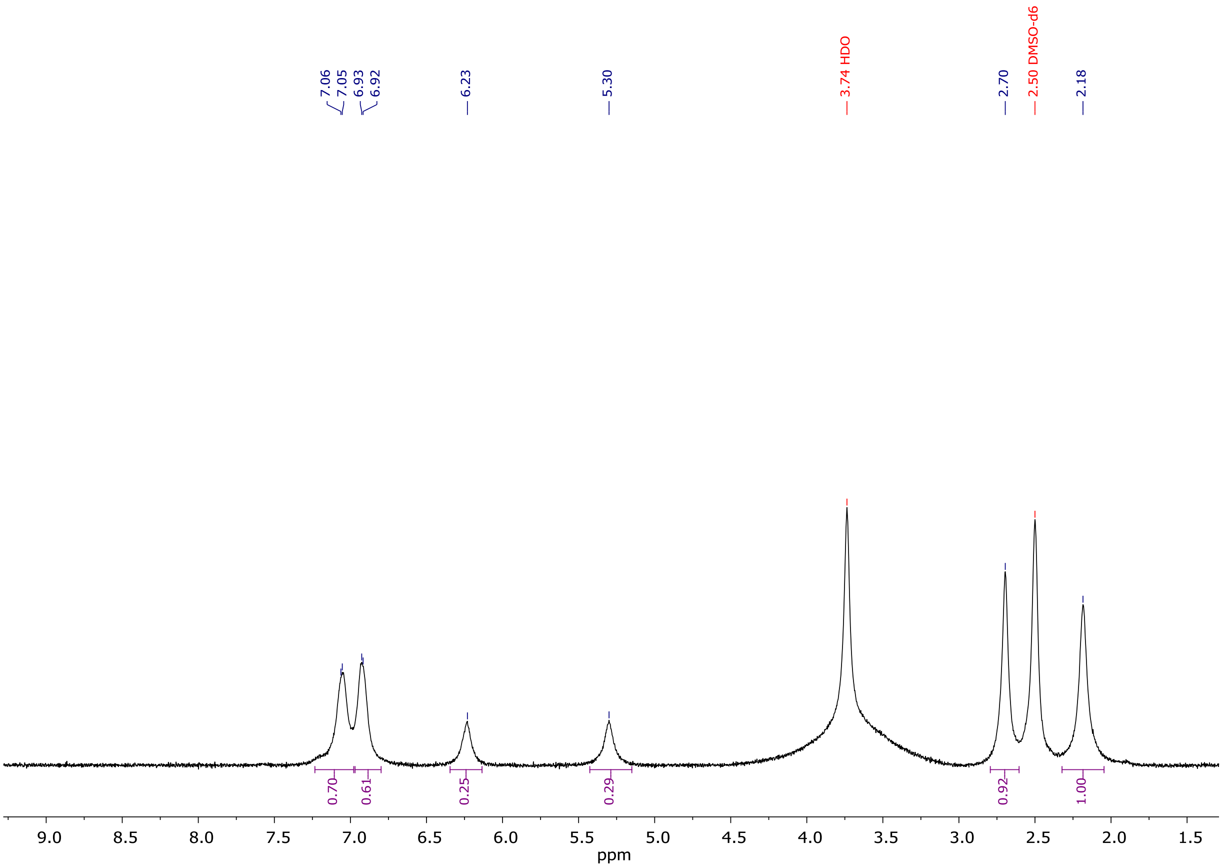

^{13}C NMR spectrum (151 MHz) for **1h**

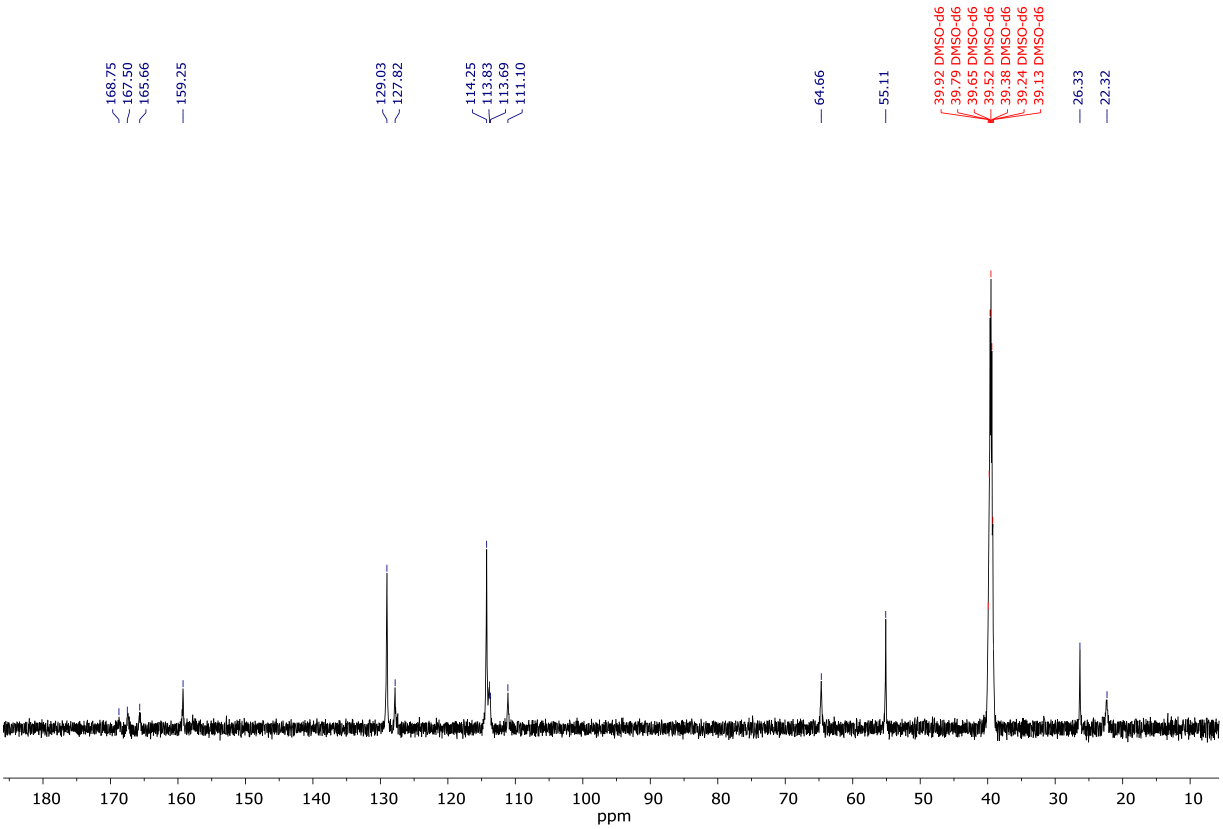

^{1}H NMR spectrum (300 MHz) for **1i**

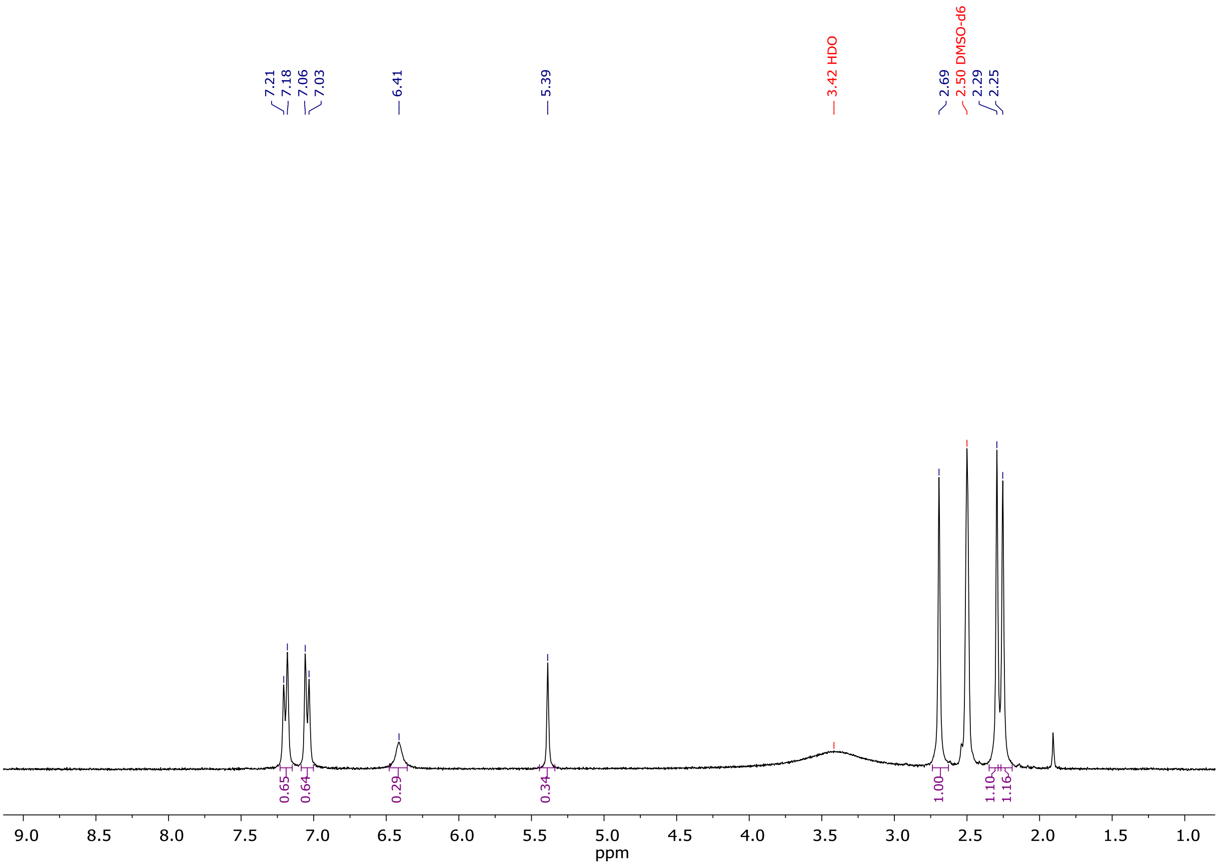

^{13}C NMR spectrum (75 MHz) for **1i**

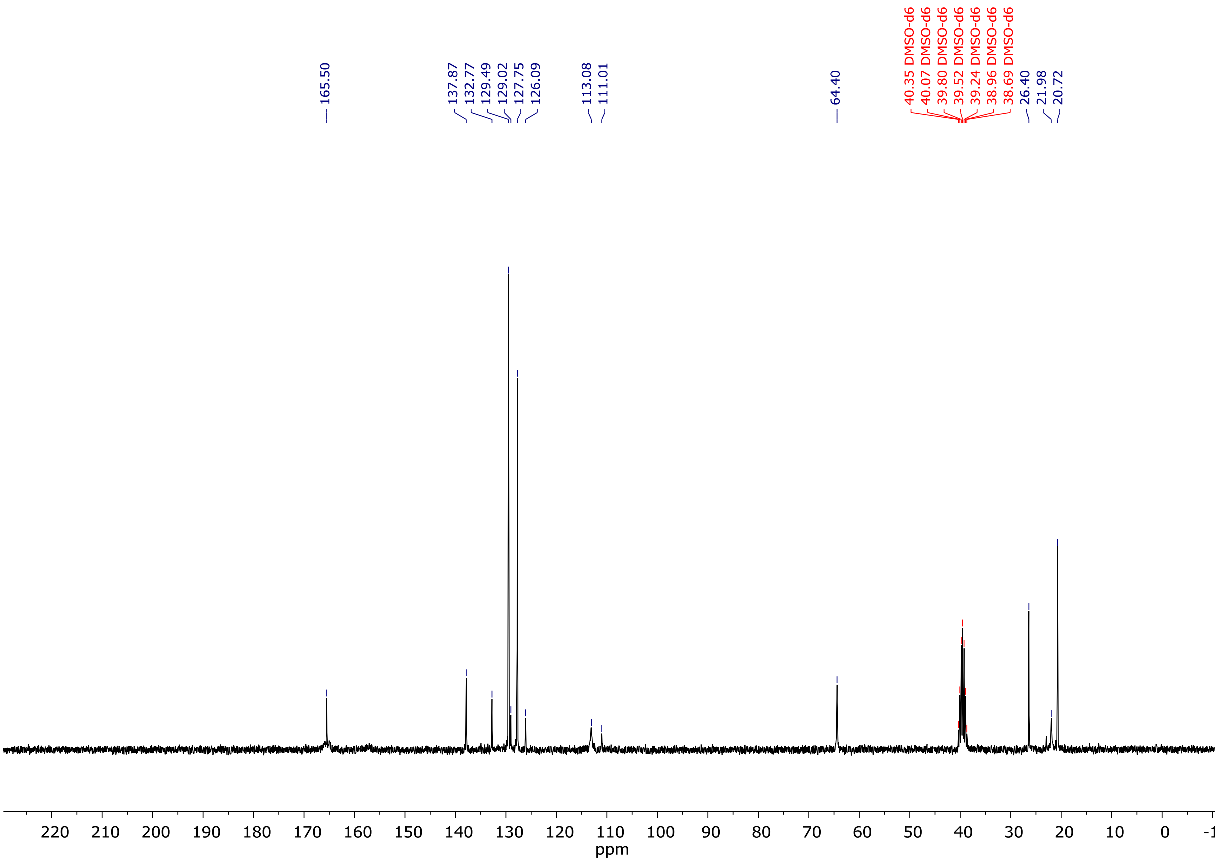

^{1}H NMR spectrum (300 MHz) for **1j**

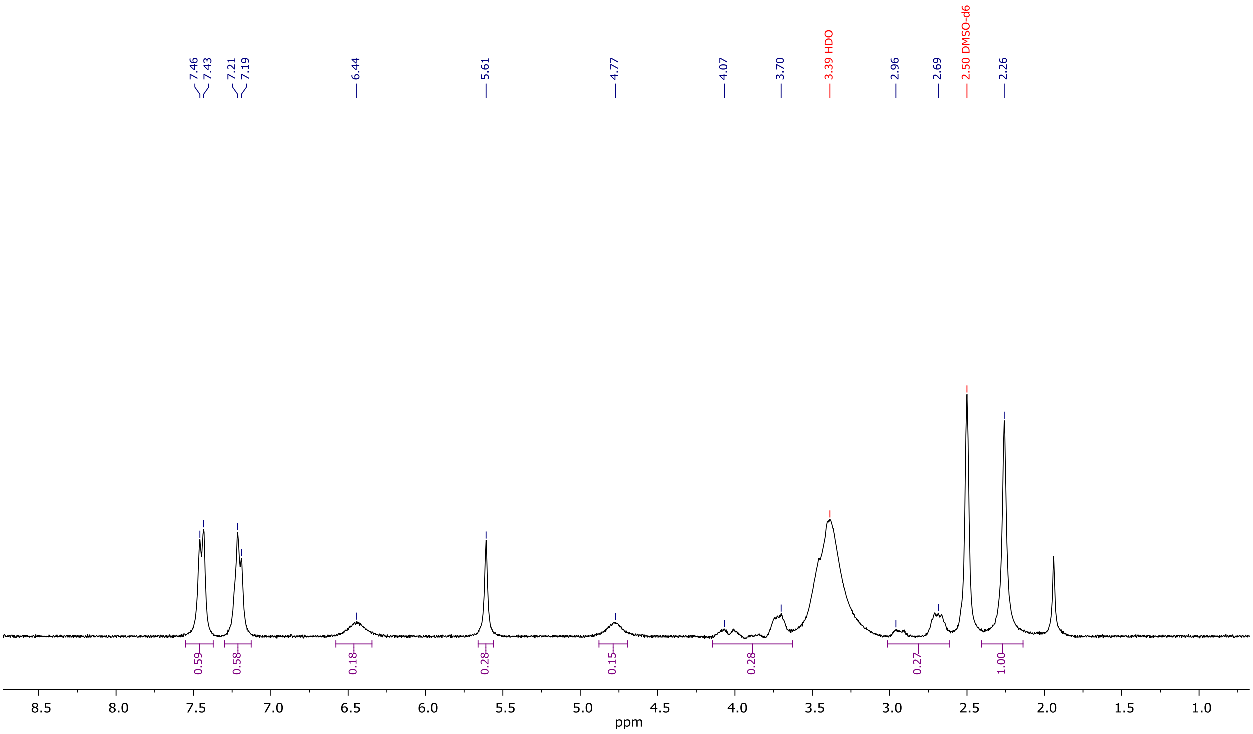

^{13}C NMR spectrum (126 MHz) for **1j**

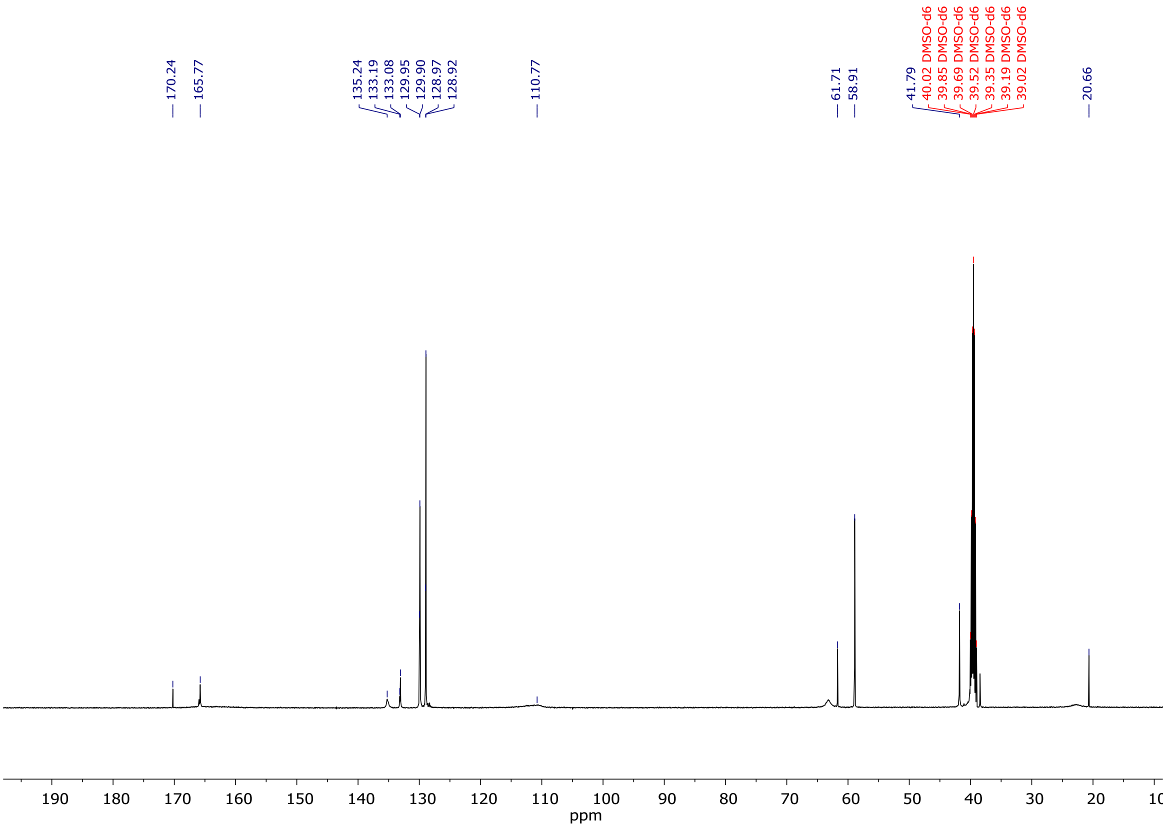

5. HRMS for all compounds.

HRMS for **2a**

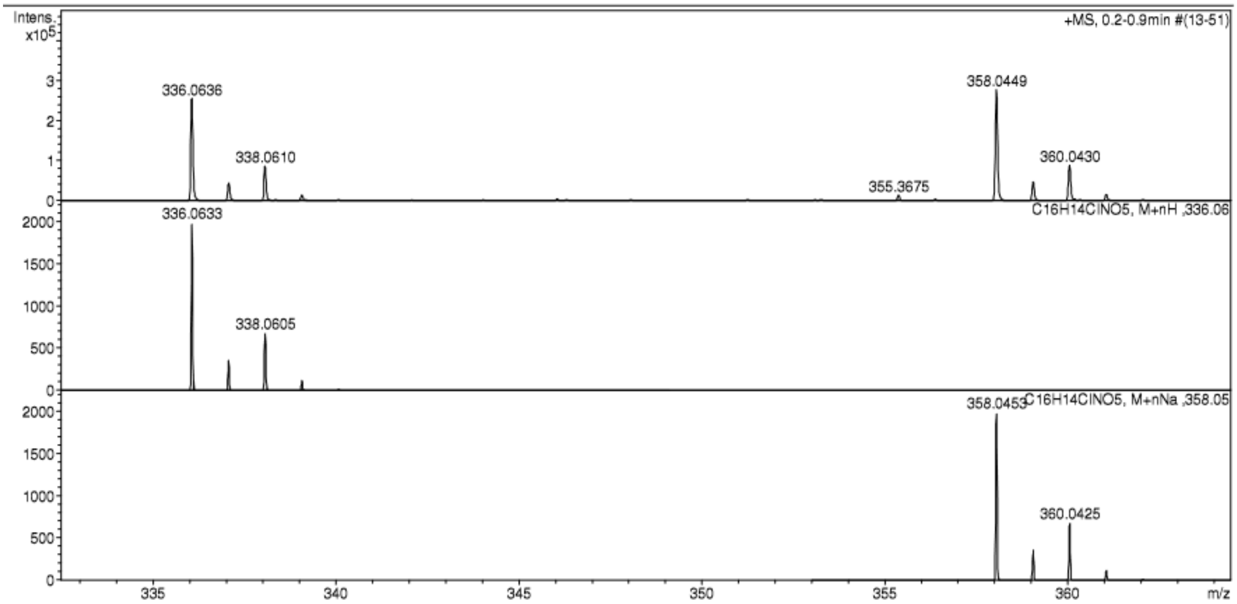

HRMS for **2b**

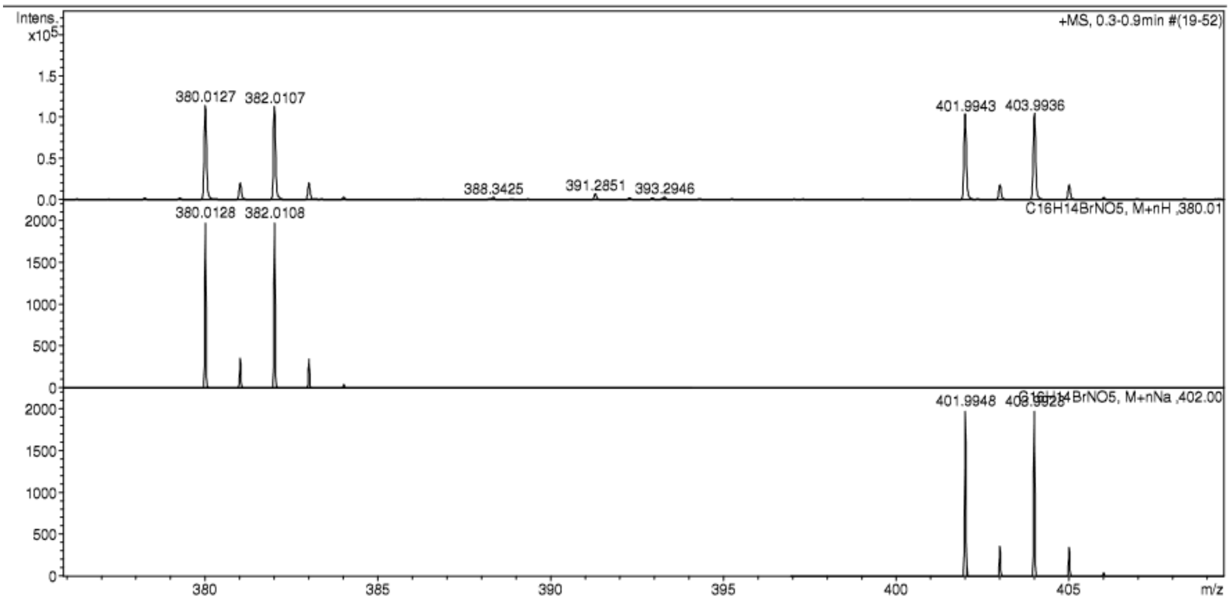

HRMS for **2c**

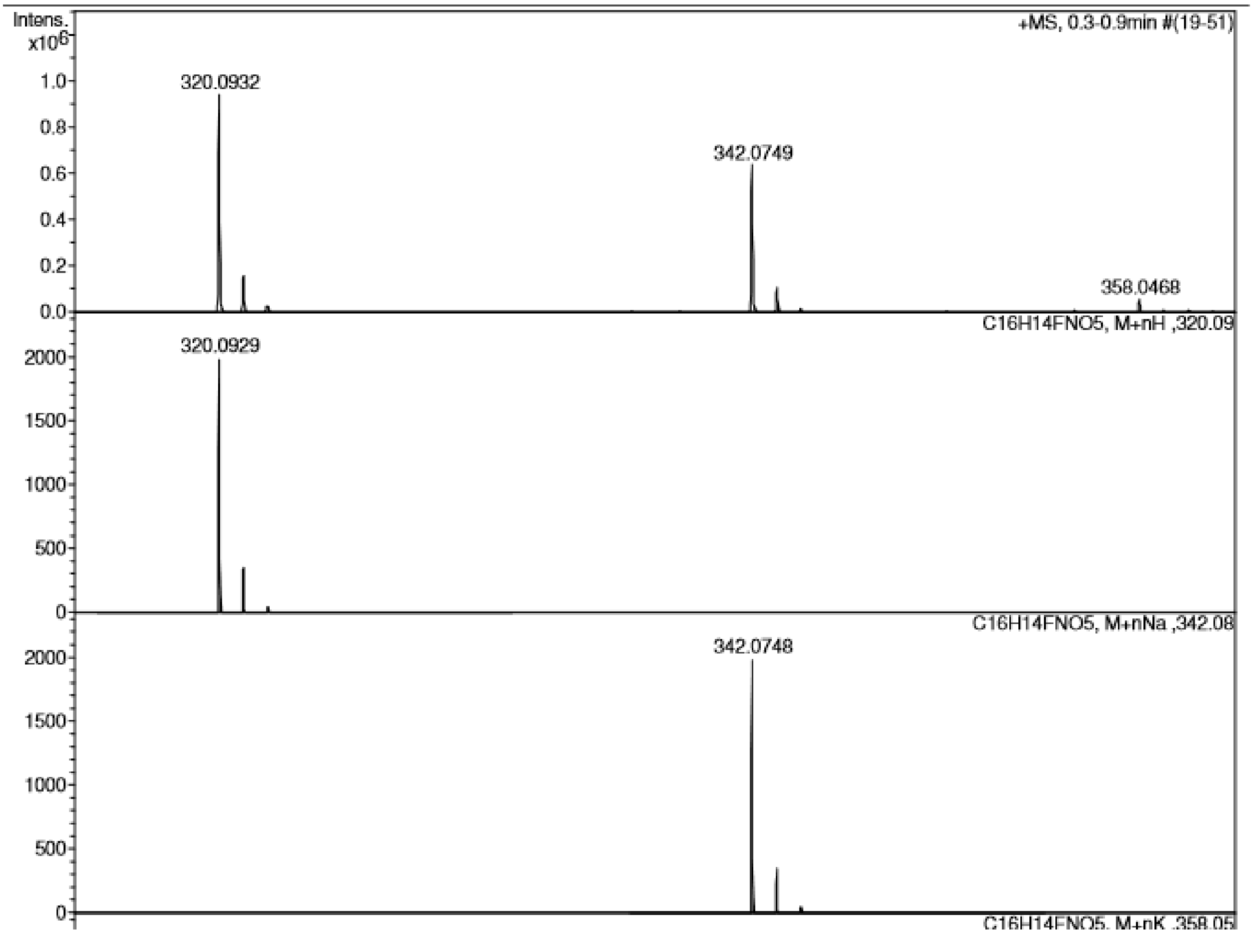

HRMS for **2d**

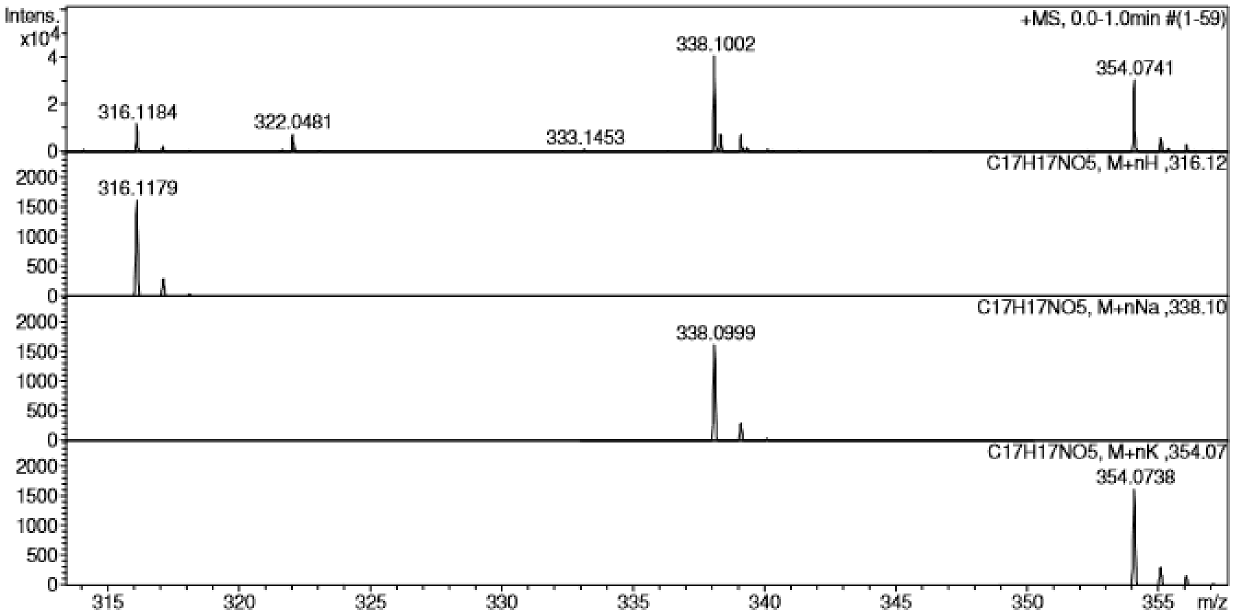

HRMS for **2e**

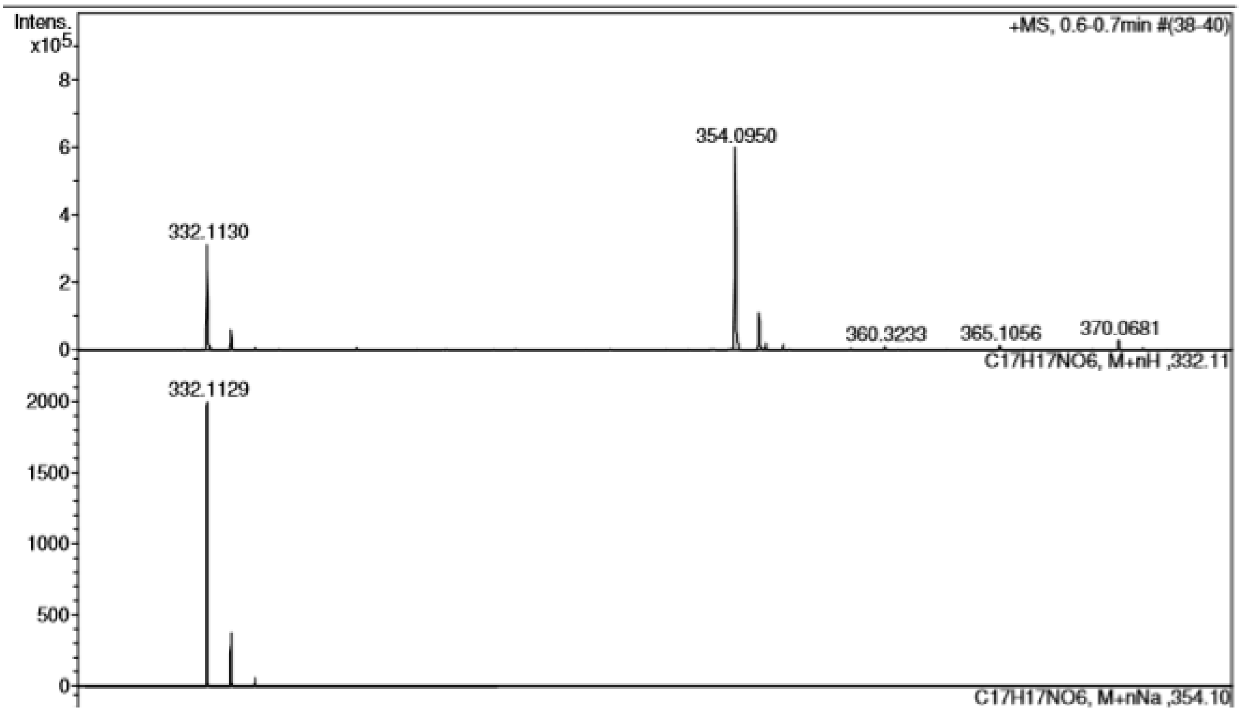

HRMS for **7b**

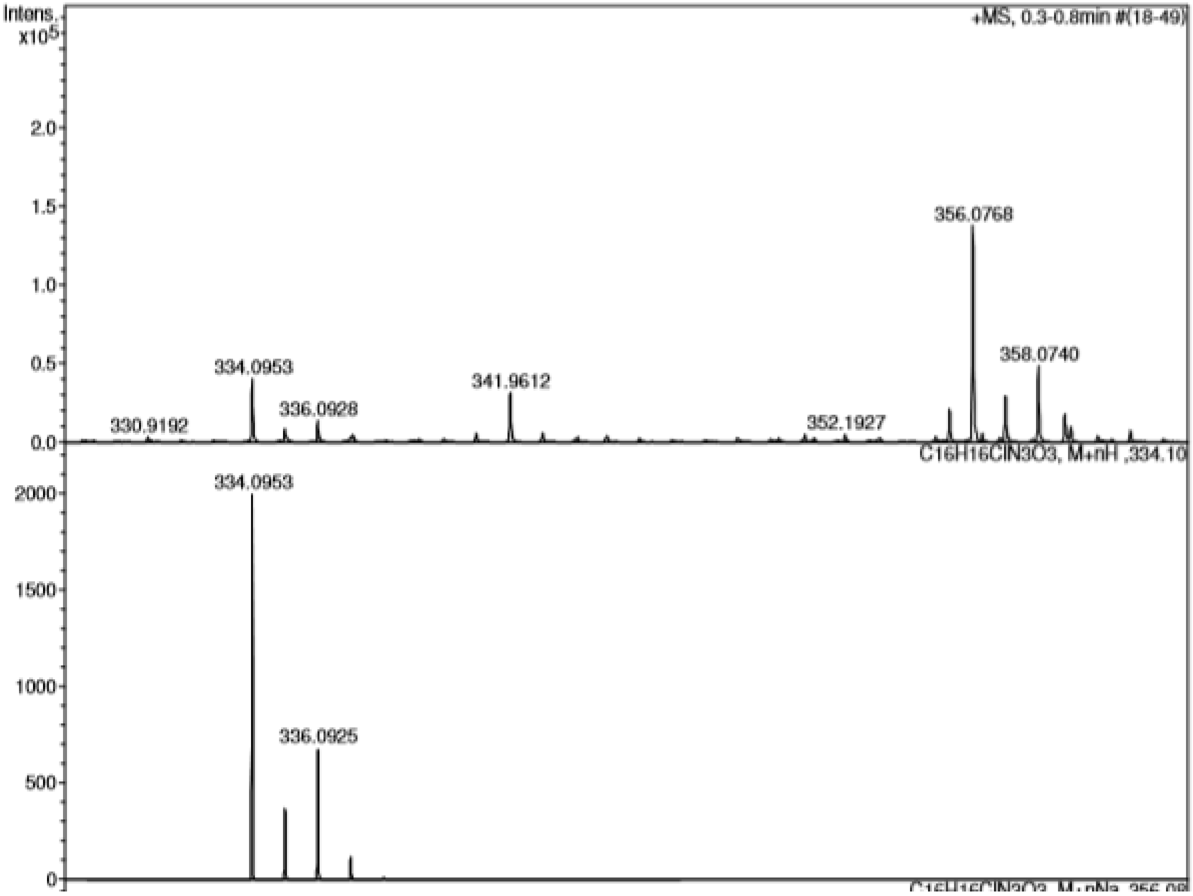

HRMS for **7c**

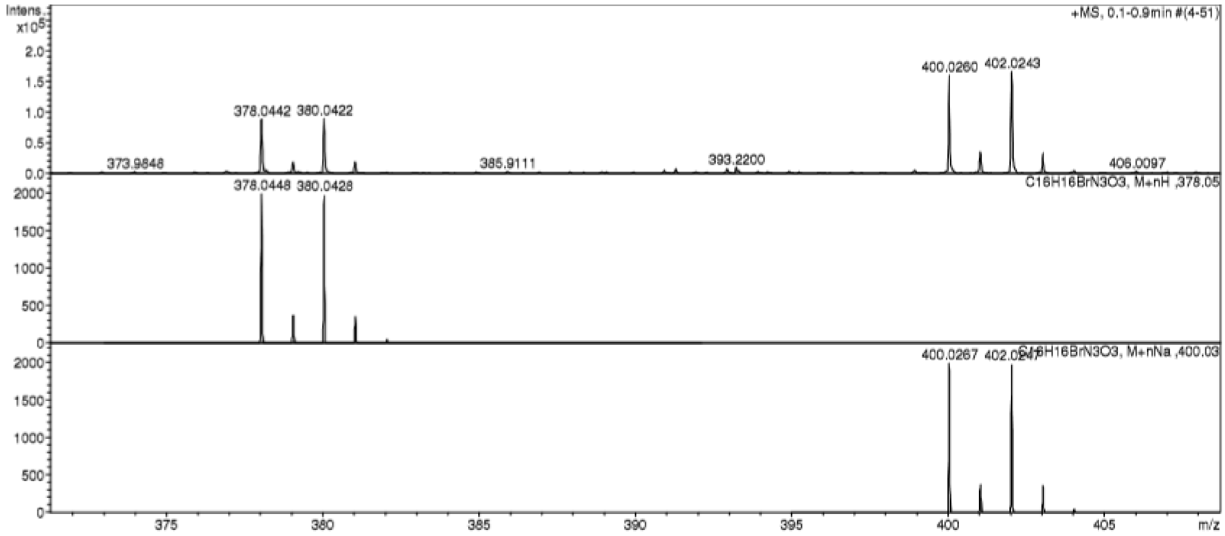

HRMS for **7d**

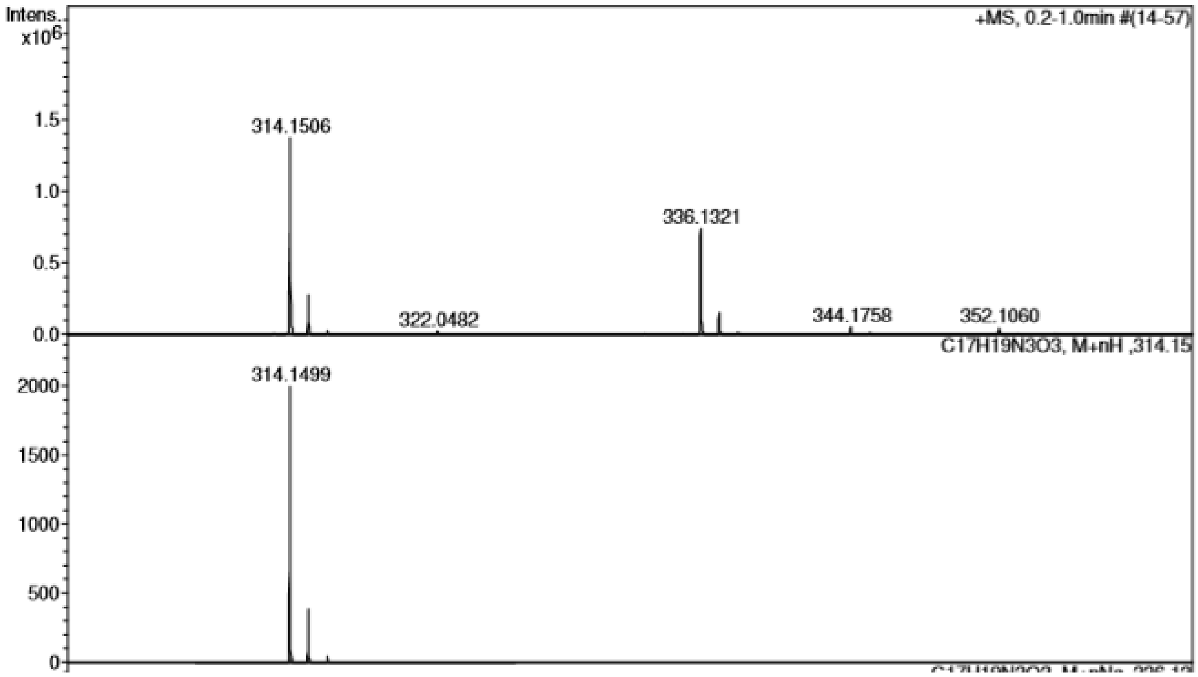

HRMS for **7e**

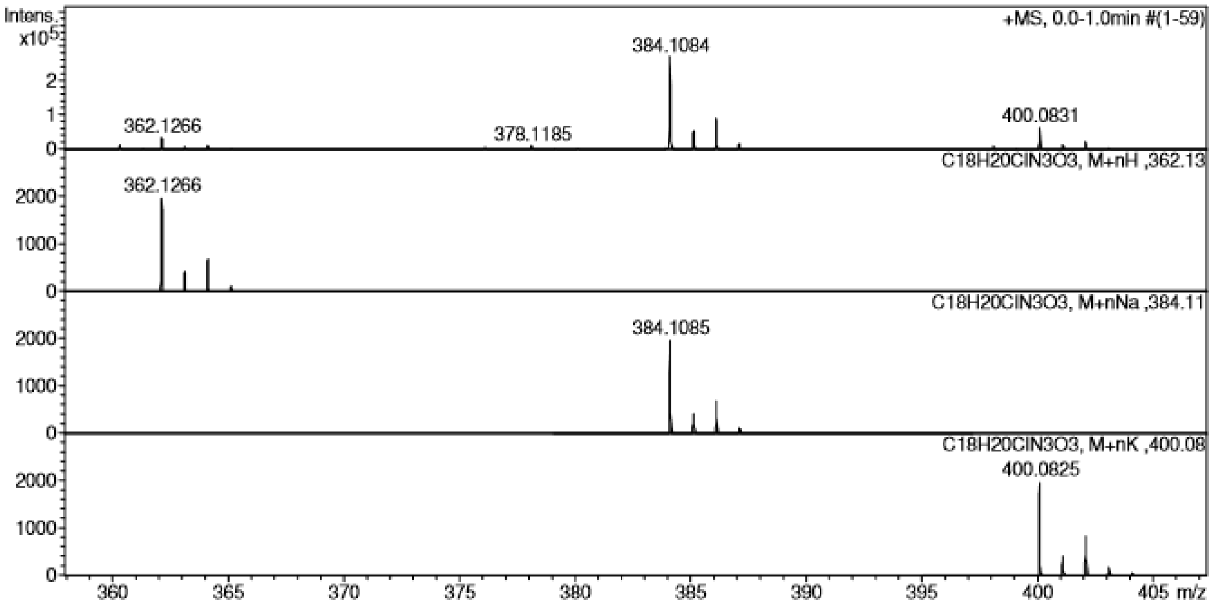

Intens.
x10^5
+MS, 0.0-1.0min #(1-59)
384.1084
362.1266
378.1185
400.0831
C18H20ClN3O3, M+nH ,362.13
362.1266
C18H20ClN3O3, M+nNa ,384.11
384.1085
C18H20ClN3O3, M+nK ,400.08
400.0825
m/z

HRMS for **7f**

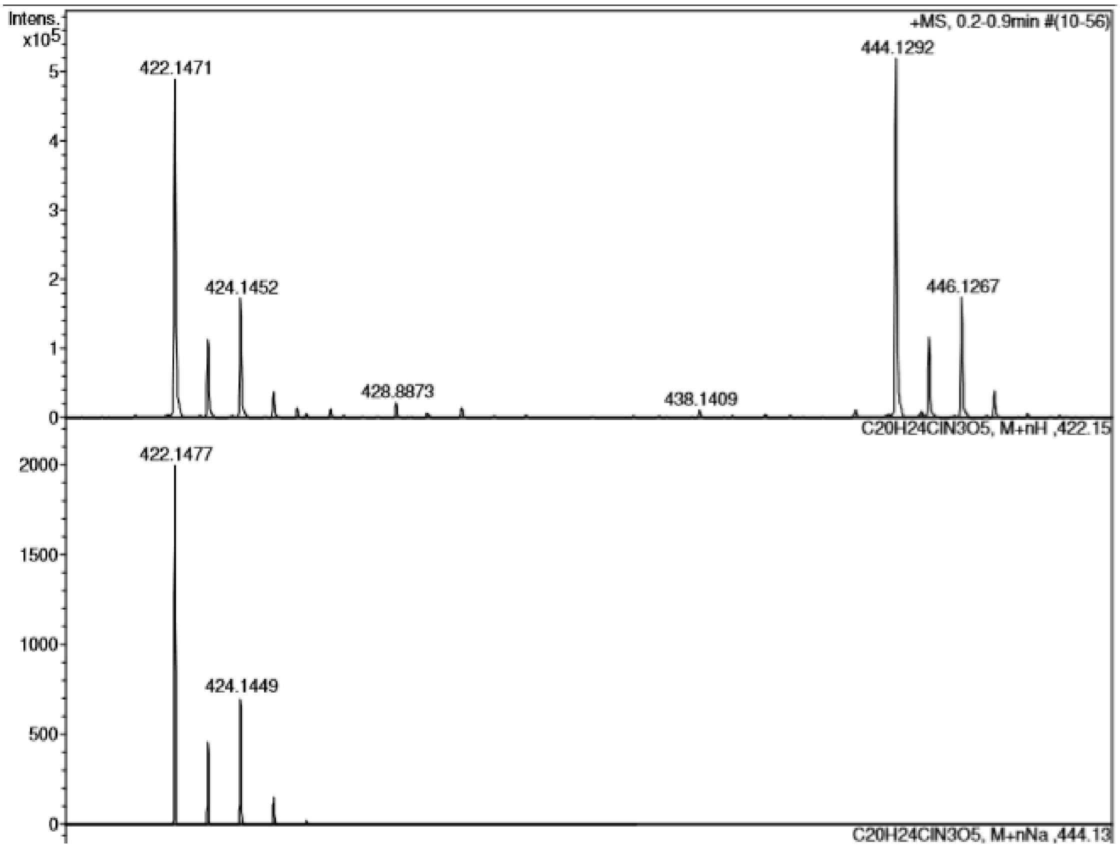

HRMS for **7a**

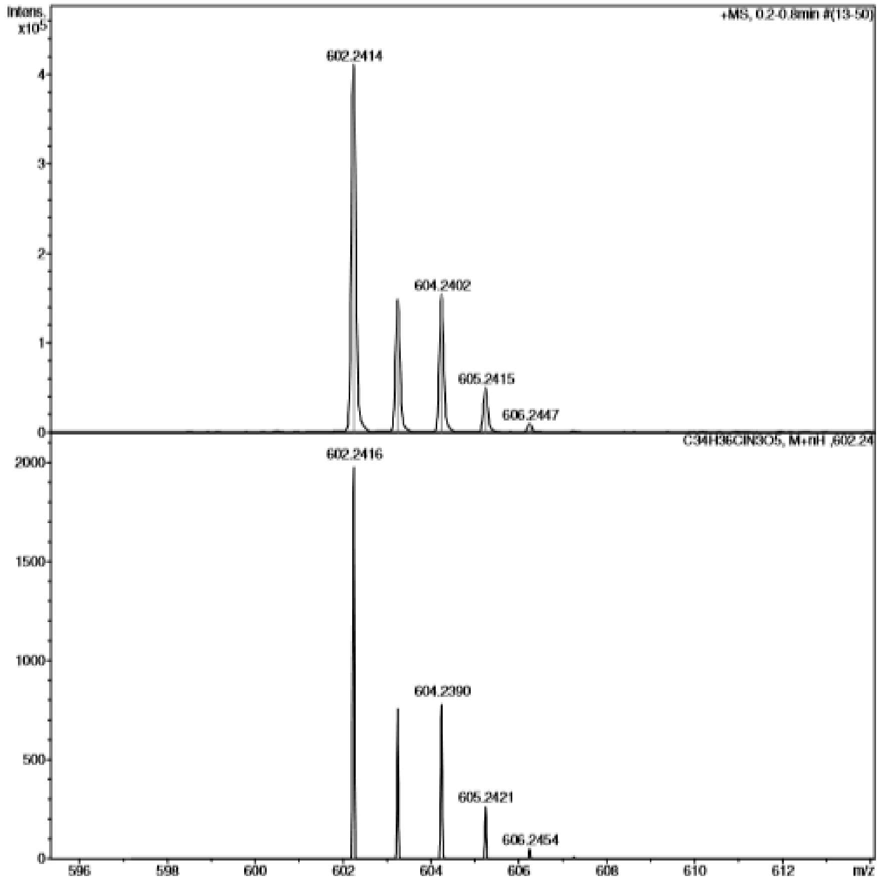

HRMS for **1b**

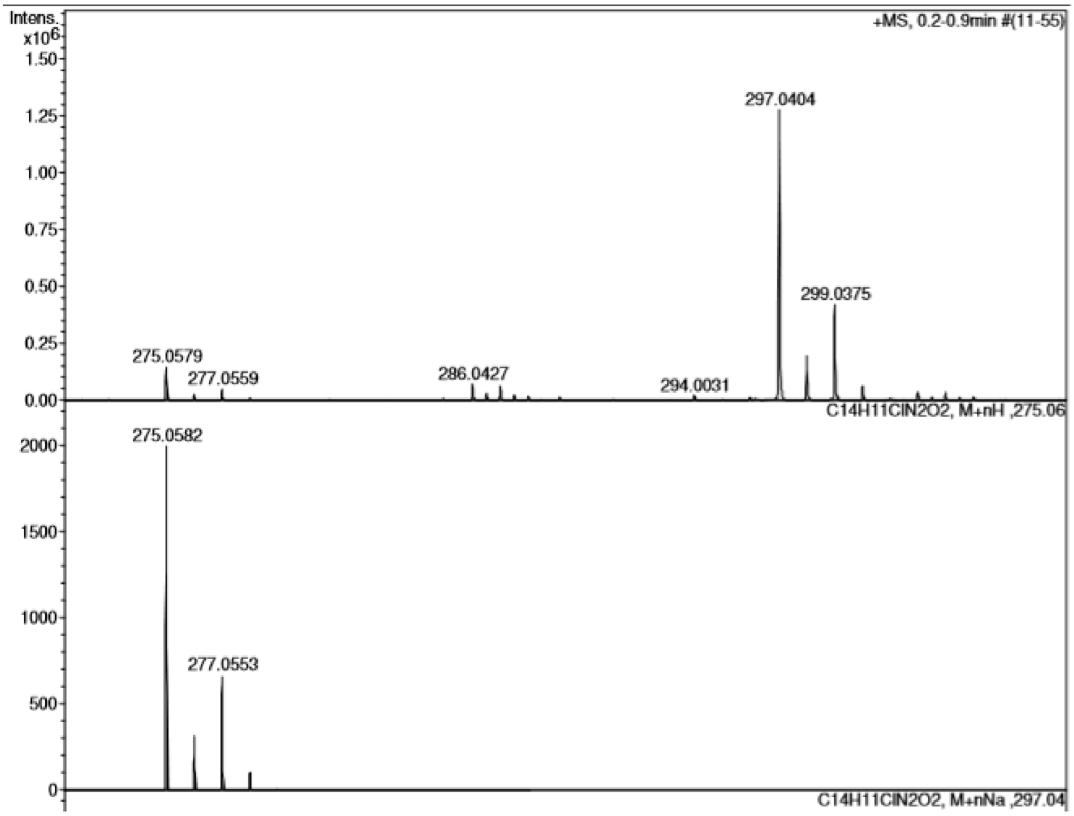

HRMS for **1c**

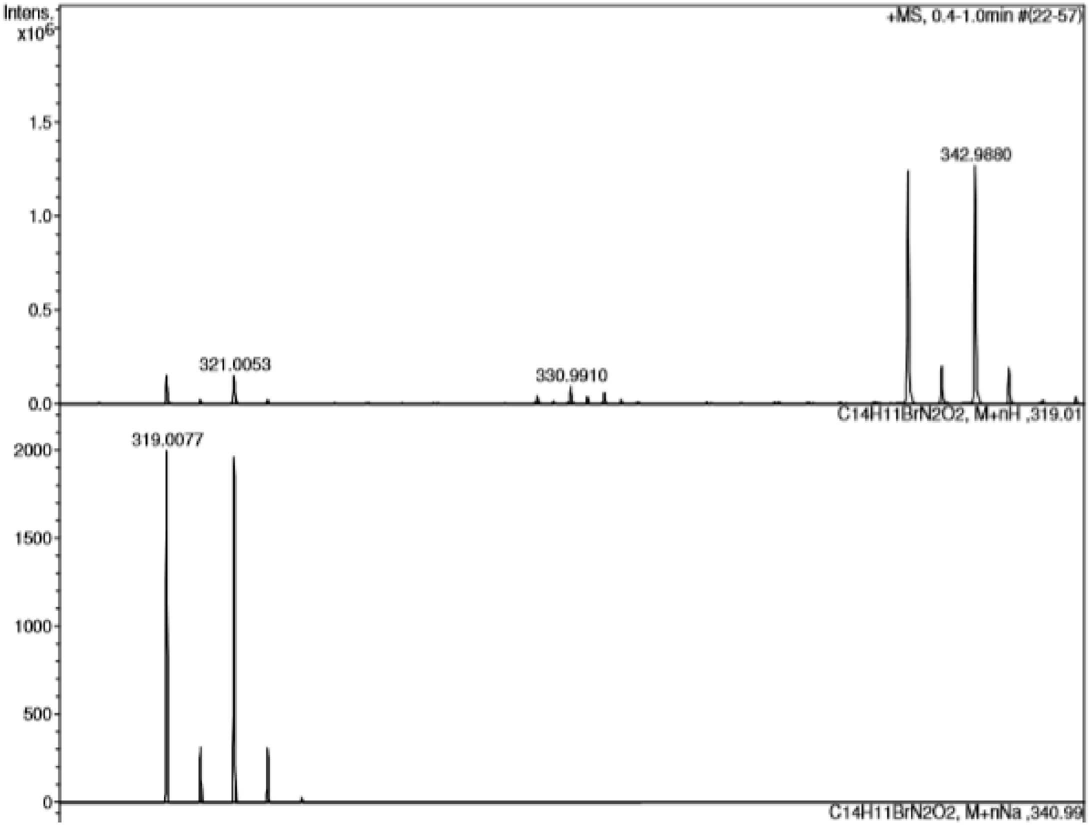

HRMS for **1d**

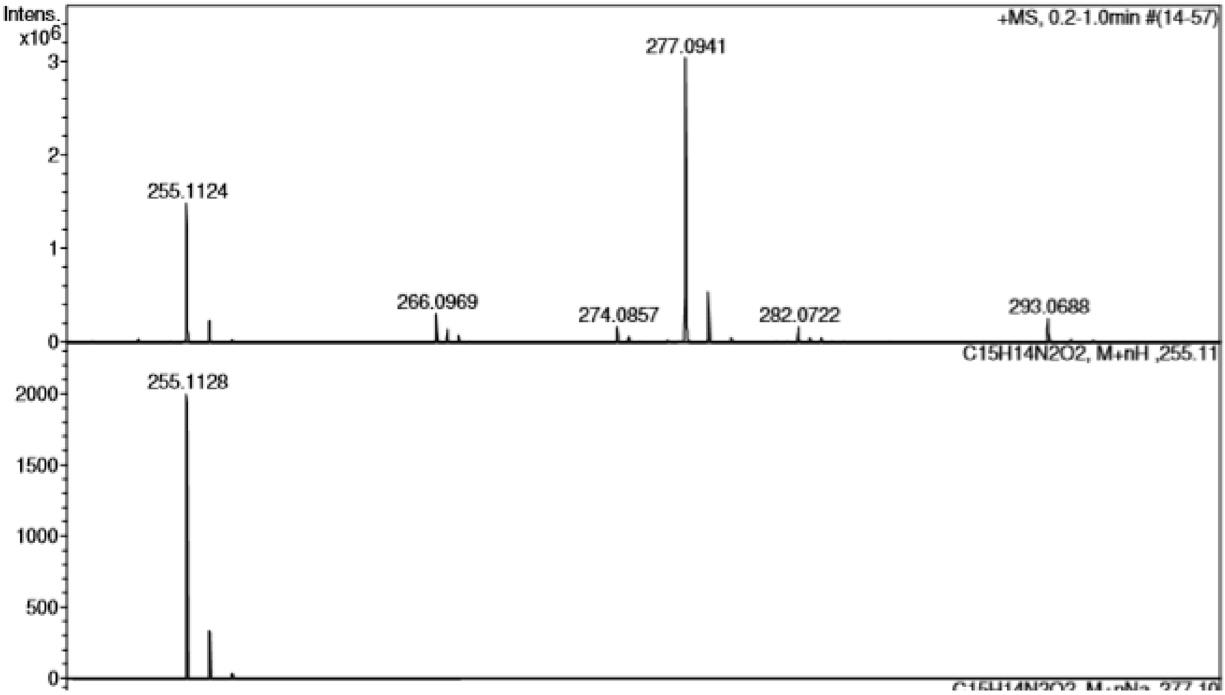

Intens.
x10^6
+MS, 0.2-1.0min #(14-57)
277.0941
255.1124
266.0969
274.0857
282.0722
293.0688
C15H14N2O2, M+nH ,255.11
255.1128

HRMS for **1e**

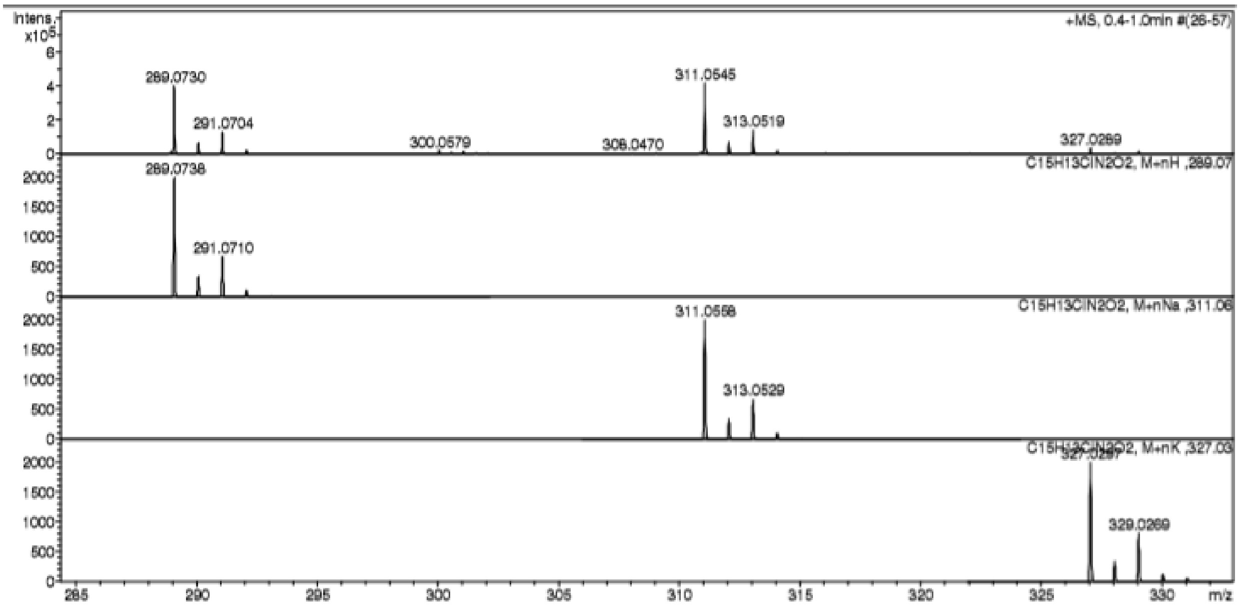

HRMS for **1f**

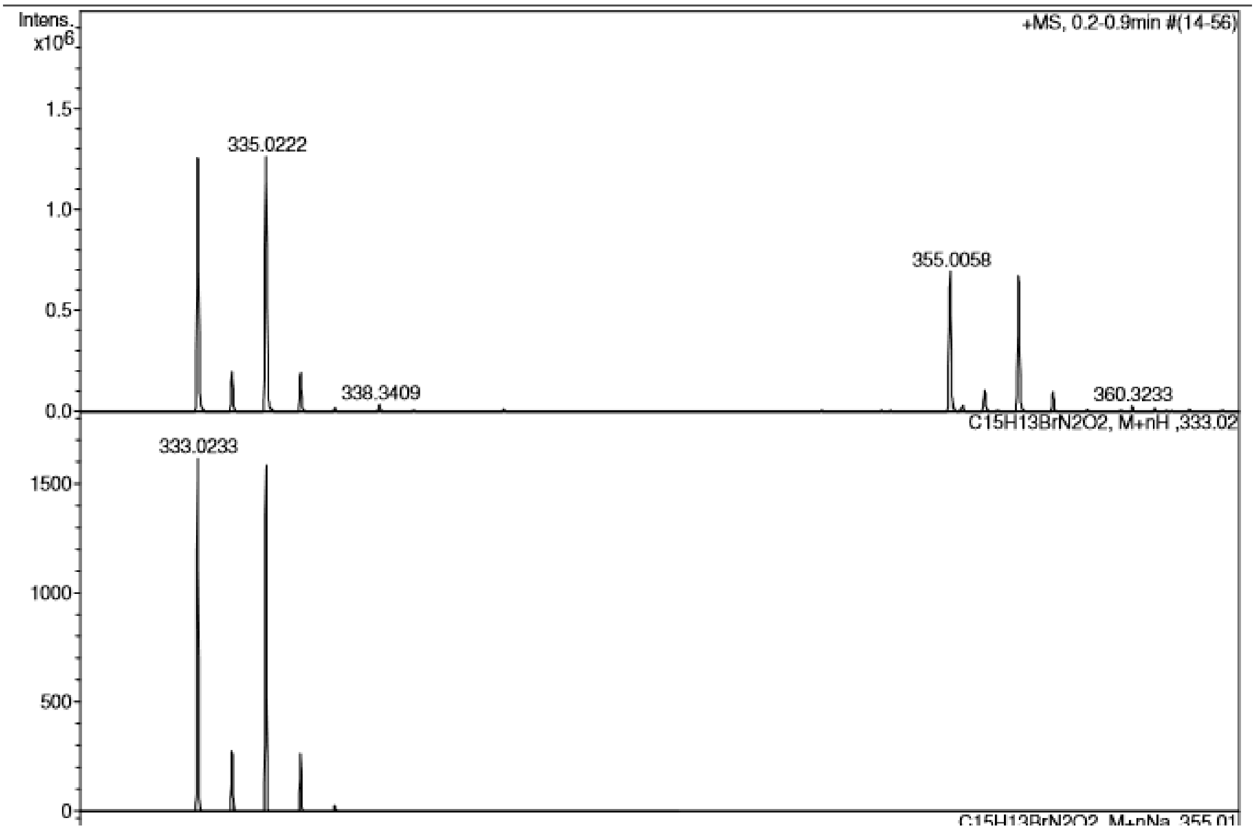

HRMS for **1g**

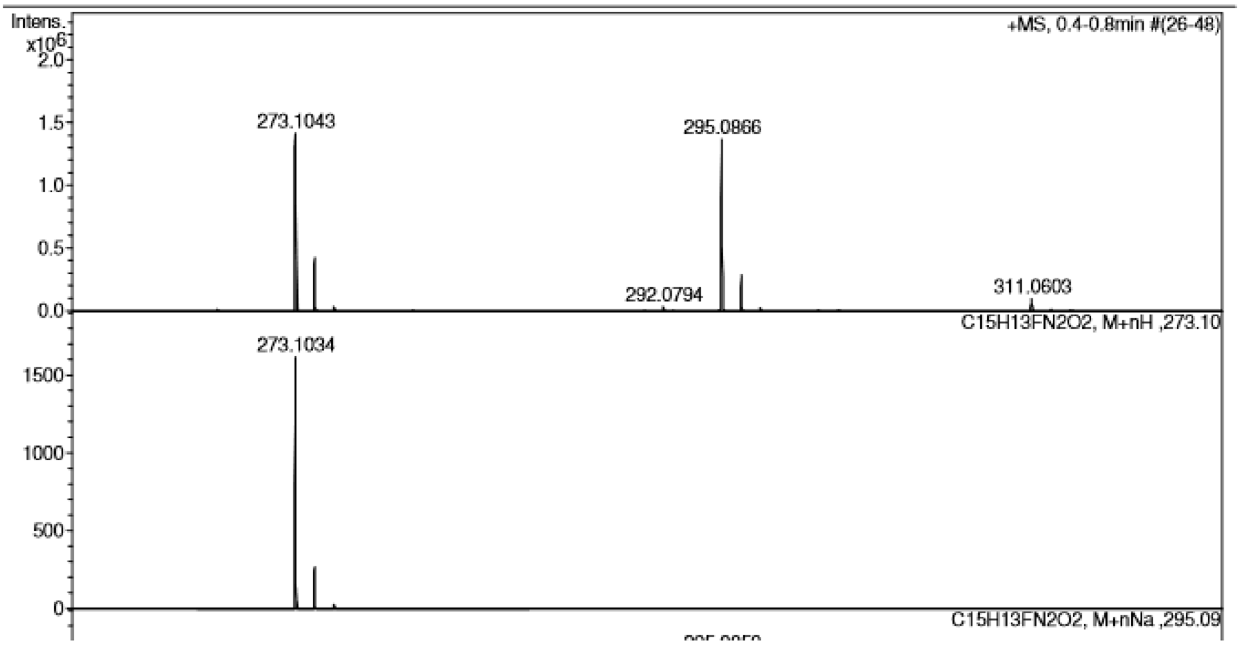

HRMS for **1h**

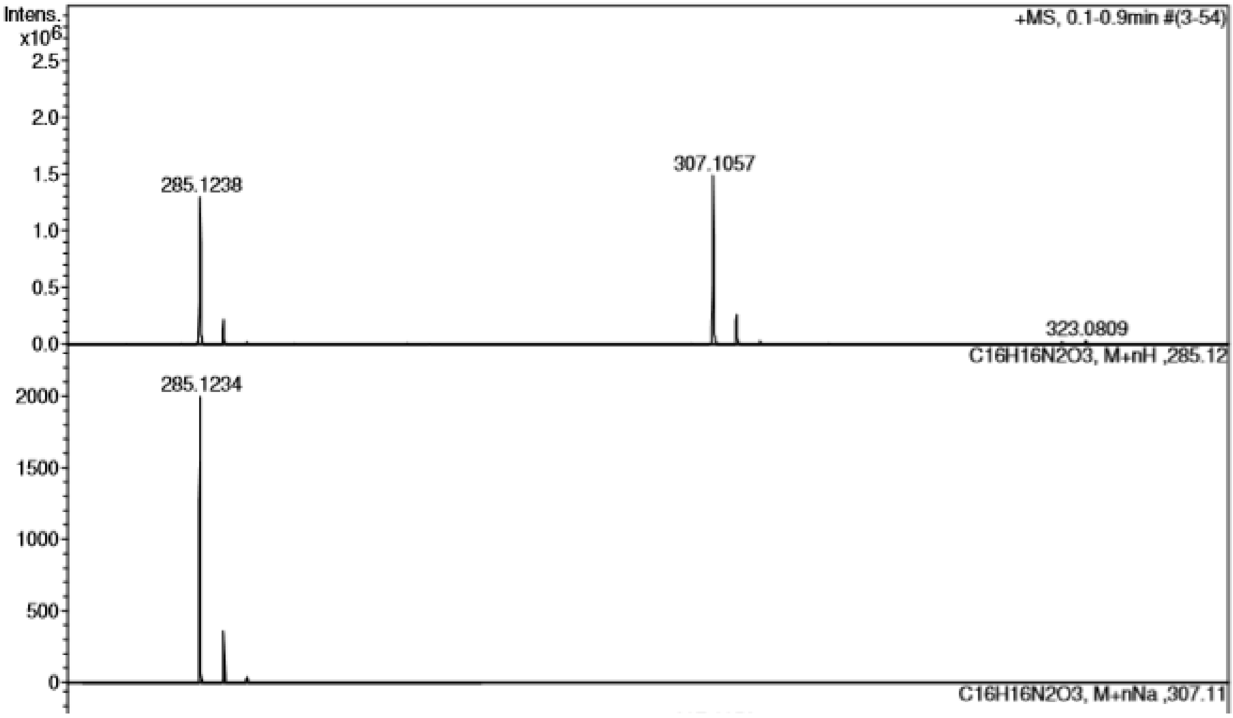

HRMS for **1i**

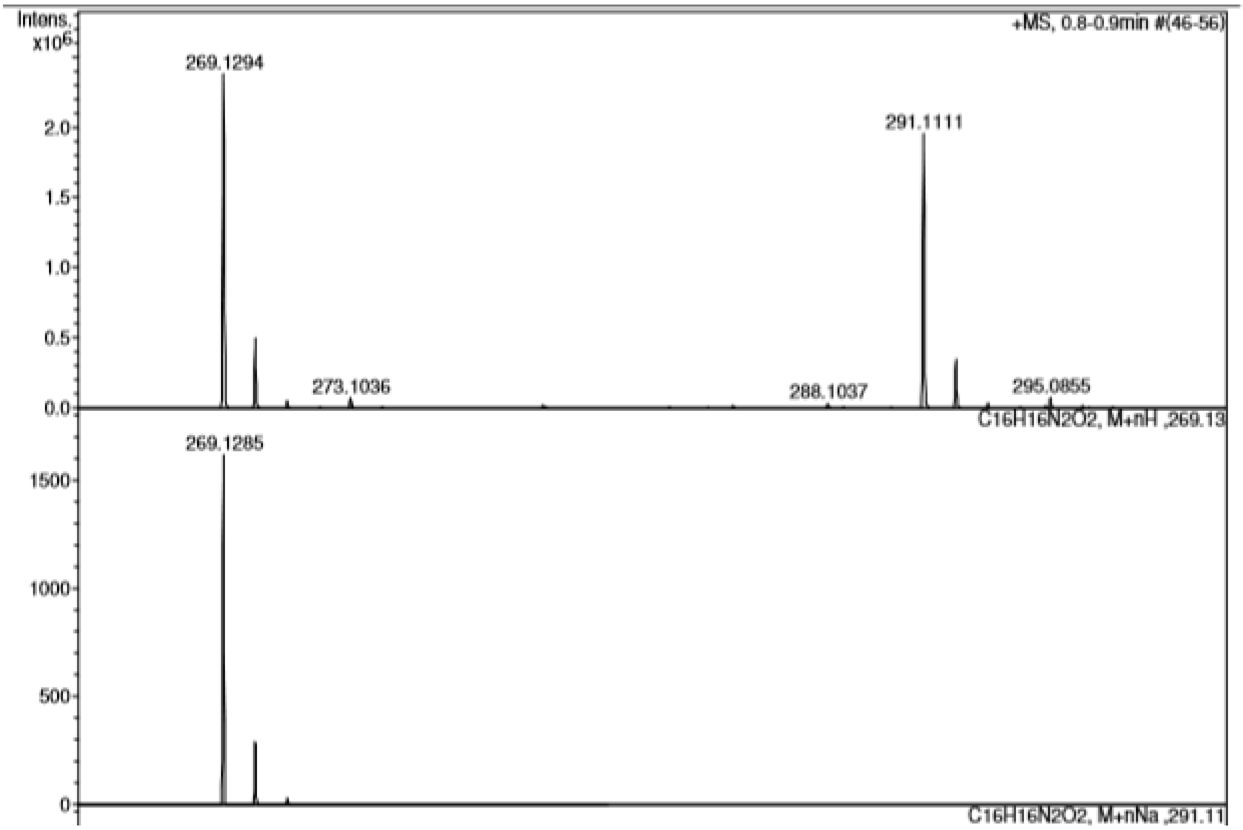

HRMS for **1j**

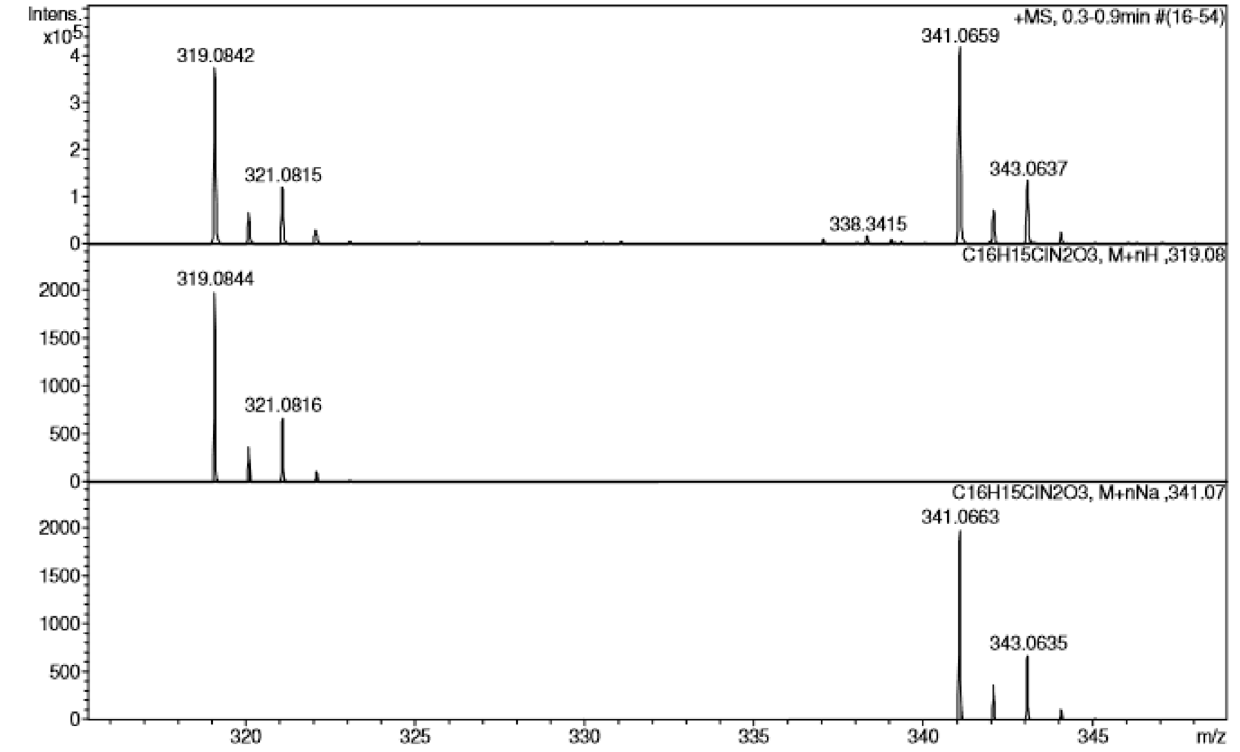

HRMS for **1a**

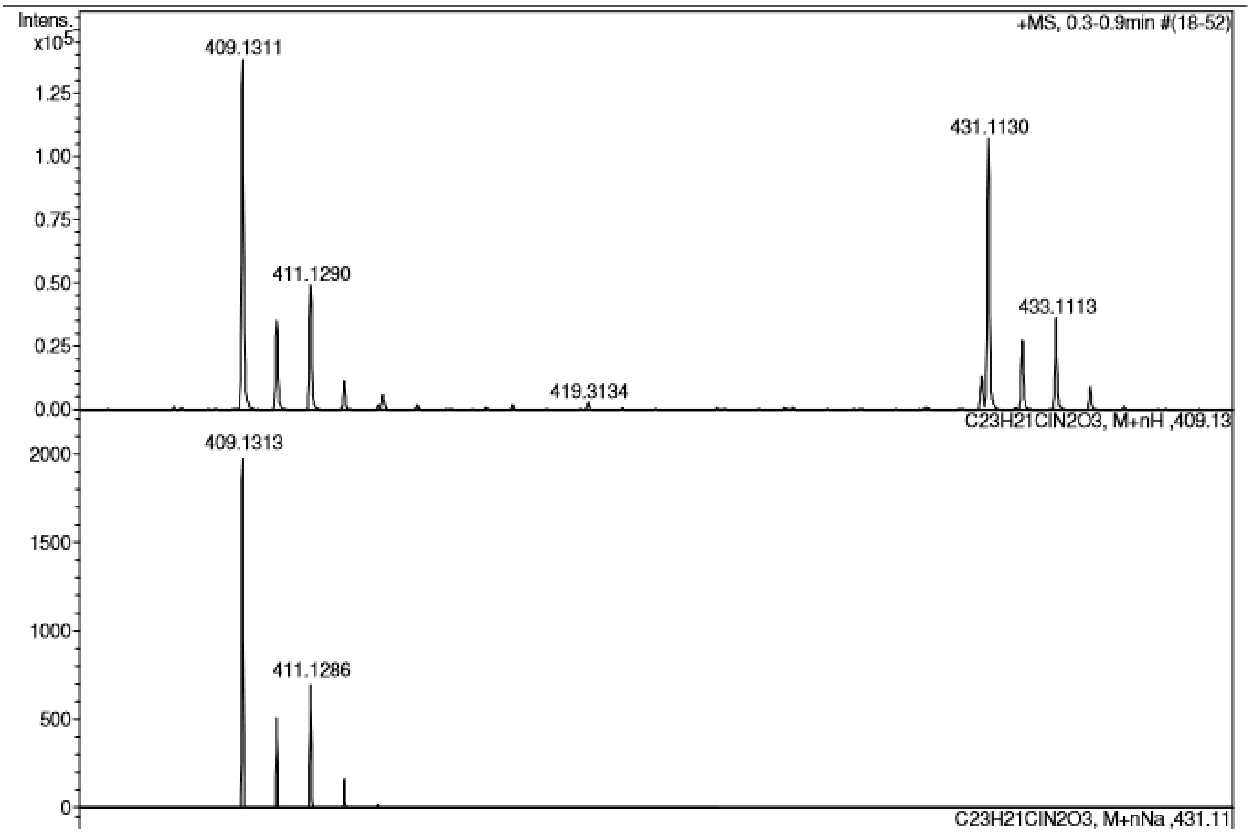

Intens.
x10^5
+MS, 0.3-0.9min #(18-52)
409.1311
431.1130
411.1290
433.1113
419.3134
1.25
1.00
0.75
0.50
0.25
0.00
C23H21ClN2O3, M+nH ,409.13
409.1313
411.1286
2000
1500
1000
500
0
C23H21ClN2O3, M+nNa ,431.11

6. Copies of 2D spectra

HMBC for compound **7e**

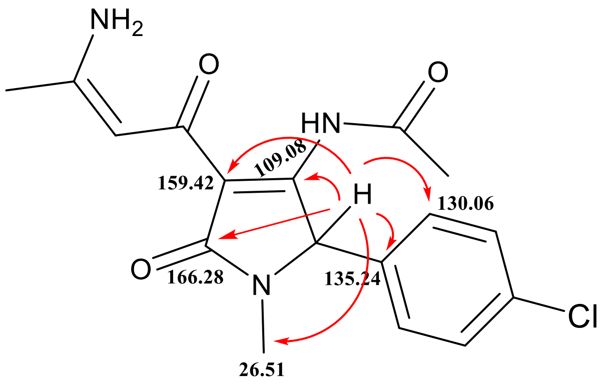

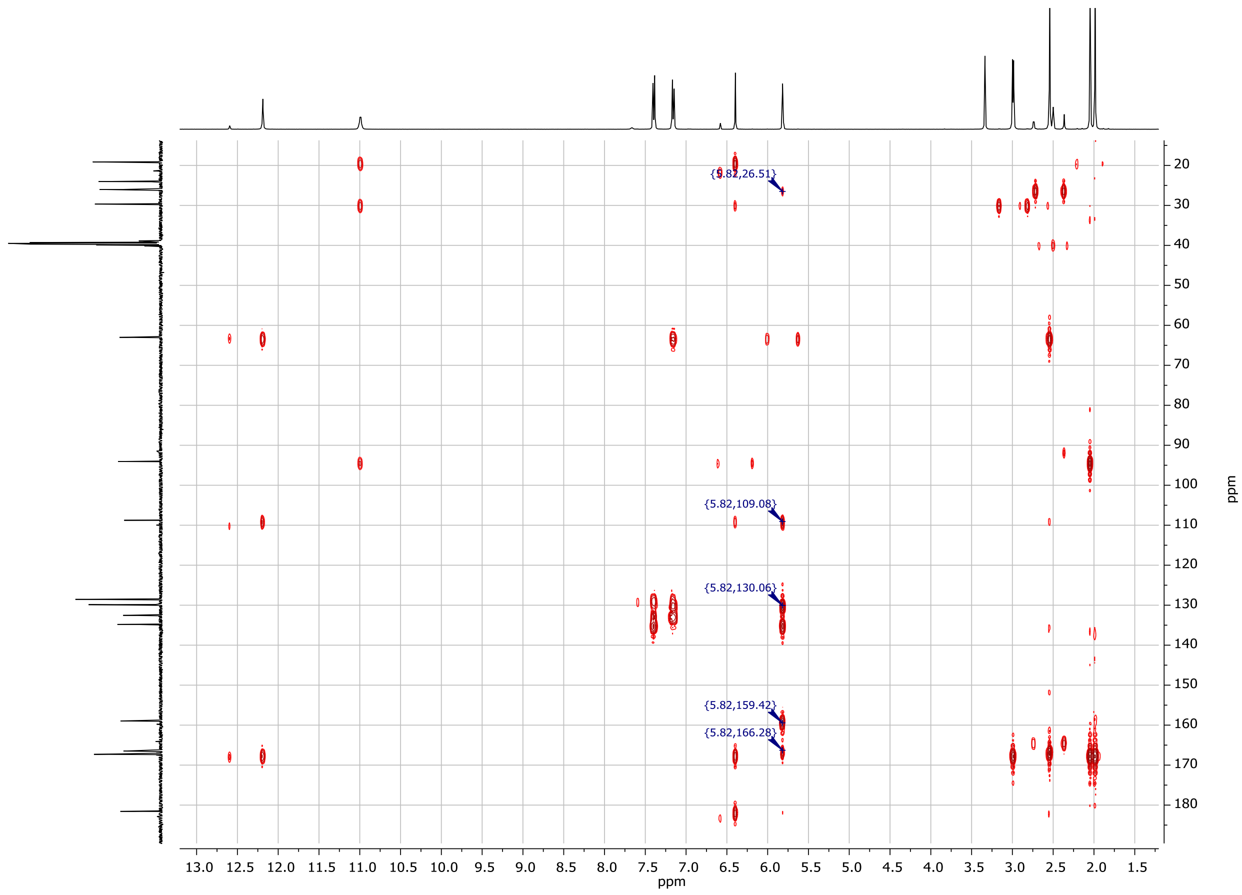

HMBC for compound **1e**

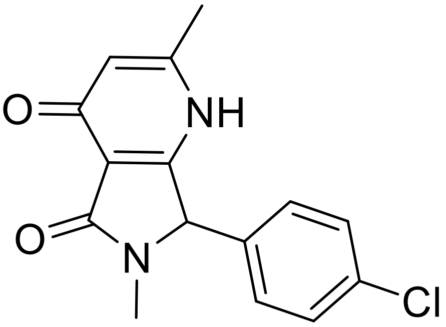

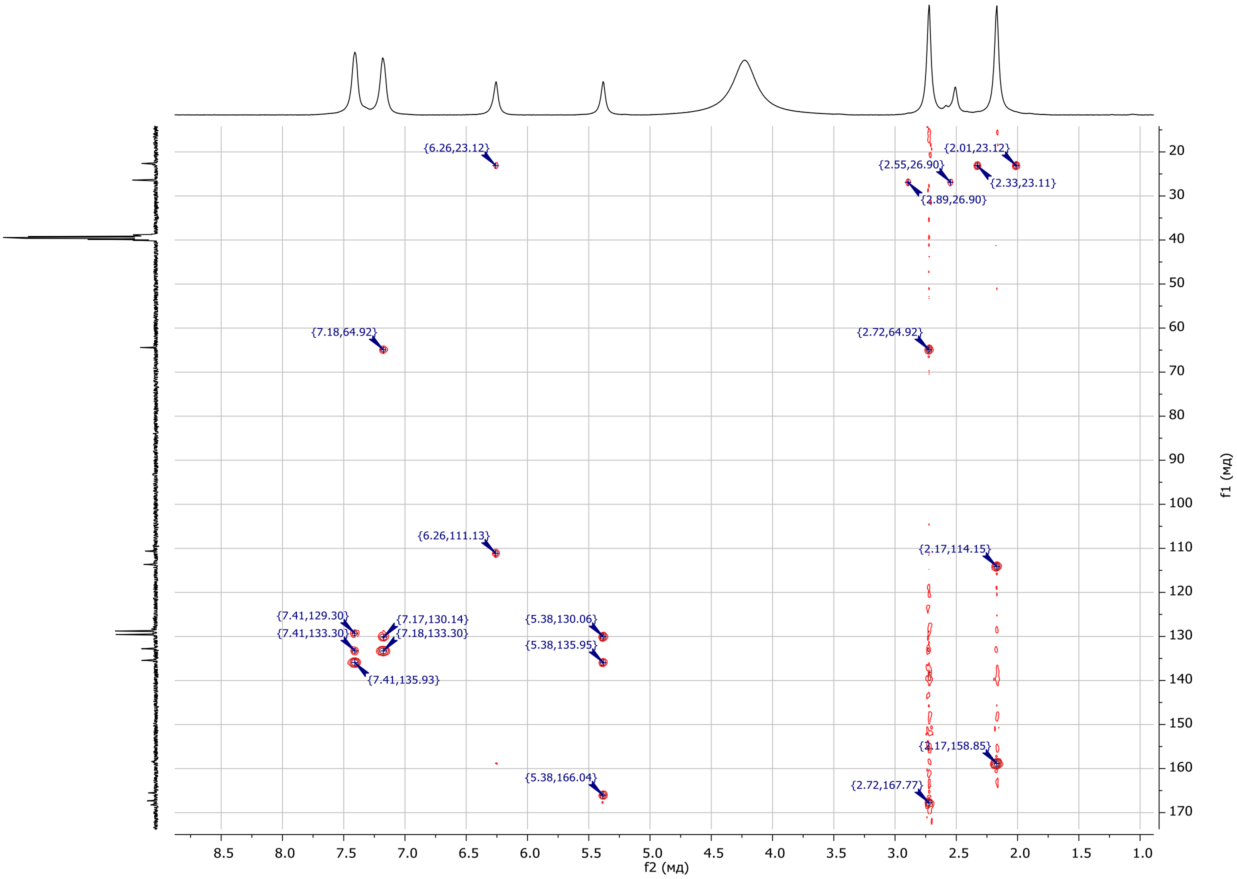

7. X-ray analysis of compound **1e**

Crystallographic data: Crystals of **1e** ($C_{17}H_{19}ClN_2O_3$, M = 334.79) are orthorhombic, space group *Pbca*, at 120 K: a = 15.8377(10), b = 12.1158(7), c = 17.2705(12) Å, V = 3314.0(4) Å^3, Z = 8 (Z' = 1), d_{calc} = 1.342 g cm^{-3}, μ(Cu Kα) = 21.82 cm^{-1}, F(000) = 1408. Intensities of 39379 reflections were measured with a Bruker APEX2 DUO CCD diffractometer [λ(Cu Kα) = 1.54178 Å, ω-scans, 2θ<135°], and 2914 independent reflections [R_{int} = 0.0403] were used in further refinement. The structure was solved by direct methods and refined by the full-matrix least-squares technique against F^2 in the anisotropic-isotropic approximation. Hydrogen atoms of the NH group and the OH group of the ethanol solvent molecule in **1e** were found in difference Fourier synthesis, while positions of others were calculated. All hydrogen atoms were then refined in the isotropic approximation within the riding model. The refinement converged to wR2 = 0.1187 and GOF = 1.043 for all the independent reflections (R1 = 0.0467 was calculated against F for 2439 observed reflections with I>2σ(I))**.** All calculations were performed using SHELXTL PLUS 5.0.[1] **CCDC 1921613** contains the supplementary crystallographic data for **1e**.

Reference

1. G.M. Sheldrick. A short history of SHELX. *Acta Cryst. A*, 2008, 64, 112-122
